# Supplementary material for: Genome-wide signatures of adaptation to extreme environments in red algae
Source: Nat Commun. 2023 Jan 4;14:10. doi: 10.1038/s41467-022-35566-x (PMC9812998; doi:10.1038/s41467-022-35566-x)
Supplement: Supplementary file 1 — Supplementary Information [file 41467_2022_35566_MOESM1_ESM.pdf]

Supplementary materials for

Genome-wide signatures of adaptation to extreme environments in red algae

Chung Hyun Cho[1], Seung In Park[1], Tzu-Yen Huang[1], Yongsung Lee[1], Claudia Ciniglia[2], Hari Chandana Yadavalli[3], Seong Wook Yang[3], Debashish Bhattacharya[4], Hwan Su Yoon[1,*]

[1]Department of Biological Sciences, Sungkyunkwan University, Suwon 16419, Korea

[2]Department of Environmental, Biological and Pharmaceutical Science and Technologies, University of Campania Luigi Vanvitelli, Caserta, Italy

[3]Department of Systems Biology, Institute of Life Science and Biotechnology, Yonsei University, Seoul, Korea

[4]Department of Biochemistry and Microbiology, Rutgers University, New Brunswick, USA

[*]Corresponding author: E-mail: hsyoon2011@skku.edu

Supplementary Notes 1-8

Supplementary Figures 1-24

Supplementary References

Supplementary Notes

Supplementary Note 1: Issues with previous genomes and gene modeling

When compared to sister species in the Cyanidiales, the *Cyanidiococcus yangmingshanensis* THAL066 genome contains a larger number of introns (453), which is 12.6x higher intron abundance than in the 8.1.23 F7 strain. Moreover, THAL066 appears to have a higher number of protein-coding genes (5,189) than other Cyanidiales species (4,803-4,870) including the genomes from this study. We investigated whether the higher number of introns and genes was explained by strain-specific evolution or alternatively, due to mistakes in gene modeling. We compared gene synteny because genome structure of the two *Cyanidiococcus* strains is highly conserved (Supplementary Fig. 1). We compared a region that has high nucleotide conservation, but different gene structure (i.e., exon-intron pattern) in the two *Cyanidiococcus* genomes. Due to the "unpolished" genomes and gene modeling issues, particular genes from THAL066 were misinterpreted (Supplementary Fig. 2a). For example, the OCA6 gene is located in chromosome 20 of the THAL066 genome, but those genes were not found in other sister species (Supplementary Fig. 2a). The intron region of the OCA6 gene in THAL066 has four nucleotide substitutions when compared to the coding region of the OCA6 gene in the 8.1.23 F7 strain, and these changes are explained by frameshifts (Supplementary Fig. 2a). Iso-Seq transcripts from 8.1.23 show no evidence of intron splicing in the OCA6 gene and we confirmed that the extra introns in the THAL066 genome were overrepresented, with a similar pattern to that of OCA6 gene. Similarly, 'F1559_002061' and ‘F1559_002077’ genes in THAL066 strain were matched to the 5' and 3' ends of another Cyanidiales gene (e.g., ‘CCYA20G4772’ gene of *Cyanidiococcus* 8.1.23 F7, ‘CMA118C’ gene of *Cyanidioschyzon* 10D). The nucleotide alignment of ‘CCYA20G4772’ gene (1,638 bp) in 8.1.23 F7 strain revealed only four nucleotide substitutions (99.8% identity) when compared to corresponding region of THAL066 strain (Supplementary Fig. 2a). As a result, we were able to determine that the stop codon was derived from a single nucleotide insertion in ‘F1559_002061’ gene when compared to our 8.1.23 F7 genome with Iso-

Seq transcript verification. Sequence variation split a single gene into two different genes in the THAL066 strain, and gene family analysis revealed that duplicated genes of THAL066 strain found in other Cyanidiales single gene-containing orthogroup were caused by this case. All these data suggest that the THAL066 genome contains mis-annotations with respect to introns due to issues with gene prediction that are explained by incomplete genome correction and intron prediction problems.

In the case of *Galdieria sulphuraria* 074W, this genome has two issues: viral contamination and a chimeric assembly. Because of its viral gene content (five viral protein hits from contigs) and genome features (e.g., short intergenic space), 'scaf_102' from the 074W genome is a viral contig that is not present in any other *Galdieria* assembly (Supplementary Fig. 2b). Furthermore, we discovered a long telomere region in 'scaf_14' that was linked to another gene region by a 1.5 kbp gap ('N'), indicating a chimeric scaffold. Considering all of these issues, we decided to use only a small number of high-quality genomes (e.g., telomere-to-telomere, T2T, or similar quality) to interpret gene evolution and genomic features in our study.

Supplementary Note 2: Telomeres of cyanidiophycean species

The basic structure of telomeric repeat sequences in *Cyanidiococcus* and *Cyanidioschyzon* is 'A_2TG_6' (9 mers), but *Cyanidium* contains one additional guanine sequence as 'A_2TG_7' (10 mers). Using the reference *Galdieria* genomes and newly assembled *Galdieria* 108.79 E11 genome, we found that the telomeric repeats in the genomes of *Galdieria sulphuraria* (GASU) and *Galdieria phlegrea* (GAPH) are '$T_2AG_3T_3A$' (10 mers). Galdieriales telomeric repeats share higher similarity with these regions in other lineages (e.g., 'T_3AG_3': *Arabidopsis* and most of land plants, 'T_2AG_3': the most common telomeric repeats found across the tree of life including human) than with Cyanidiales species [1-3]. This implies that, similar to other lineages, Galdieriales has additional 'T_3A' sequences from the probable ancestral telomeric sequence ('T_2AG_3'), whereas Cyanidiales species have two distinct telomere features: an extended poly-G sequence

and 'A$_2$T' sequences before the poly-G sequence (A$_2$TG$_6$ in *Cyanidiococcus* and *Cyanidioschyzon*; A$_2$TG$_7$ in *Cyanidium*), which are not commonly found in telomere sequences of other species. The modification of the poly-G sequence in Cyanidiales telomeres may affect the formation of the G-quadruplex structure (a DNA secondary structure), potentially preventing telomeric functions (e.g., telomere elongation) [4].

The G-quadruplex typically generates CD spectra with a negative peak at 245 nm and a positive peak at 265 nm according to previous CD spectra results [5]. To test this further, we measured CD spectra of telomeric repeats using synthesized oligonucleotides and compared telomeric repeats of cyanidiophycean algae with the G-quadruplex forming positive controls (i.e., G-rich strand of human and *Arabidopsis*) and negative controls of non-G-quadruplex forming telomeric repeats (i.e., C-rich strand of human and *Arabidopsis*) (Supplementary Figs. 3, 4; Supplementary Dataset 2). Despite having completely different telomeric sequences when compared to the reference samples (e.g., Hsa_G4, Ath_G4, G4T4), our CD results revealed a clear, typical pattern of positive and negative peaks in both Cyanidiales and Galdieriales (Supplementary Fig. 3). This result suggests that telomeres in Cyanidiales and Galdieriales evolved differently in each lineage but form a G-quadruplex sequence at the end of telomere, similar to other eukaryotes.

Unlike telomeric repeats, *Cyanidioschyzon* centromeres are non-repetitive and heterogeneous DNA elements that lack inter-chromosomal sequence similarities. We were not able to identify the putative centromeric regions from other Cyanidiales genomes using our methods [6,7].

Supplementary Note 3: Assessment of the *Galdieria sulphuraria* 108.79 E11 genome

We discovered that all publicly available 11 *Galdieria* genomes, including both *G. sulphuraria* (GASU) and *G. phlegrea*, have identical telomeric repeat sequences ('T$_2$AG$_3$'). The newly sequenced GASU 108.79 E11 genome has 58 chromosomes with both telomere regions and 16

scaffolds with a single telomere region indicating that around 66 chromosomes exist in this strain. Among the 11 published *Galdieria* genomes, the GASU MtSh genome [8] has a higher quality (e.g., number of scaffolds, N50 of scaffolds, BUSCO). Eighty scaffolds from the MtSh genome have telomere sequences at one end with 36 scaffolds having telomeres on both ends (58 chromosomes: 36 complete + 22 single-end, 22 without telomeres, see Supplementary Dataset 3). The MtSh strain has the same estimated chromosome number as 108.79 E11 strain (76 scaffolds: 58 complete + 16 single-end, 2 without telomeres) (Supplementary Fig. 5), and this number is comparable to chromosome numbers (57 chromosomes from RT-M9 and RT-1.1 strains) from previous work [9,10].

Supplementary Note 4: *Ab initio* prediction of miRNA in Cyanidiophyceae

The presence of miRNA is provisional when using *in silico* prediction due to the lack of experimental verification [11,12]. Using the most recent Rfam database (v14.5), a few miRNA candidates from Cyanidiophyceae genomes were identified with low homology (Supplementary Dataset 10). By applying a strict *e*-value cut-off value (<0.001), less than four miRNA candidates in each species remained and no overlapping miRNA between species was observed. Due to the loss of key miRNA pathway components, miRNA validation with probes will be required in the future.

Supplementary Note 5: Long noncoding RNA (lncRNA) in Cyanidiophyceae

Unlike assembled transcriptome data from Illumina sequencing, PacBio Iso-seq data allowed us to identify full transcripts and coding direction of genes [13]. Most transcripts aligned well with their transcriptional direction; however, some transcripts were mapped in the opposite direction, partially or completely covering the gene (Supplementary Fig. 16a). Those transcripts can be classified into different types of mapping patterns, i) gene-independent antisense transcript, and ii) gene-containing antisense transcript, which was transcribed from the adjacent gene that was in

opposite direction (Supplementary Fig. 16b). Although most long noncoding RNA (lncRNA) candidates overlapped only a partial region of corresponding genes, we were able to find a few lncRNA candidates that spanned the complete gene region (Supplementary Fig. 16). We suggest these transcripts in *Cyanidiococcus* and *Cyanidium* are the lncRNA candidates, which are similar to the report from brown algae and green algae [14,15]. As a result, we propose that the small RNA interference system has degenerated in Cyanidiales, but lncRNAs may be able to regulate transcription, splicing, and translation.

Supplementary Note 6: Polycomb complexes in Cyanidiophyceae

Polycomb groups (PcG) are a well-preserved system for epigenetic regulation (i.e., post-translational modifications) from the eukaryotic ancestor, and they have been highlighted for actively interacting with histones to repress transcription [16]. Two major polycomb repressive complexes (PRCs) have been proposed in plant and algal systems: PRC1(-like) complex that ubiquitinates histone 2A (H2AK119) and PRC2(-like) complex that methylates histone H3 (H3K27) [17,18]. Based on previous polycomb protein surveys [18,19], we discovered that Cyanidiophyceae retained the core PRC2 genes from eukaryotic ancestor, but the core PRC1 proteins were degenerated in the red algal ancestor (Supplementary Fig. 17). The BMI1 gene, which is one of the core PRC1 components, was discovered in Galdieriales, and phylogenetic analysis of BMI supports the idea that red algae degenerated these genes independently in each Rhodophytina and Cyanidiales lineages (Supplementary Fig. 17). This implies that, while the PRC2 system has been preserved, the PRC1 system in red algae has degenerated and may have lost or replaced histone H2AK119 mono-ubiquitination, which is required for polycomb-mediated transcription regulation.

Supplementary Note 7: Heat shock protein-related pathway and protein properties of Cyanidiophyceae

Heat shock proteins (Hsp; chaperones) are the most well-known proteins involved in protein homeostasis that react to heat stress or other external stresses [10,20]. We identified different chaperone types (Hsp20, 40, 60, 70, 90, and 100) from our representative taxon sampling (Supplementary Fig. 18). Although cyanidiophycean are extremophiles, the number of chaperon genes doesn't show any difference with other mesophilic red algae (Cyanidiales: 55-60, Galdieriales: 61-74, Rhodophytina: 58-71). A single *dna*J gene (Hsp40), which functions as co-chaperones and nucleotide exchange factors for Hsp70s [21], was identified as a HGT candidate gene in Cyanidiales, chaperone proteins analysis revealed no significant traits of extremophiles without it. After that, each proteome's aggregation propensity was assessed and compared, because chaperones preferentially bind to aggregation-prone regions (APRs) of proteins [22,23]. We grouped aggregation values of each proteome based on classification system (minimum class level) to see the differences (Supplementary Fig. 19). Extremophilic cyanidiophycean species have more aggregation prone regions than other lineages and statistical test supports that they have significantly different (t-test p-value < 0.05) proteome aggregation scores compared to mesophilic red algae (average of Cyanidiophyceae: 2,749.5; average of Rhodophytina: 2,202.4). The hydrophobicity of proteomes was not significantly different between lineages.

We hypothesize that the proteostasis machinery of cyanidiophycean species has undergone specific adaptations to control their highly aggregation-prone proteome by removing misfolded and aggregated proteins. This process allows Cyanidiophycean species to provide degradation systems that prevent proteins from malfunctioning due to extreme environmental stress, which is similar to the conclusion reached about Galdieriales mitochondrial-encoded proteins [24].

Unlike non-amplified copies of chaperones in Cyanidiophyceae, heat shock transcription factors (HSFs) have been duplicated in all cyanidiophycean algae (Cyanidiales: 3 copies, Galdieriales: 3-4 copies), in contrast to the other mesophilic red algae that has a single copy [25]. HSFs are transcription activators of heat shock related-encoding genes (e.g., heat shock protein)

that bind to heat shock promoter elements and these genes have been amplified significantly in land plant lineages [26,27]. Not only have HSFs been linked to heat stress, but also response to variable environmental stresses (e.g., high light, drought, chilling stress, ROS), as well as plant cadmium tolerance [28,29]. According to previous phylogenetic analysis of HSF genes, multiple duplication events (but not caused by subtelomeric gene duplication event) of HSF genes (one during the divergence of Cyanidiales and Galdieriales, and two in Galdieriales) have the potential to benefit polyextremophilic adaptation of Cyanidiophyceae despite the genome reduction event at the same time [25].

Supplementary Note 8: Evidence of polycistronic gene expression in Cyanidiophyceae

The translation of multiple proteins from a single transcript that is clustered into operons is referred to as polycistronic gene expression and this system is widespread in prokaryotic genomes that often encode biosynthetic gene clusters [30]. Although there were reports of polycistronic gene expression in a few green algae and land plant species, it has not been reported in any red algae and most of eukaryotic lineages [31-33]. From the Illumina assembly data of transcriptome, we discovered that some transcripts are co-expressed multiple genes in the same transcript. To verify whether these transcripts were artificially created during assembly or was a true polycistronic transcript, assembly-free, full-length cDNA PacBio Iso-Seq was used. Similar to polycistronic transcripts identified from Illumina assembly data, a total 470 (705 genes) out of 16,029 Iso-Seq transcripts from *Cyanidium,* 334 (478 genes) out of 20,488 from *Cyanidiococcus* Iso-Seq transcripts, and 408 (852 genes) out of 21,609 from *Galdieria* Iso-Seq transcripts were identified to be polycistronic transcripts. Custom primers for polycistronic candidates and Sanger sequencing after PCR confirmed these polycistronic gene expressions in *Cyanidium* (Supplementary Fig. 20; Supplementary Dataset 11).

Most of polycistronic transcripts were bicistronic [*Cyanidium*: 442 out of 470 transcripts (94.0%), *Cyanidiococcus*: 328 out of 334 transcripts (98.2%)] in Cyanidiales, but some contained

more than three or more colinear genes (up to six colinear genes in *Cyanidium*). Unlike the other two Cyanidiales species, *Galdieria* has a lower proportion of bicistronic transcripts [171 out of 470 transcripts (36.4%)] and a higher proportion of polycistronic transcripts with more than two colinear genes in a single transcript (up to 14 colinear genes). Significant functional correlation among co-localized genes was found from functional annotation information of polycistronic transcripts (*Cyanidium*: 22 out of 705 co-expressed genes, *Cyanidiococcus*: 12 out of 478 co-expressed genes).

To analyze polycistronic transcription in our study species, we first tried to identify internal ribosome entry sites (IRESs), which can initiate protein translation from the mRNA intergenic region among genes. None of the polycistronic transcripts in Cyanidiales are predicted to contain IRES, ruling out the possibility of polycistronic transcriptions mediated by IRES as found in *Chlamydomonas* [33]. On the other hand, *Galdieria* polycistronic transcripts have been predicted to provide IRES signal [380 out of 408 transcripts (93.1%)], which remains the IRES polycistronic transcription mechanisms developed in *Galdieria* species.

No homologous sequences were detected in the intergenic region of polycistronic genes using 2A self-cleaving peptides, which are ~30 amino acids long and contain the conserved sequence motif ('-DxExNPGP-') that could be one of the polycistronic transcription methods [34]. We were unable to demonstrate other polycistronic expression mechanisms such as 'termination-reinitiation' and 'leaky scanning' in the green algal cases with our current results [33]. Multiple surveys of polycistronic mechanism reveal that the two lineages may have developed completely different transcriptional mechanisms including intron splicing and polycistronic gene expression system.

Supplementary Figures

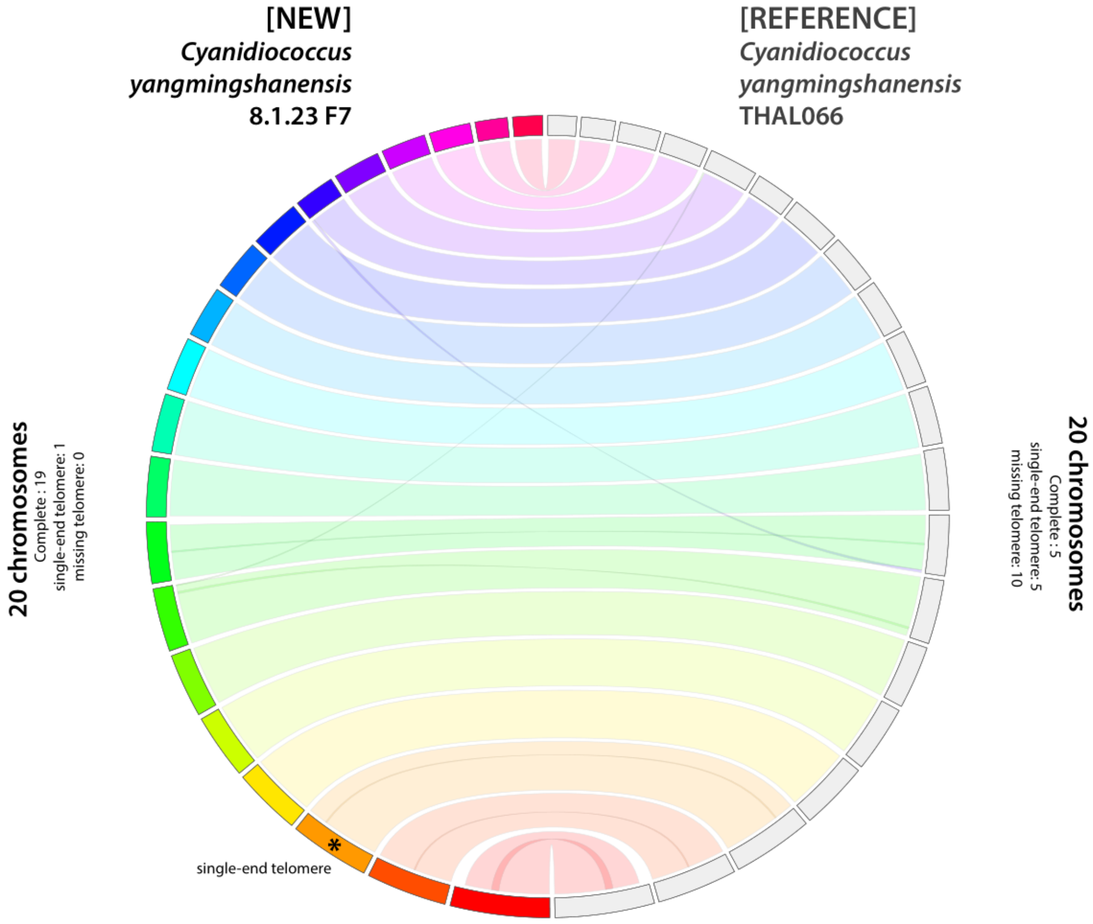

Supplementary Fig. 1. A chromosome comparison of two *Cyanidiococcus yangmingshanensis* strains. The genomes of newly sequenced 8.1.23 F7 and publicly available THAL066 strains were used for comparison based on nucleotide similarity.

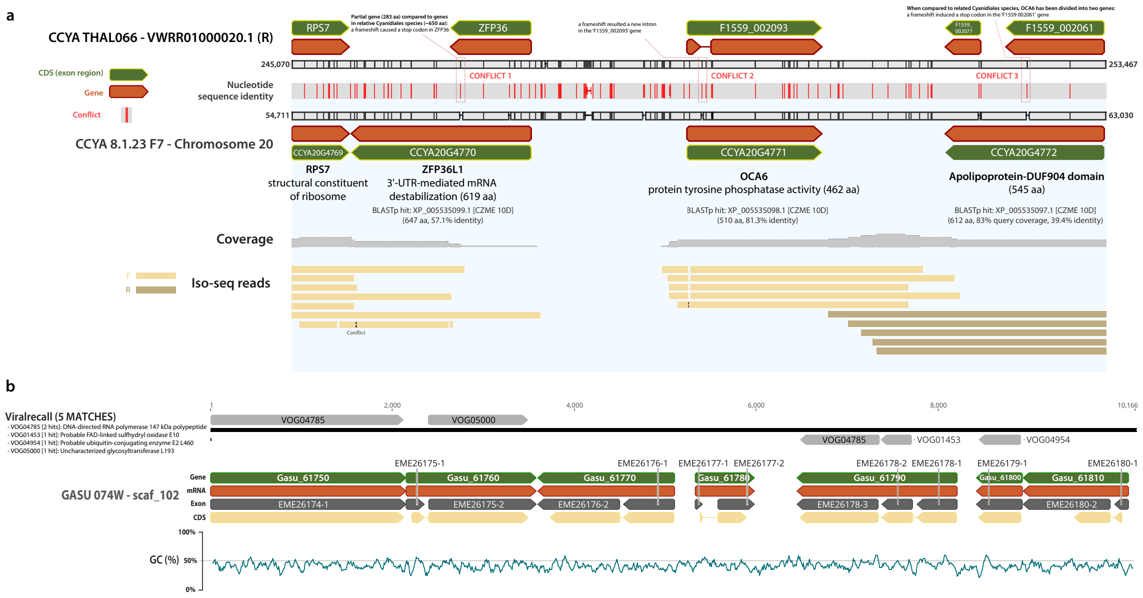

Supplementary Fig. 2. Issues with reported genomes and gene modeling in Cyanidiophyceae. a A possible misidentification of gene modeling in the THAL066 genome. Annotation comparison in a conserved region of two *Cyanidiococcus* strains. F: forward, R: reverse. **b** Viral contamination candidate found in *Galdieria sulphuraria* 074W genome.

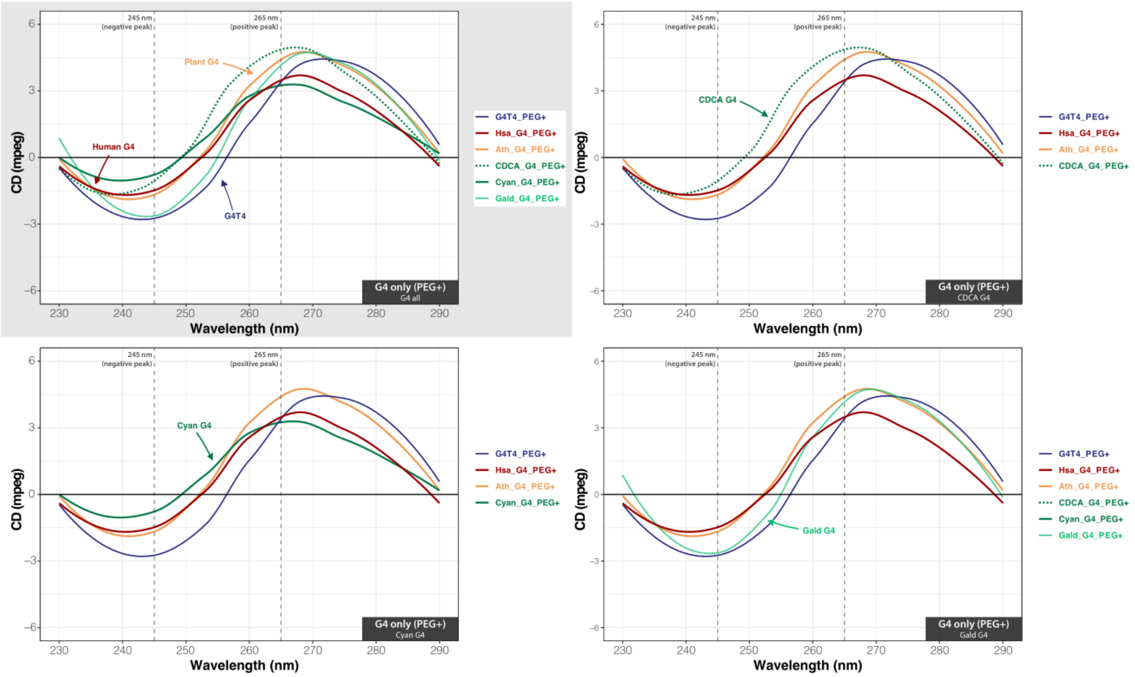

Supplementary Fig. 3. CD spectra of G-rich strand oligonucleotides. ‘G4T4’, ‘Hsa_G4’, and ‘Ath_G4’ were used as positive controls for the formation of G-quadruplexes.

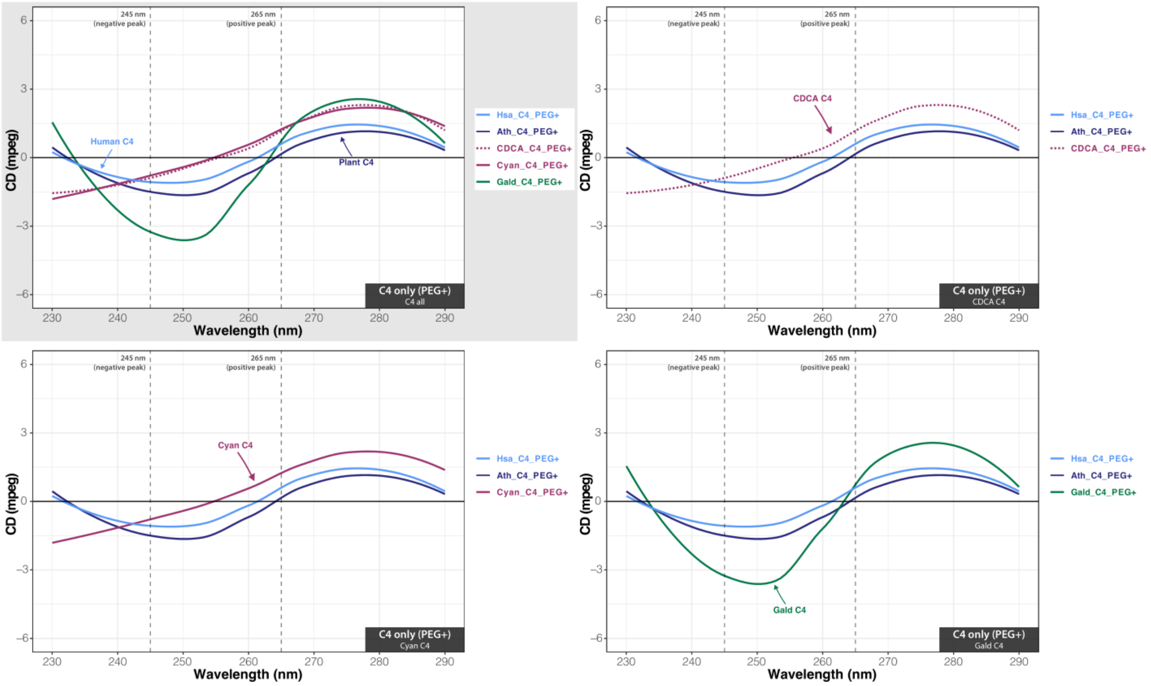

Supplementary Fig. 4. CD spectra of C-rich strand oligonucleotides. 'Hsa_C4' and 'Ath_C4' were used as C-rich strand controls.

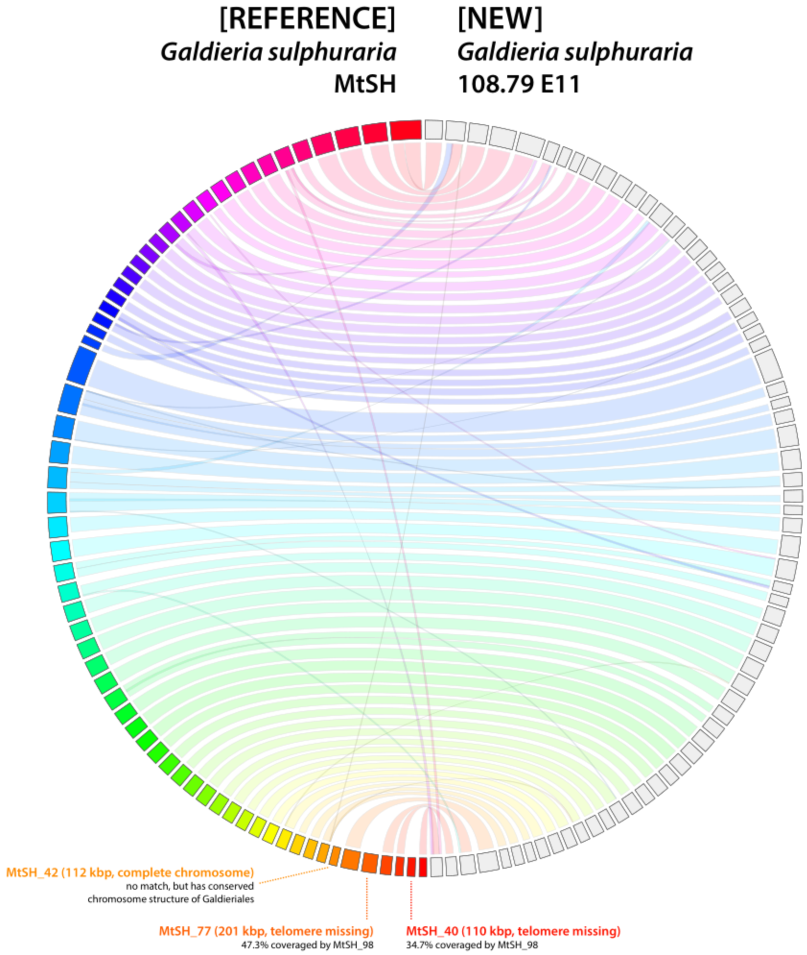

Supplementary Fig. 5. A chromosome comparison of two *Galdieria sulphuraria* strains. The genomes of newly sequenced 108.79 E5 and publicly available MtSh strains were used for comparison based on nucleotide similarity.

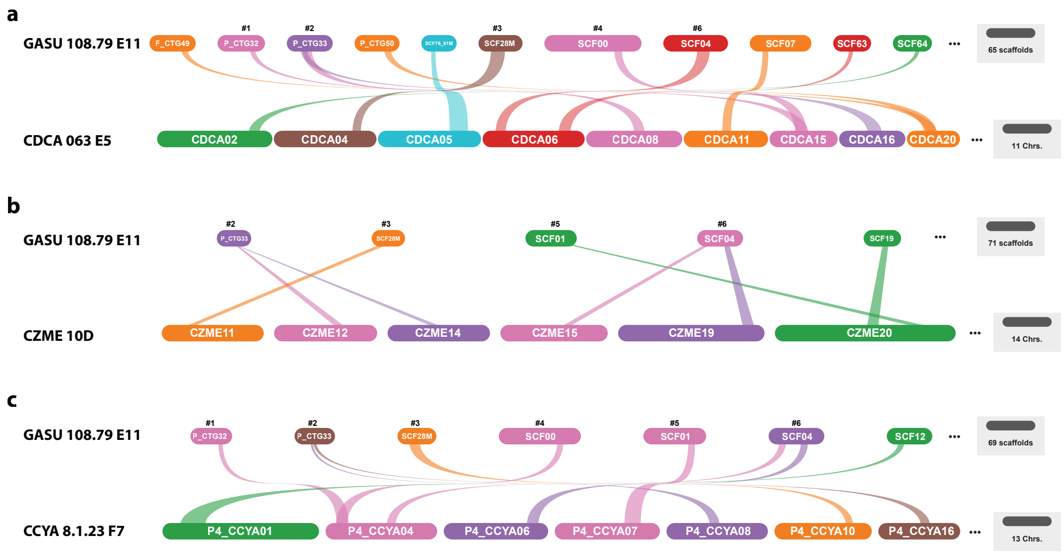

Supplementary Fig. 6. A chromosome comparison between *Galdieria sulphuraria* 108.79 E11 and other Cyanidiales genomes based on gene syntenies. a Chromosomal comparison with *Cyanidium* genome, **b** Chromosomal comparison with *Cyanidioschyzon* genome, **c** Chromosomal comparison with *Cyanidiococcus* genome. Unaligned chromosomes were placed in the right side (dark grey color).

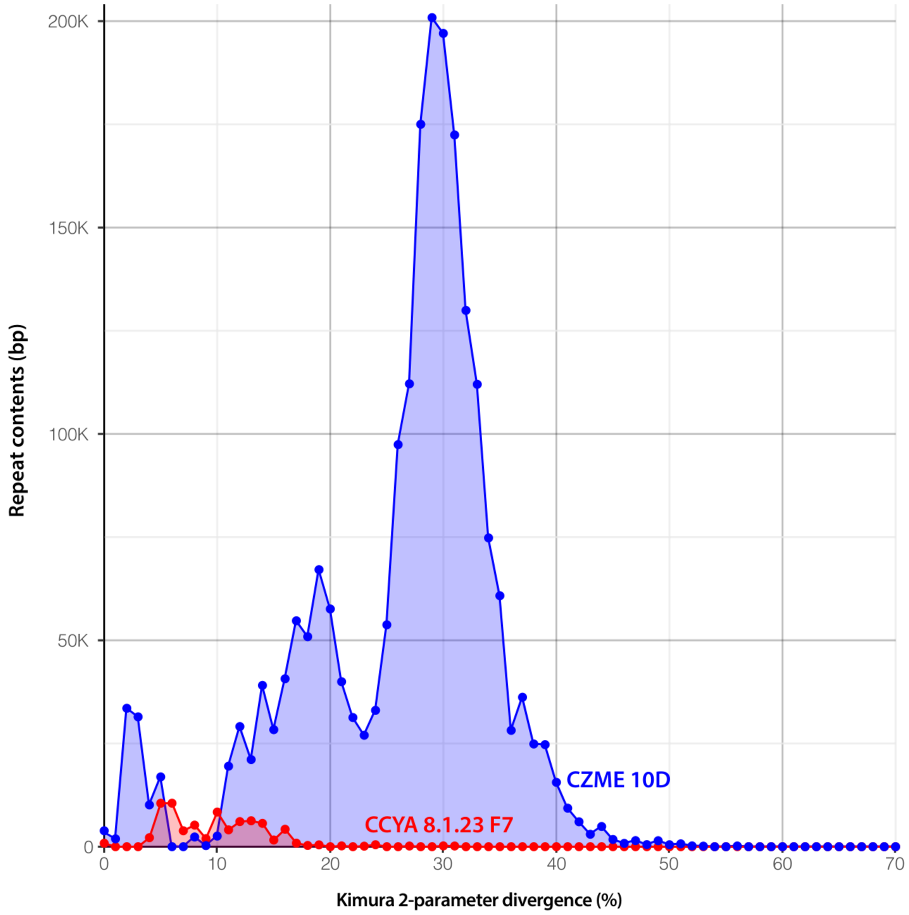

Supplementary Fig. 7. Long terminal repeat (LTR) composition and divergence rate of *Cyanidiococcus* (CCYA 8.1.23 F7; red color) and *Cyanidioschyzon* (CZME 10D; blue color).

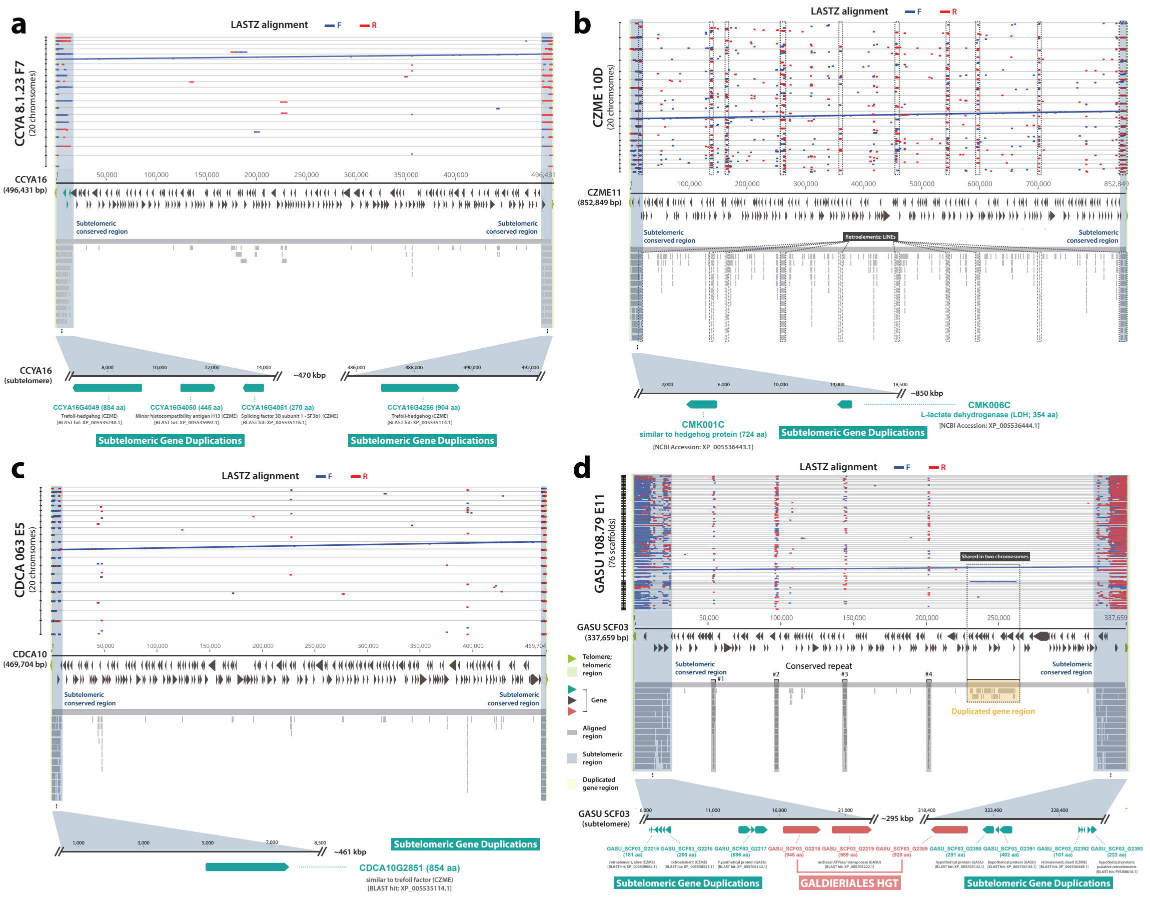

Supplementary Fig. 8. A chromosome structure of four cyanidiophycean species. a *Cyanidiococcus* (CCYA 8.1.23 F7), **b** *Cyanidioschyzon* (CZME 10D), **c** *Cyanidium* (CDCA 063 E5), and **d** *Galdieria* (GASU 108.79 E11). Subtelomeric duplicated genes are indicated by the turquoise color arrow, and putative HGTs are indicated by the red color arrow. In LASTZ alignments, the forward strand (F) is colored blue and the reverse strand (R) is colored red.

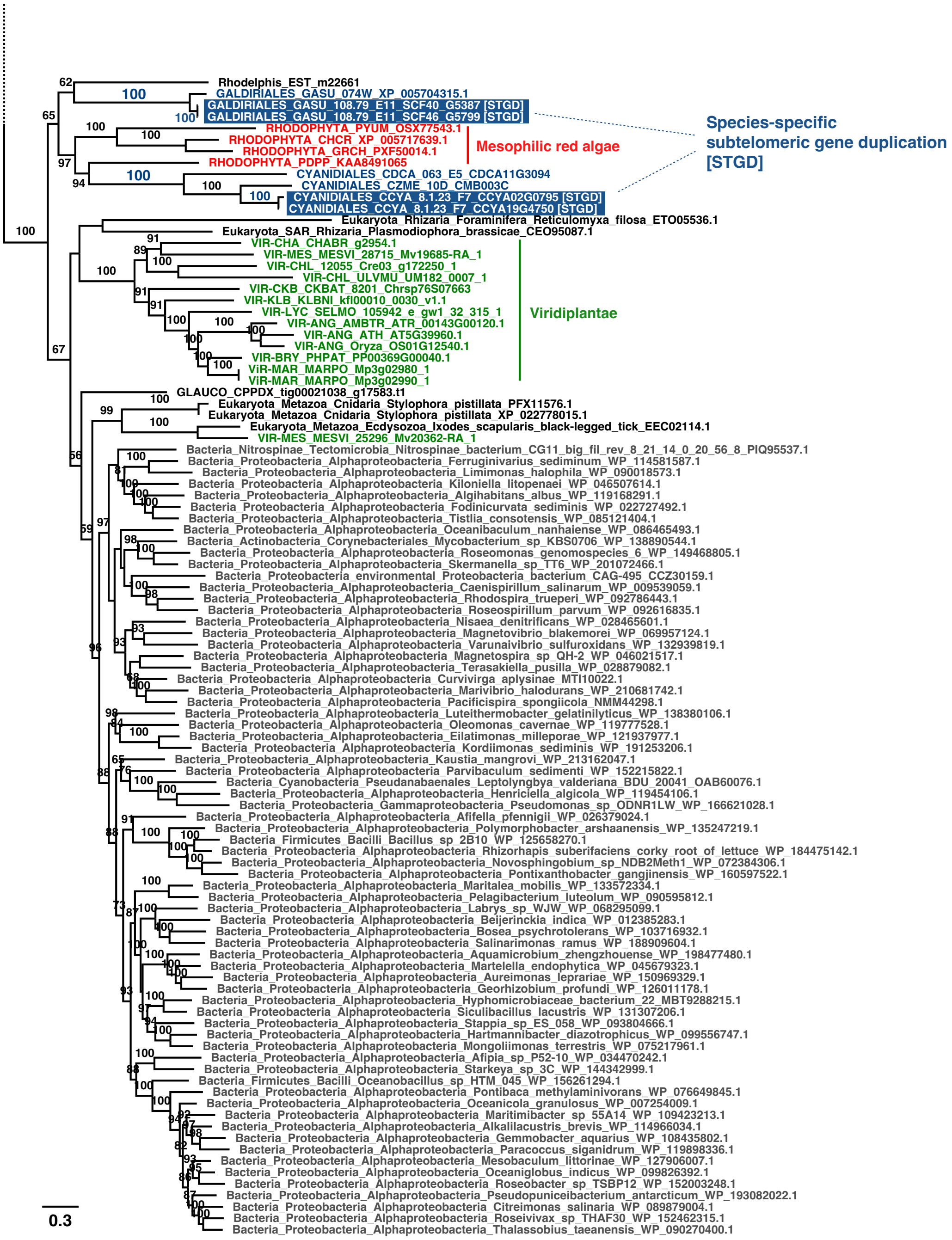

Supplementary Fig. 9. Independent subtelomeric gene duplications (STGDs) of GTP-binding proteins in Cyanidiophyceae. For readability, only a subtelomeric duplicated gene clade was visualized, and the full phylogenetic tree can be found in the Dryad database.

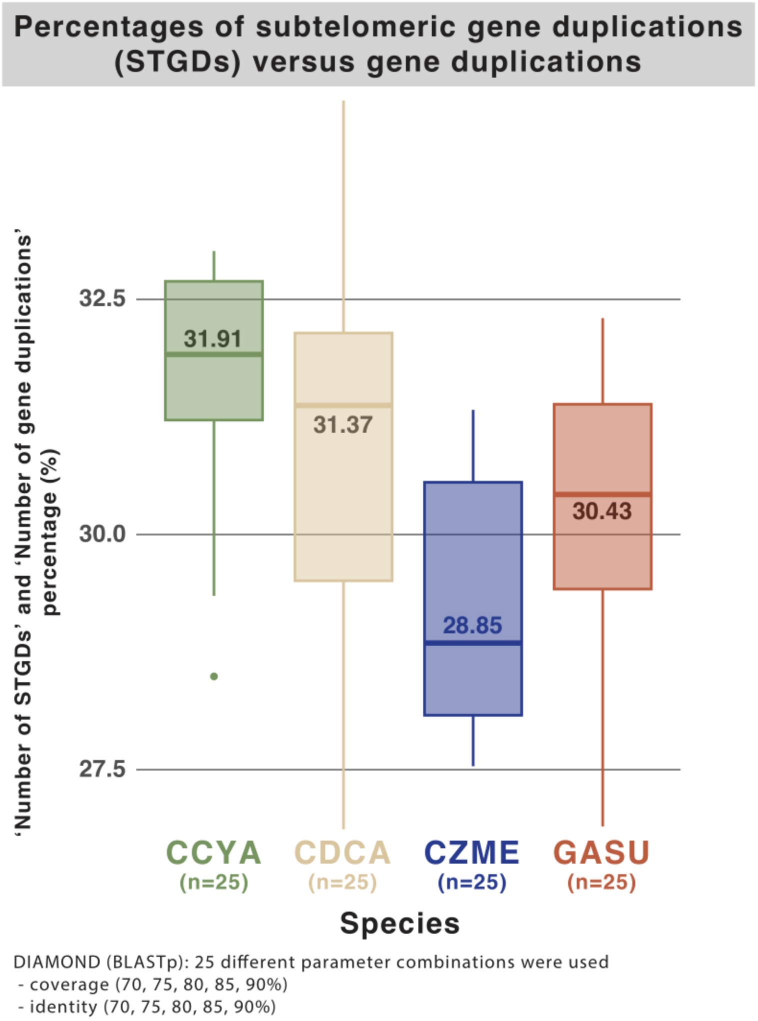

Supplementary Fig. 10. The proportion of subtelomeric gene duplication to overall gene duplication in four cyanidiophycean species. 25 combinations of query & subject coverage and protein identity parameters were considered for DIAMOND protein-protein searches**.** Box plots (n=25) are used to show the data range, highlighting the 25th (Q1) to 75th (Q3) percentiles of data with the mean value in a thick line. Extended lines from the box (whisker) indicate 10th percentile in the lower and the 90th percentile in the upper. CDCA: *Cyanidium*, CCYA: *Cyanidiococcus*, CZME: *Cyanidioschyzon*, GASU: *Galdieria*.

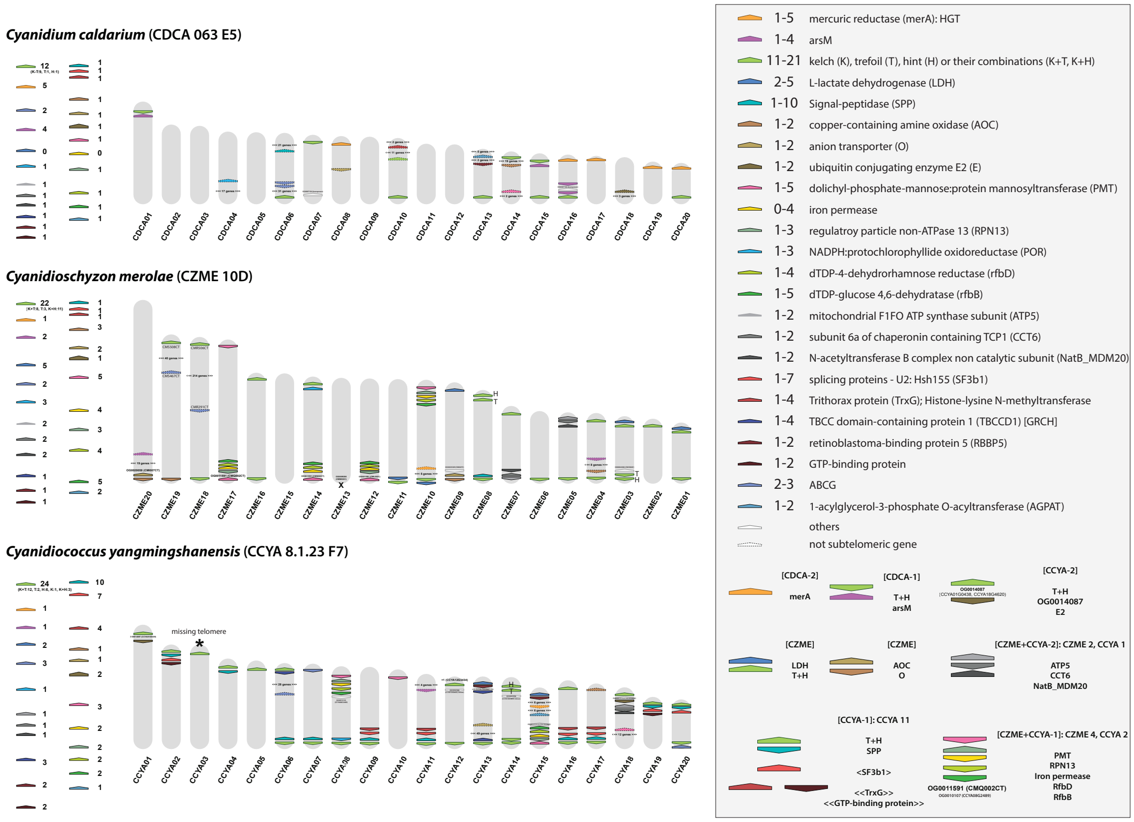

Supplementary Fig. 11. Chromosome map of subtelomeric duplicated genes from three Cyanidiales species.

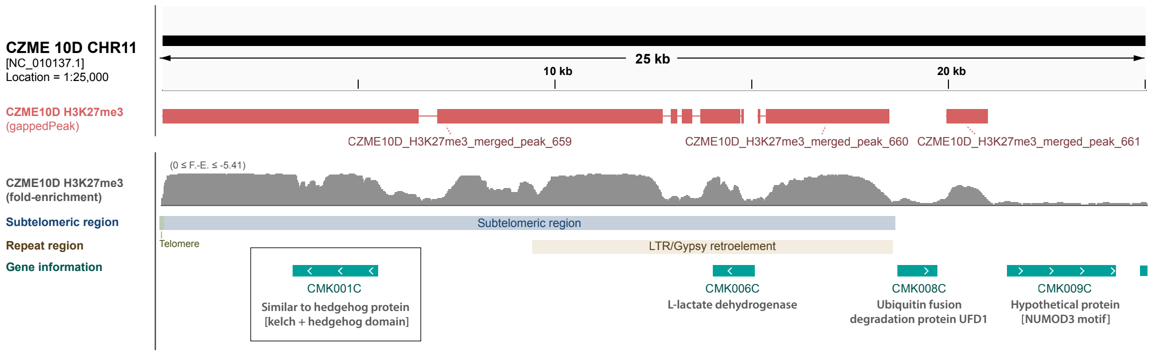

Supplementary Fig. 12. Identification of H3K27me3 modification in subtelomere of *Cyanidioschyzon merolae* 10D (chromosome 11). Below the histone modification peaks, genomic features were mapped and annotated.

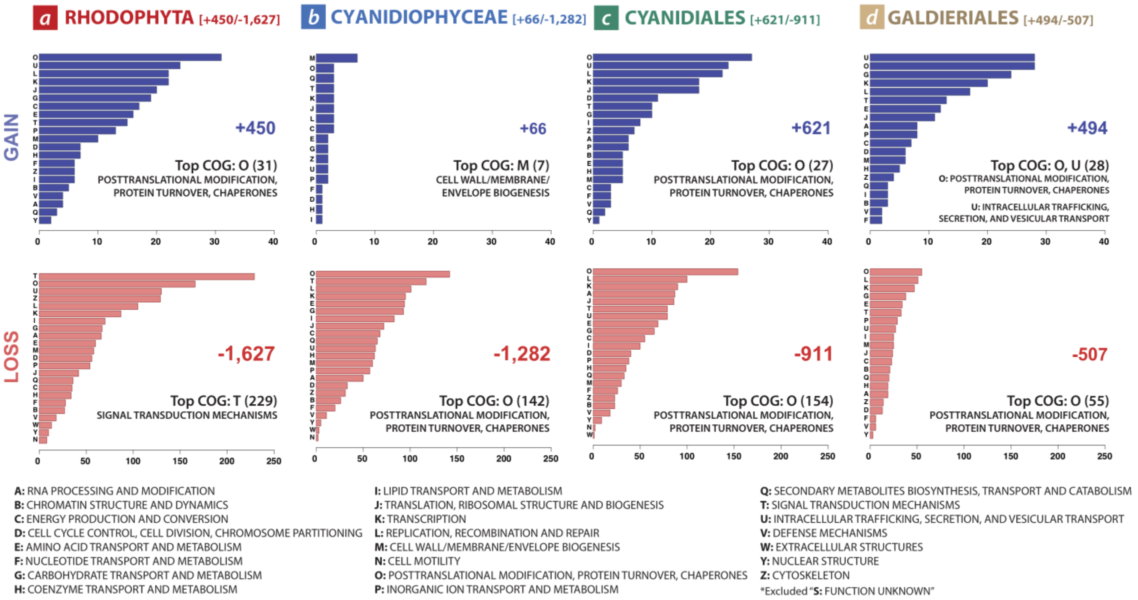

Supplementary Fig. 13. COGs of gain and loss events from Dollo parsimony result from Figure 3. The Top COGs were annotated in each COG gain and loss plot. **a** divergence of red algae, **b** divergence of Cyanidiophyceae, **c** divergence of Cyanidiales, **d** divergence of Galdieriales.

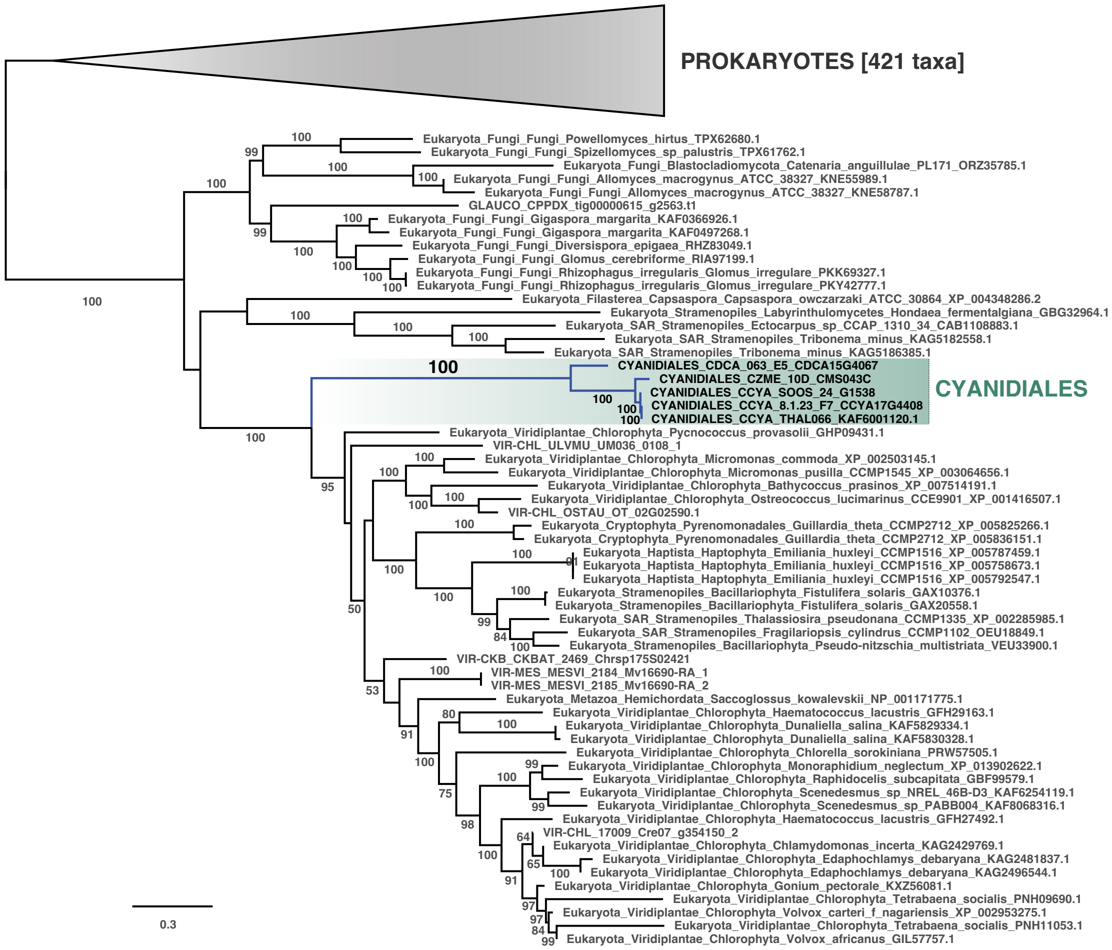

Supplementary Fig. 14. A phylogeny of *ars*J gene. The *ars*J genes in Cyanidiales are found in other eukaryotic algae, implying that HGT from prokaryotic species is unlikely.

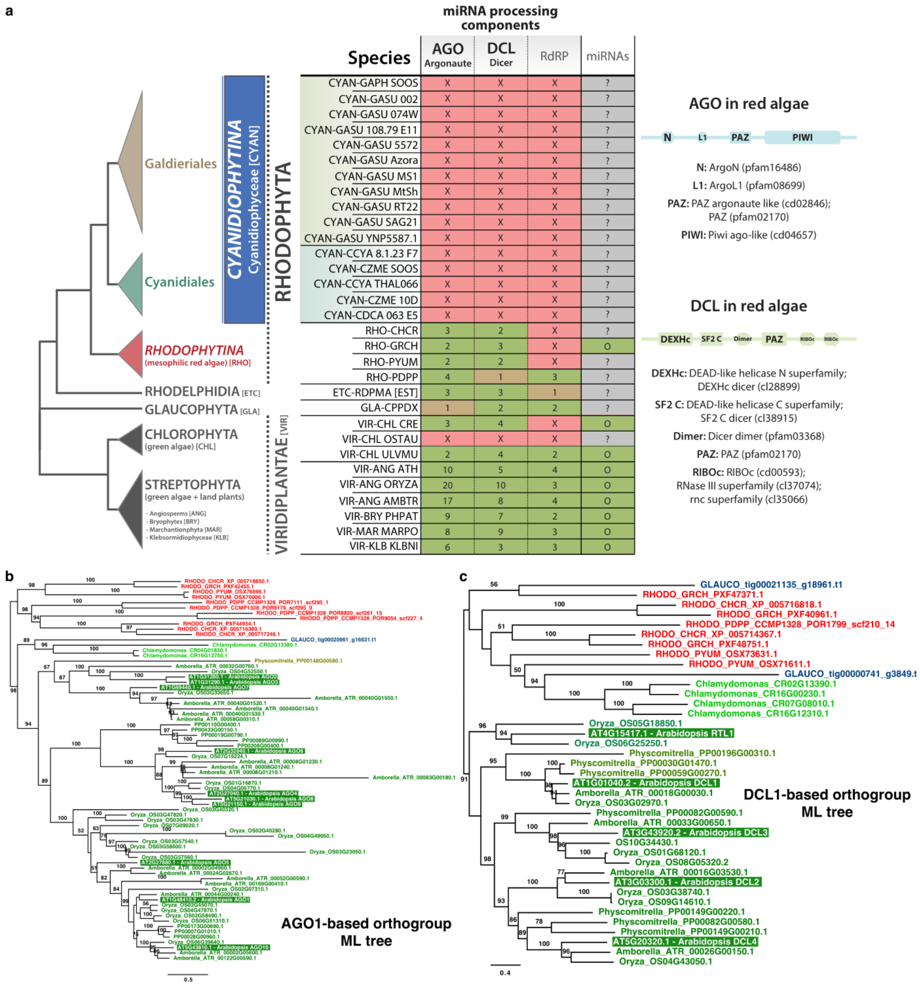

Supplementary Fig. 15. Evidence for miRNA system loss in Cyanidiophyceae. a The distribution of conserved miRNA processing proteins was studied in representative Archaeplastida. The domain structure of DCL and AGO in red algae was also analyzed. Cyanidiophyceae species lack all core components of the miRNA processing pathway. The absence of components is indicated by the red color, a single copy of genes is indicated by the brown color, and more than two copies of genes (or presence of miRNA) are indicated by the green color. The grey color indicates that there are no references. **b** A phylogeny of AGO genes using representative species in Archaeplastida. **c** A phylogeny of DCL genes using representative species in Archaeplastida.

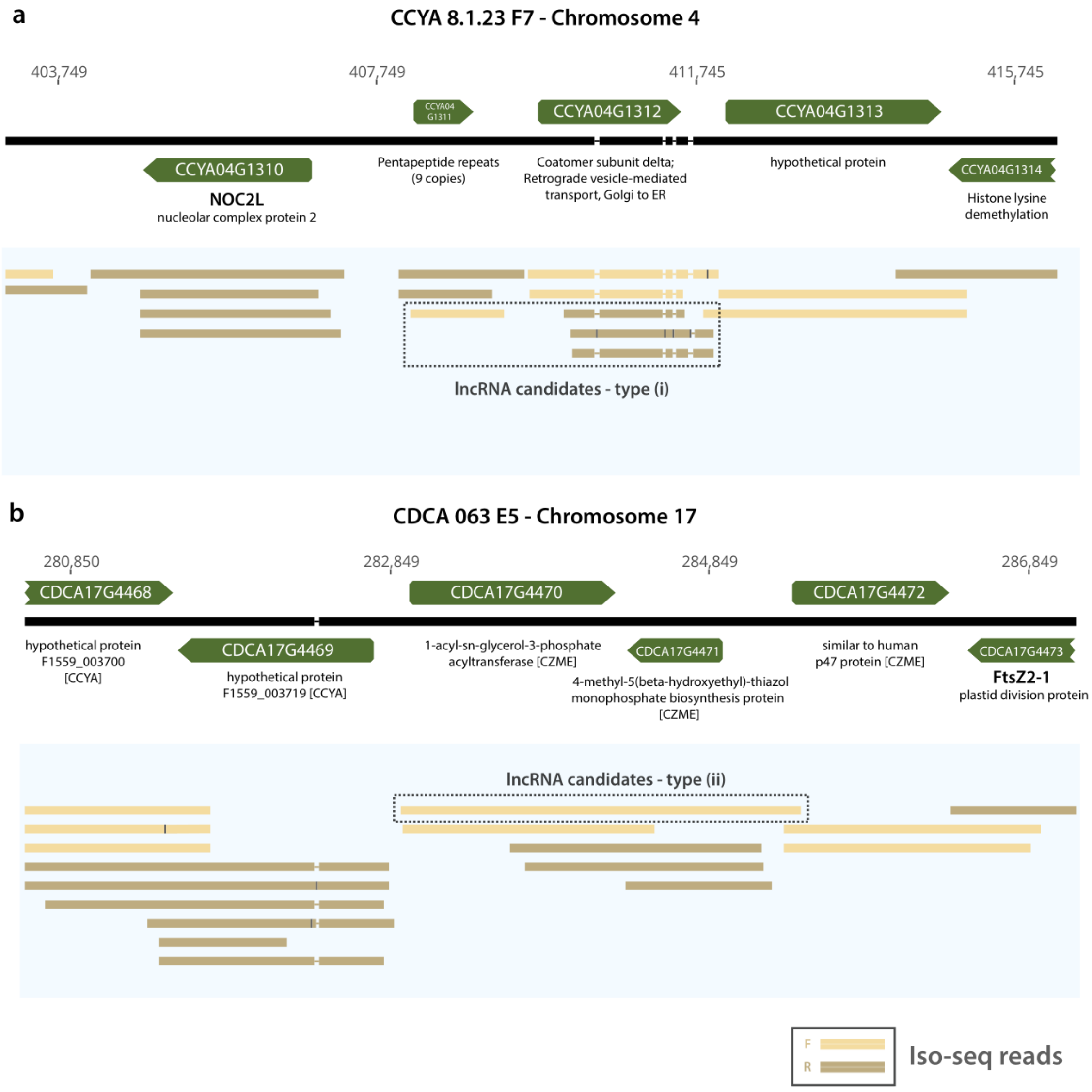

Supplementary Fig. 16. Long noncoding RNA (lncRNA) candidate in Cyanidiales genomes. **a** lncRNA candidates from *Cyanidiococcus* (CCYA 8.1.23 F7) chromosome 4, **b** lncRNA candidates from *Cyanidium* (CDCA 063 E5) chromosome 17. F: forward, R: reverse.

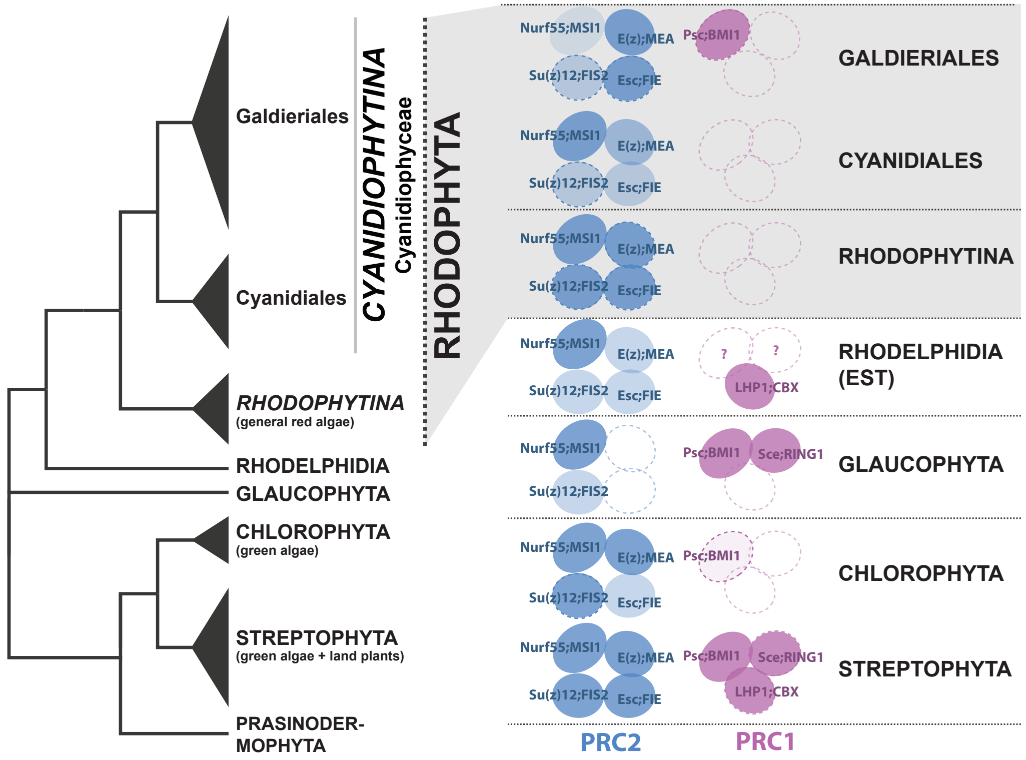

		GALDIER IALES	CYANID IALES	RED ALGAE	RHODEL PHIDIA	GLAUCO PHYTA	CHLORO PHYTA	STREPTO PHYTA
PRC2	Core PRC2 - Nurf55;MSI	1	2	2-3	3	2	2	2-5
	Core PRC2 - E(z);MEA	1-3	1	0-6	1	0	1	1-7
	Core PRC2 - Su(z)12;FIS2	0-1	0-1	0-2	1	0	0-2	1-4
	Core PRC2 - Esc;FIE	0-3	1	0-2	1	1	1	1-2
PRC1	Core PRC1 - BMI1	0-3	0	0	0	2	0-1	1-6
	Core PRC1 - RING1	0	0	0	0	3	0	0-6
	Core PRC1 - LHP1	0	0	0	2	0	0	0-4

Supplementary Fig. 17. Genomic survey of polycomb groups in Archaeplastida. PRC1: polycomb repressive complex 1, PRC2: polycomb repressive complex 2. The absence of genes is indicated by the red color, a single copy of genes is indicated by the brown color, and more than two copies of genes are indicated by the green color.

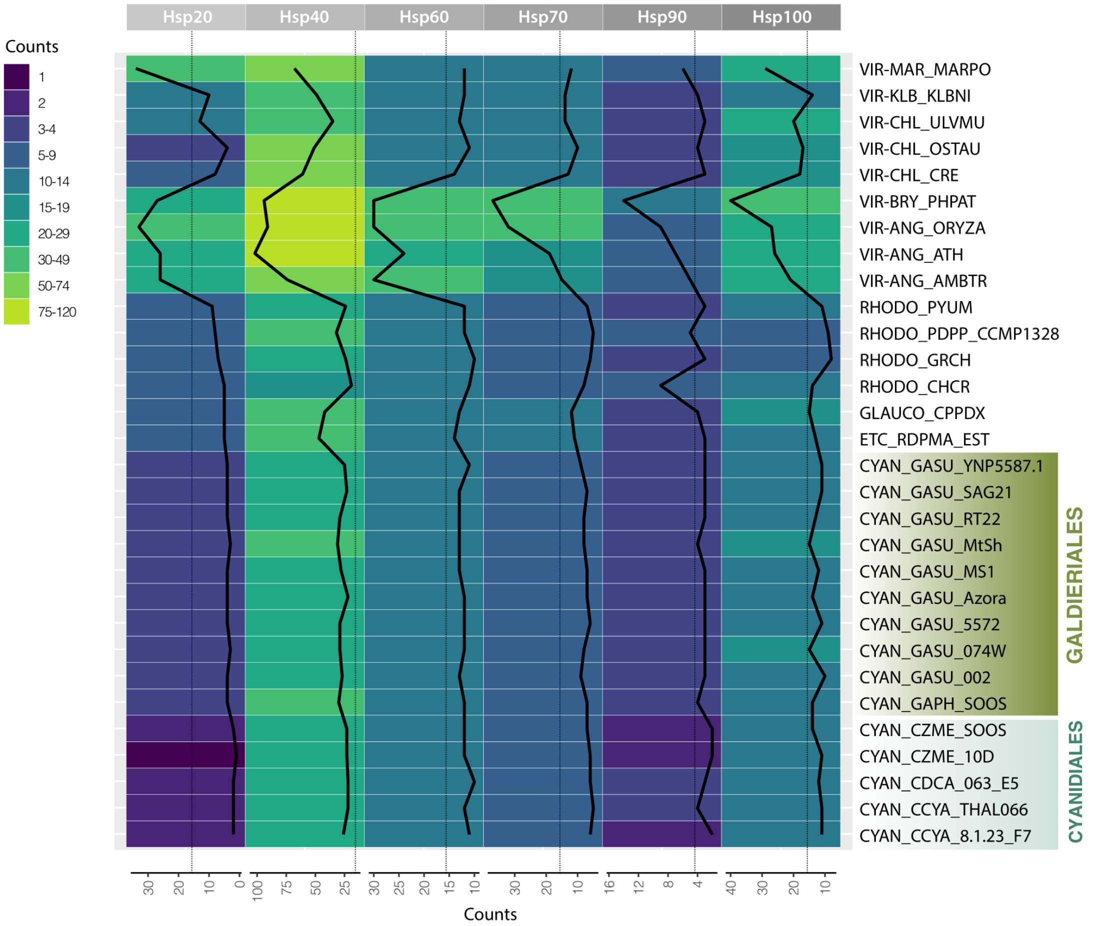

Supplementary Fig. 18. Chaperon profiles of selected Archaeplastida taxa. A heatmap depicts the counts of different types of chaperone genes from 30 Archaeplastida species.

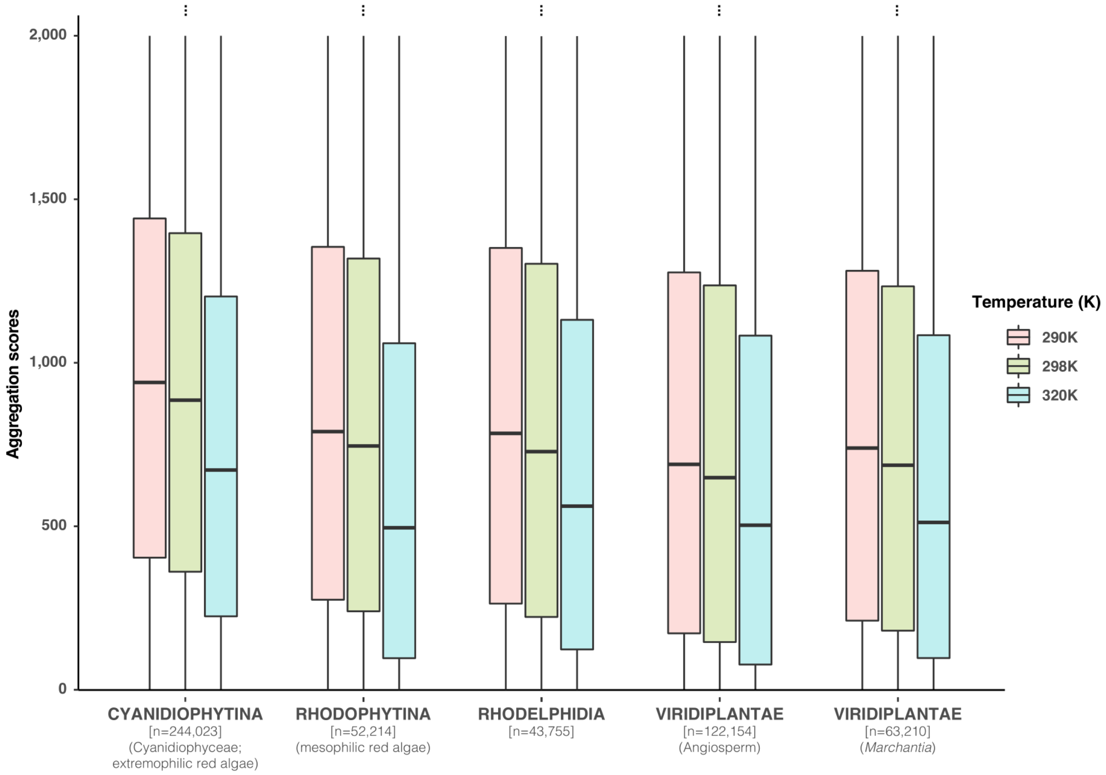

Supplementary Fig. 19. Estimated value of aggregation-prone regions using proteomes of Archaeplastida. Aggregation scores were estimated in three different temperatures (290K, 298K, 320K). Box plots are used to show the data range, highlighting the 25th (Q1) to 75th (Q3) percentiles of data with the mean value in a thick line.

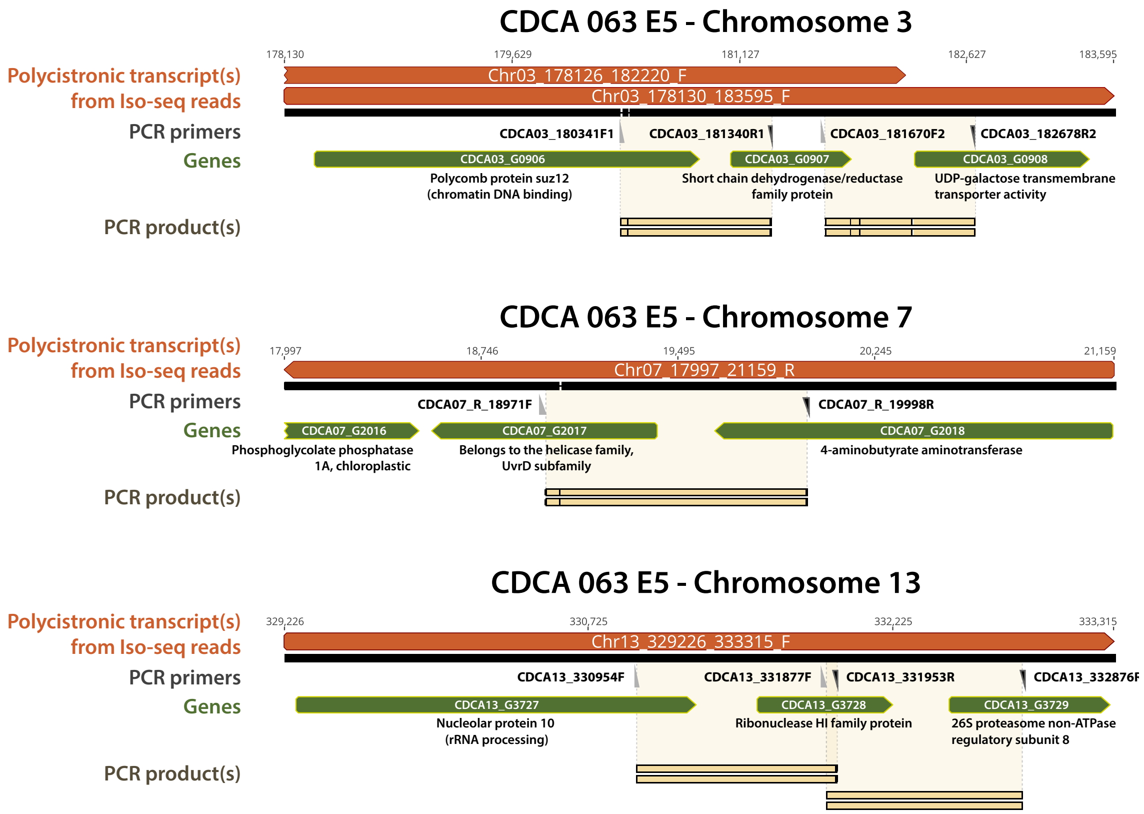

Supplementary Fig. 20. Polycistronic gene expression in *Cyanidium* (CDCA 063 E5).

Randomly selected polycistronic candidates were verified by PacBio Iso-Seq data and manually confirmed by reverse transcription-PCR.

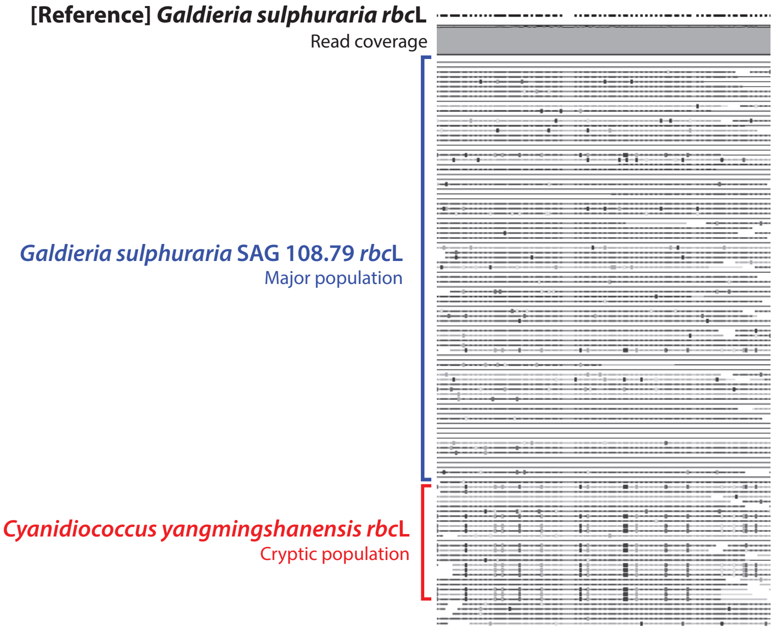

Supplementary Fig. 21. Verification of cryptic population using previous sequencing data. Illumina WGS reads were used check cryptic population. Illumina WGS reads were used to examine the cryptic population. Each row represents reads from Illumina data, and the highlighted portion within reads indicates a residue conflict when compared to the reference sequence. Two or more mapping patterns will appear if there are any existing cryptic cyanidiophycean species.

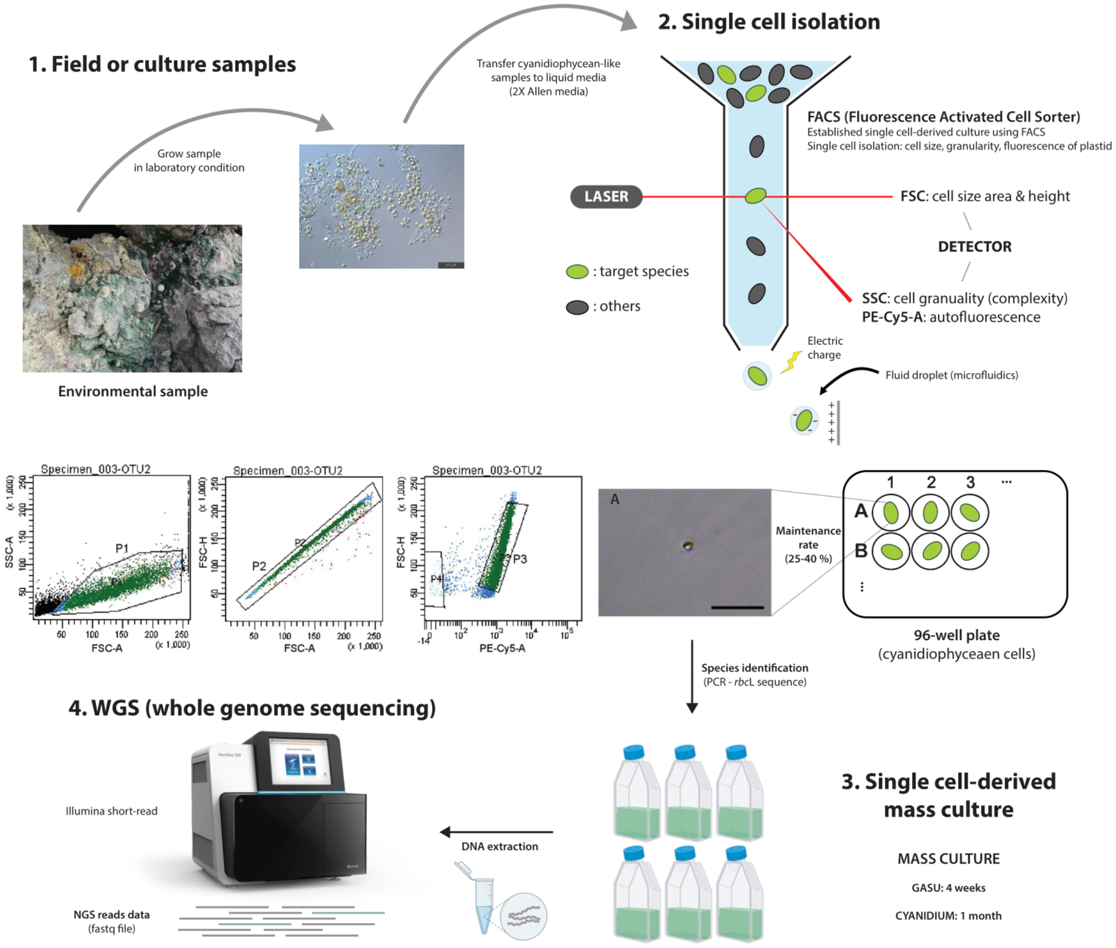

Supplementary Fig. 22. The overview of establishing cyanidiophycean unialgal culture using single cell sorting methods. The image was created with BioRender.com.

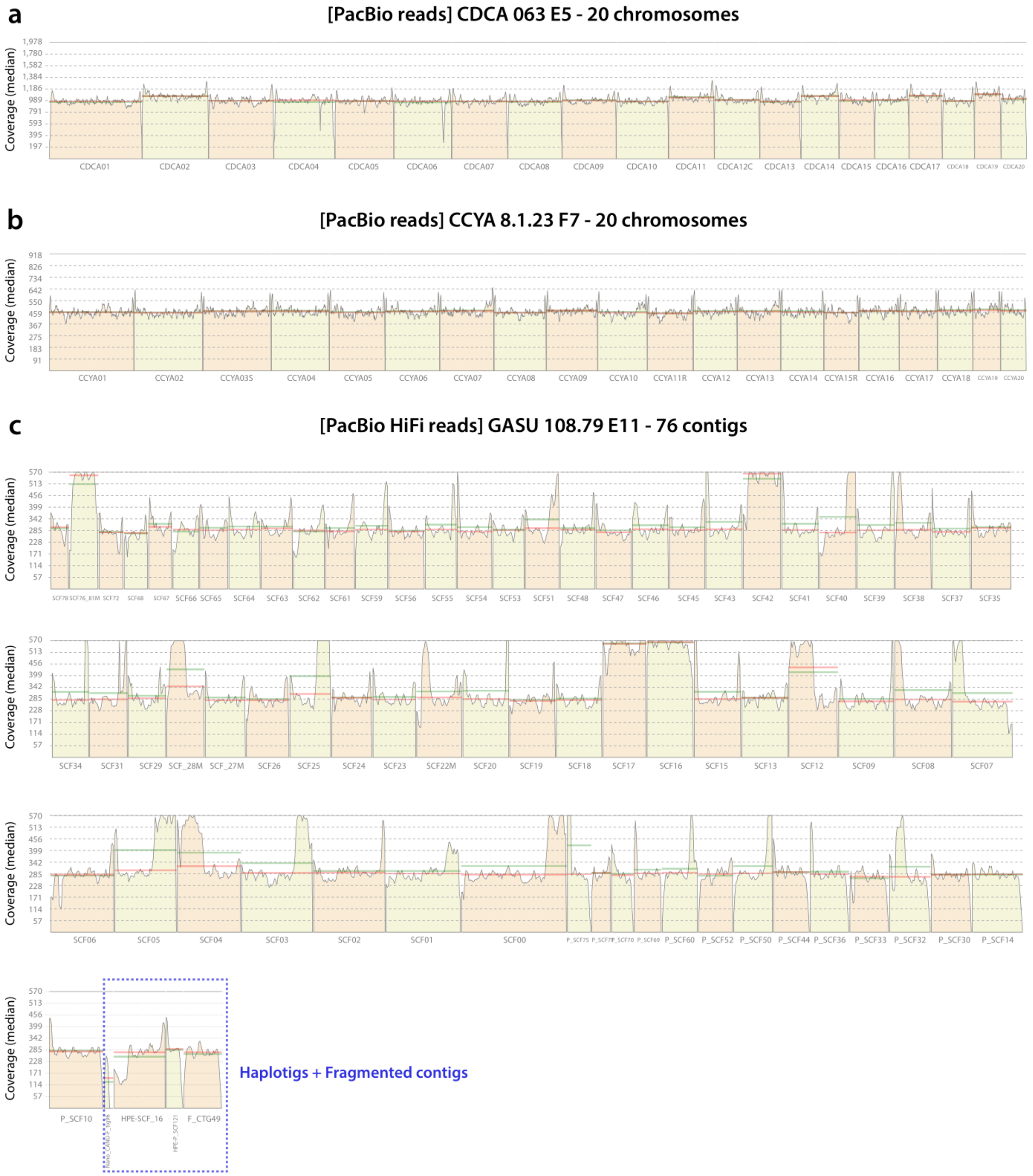

Supplementary Fig. 23. Mapping coverage of established cyanidiophycean genomes. For sequencing coverage verification, PacBio long-reads were used. **a** *Cyanidium* (CDCA 063 E5), **b** *Cyanidiococcus* (CCYA 8.1.23 F7), **c** *Galdieria* (GASU 108.79 E11).

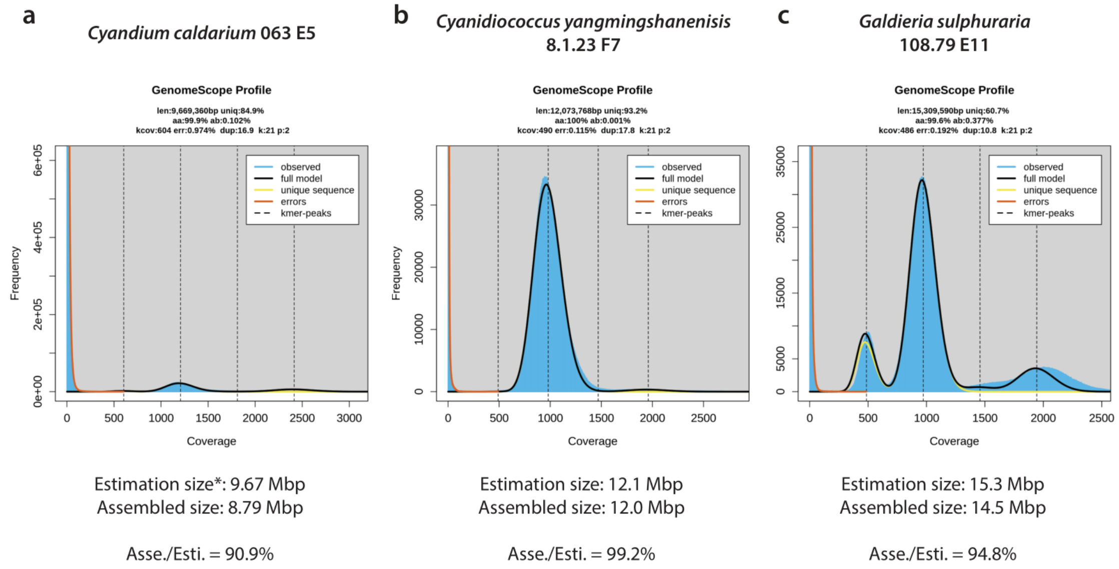

Supplementary Fig. 24. Genome size estimation of three cyanidiophycean species. The genome size of each strain was estimated using the *k*-mer base method and Illumina WGS reads. Due to estimation error using organelle-removed reads, the estimated size of *Cyanidium* (marked with an asterisk) was predicted with raw data. **a** *Cyanidium* (CDCA 063 E5), **b** *Cyanidiococcus* (CCYA 8.1.23 F7), **c** *Galdieria* (GASU 108.79 E11).

Supplementary References

1 Richards, E. J. & Ausubel, F. M. Isolation of a higher eukaryotic telomere from *Arabidopsis thaliana*. *Cell* **53**, 127-136 (1988).

2 Frydrychova, R. C. & Mason, J. M. Telomeres: their structure and maintenance. *The Mechanisms of DNA Replication. David Stuart (Ed.). InTech Publishing, London, UK*, 423-443 (2013).

3 Fulnečková, J. *et al.* A broad phylogenetic survey unveils the diversity and evolution of telomeres in eukaryotes. *Genome Biol. Evol.* **5**, 468-483 (2013).

4 Paeschke, K., Simonsson, T., Postberg, J., Rhodes, D. & Lipps, H. J. Telomere end-binding proteins control the formation of G-quadruplex DNA structures in vivo. *Nat. Struct. Mol. Biol.* **12**, 847-854 (2005).

5 Kan, Z.-y. *et al.* G-quadruplex formation in human telomeric (TTAGGG) 4 sequence with complementary strand in close vicinity under molecularly crowded condition. *Nucleic Acids Res.* **35**, 3646-3653 (2007).

6 Maruyama, S. *et al.* Centromere structures highlighted by the 100%-complete *Cyanidioschyzon merolae* Genome. *Plant Signal. Behav.* **3**, 140-141 (2008).

7 Kanesaki, Y., Imamura, S., Matsuzaki, M. & Tanaka, K. Identification of centromere regions in chromosomes of a unicellular red alga, *Cyanidioschyzon merolae*. *FEBS Lett.* **589**, 1219-1224 (2015).

8 Rossoni, A. *et al.* The genomes of polyextremophilic Cyanidiales contain 1% horizontally transferred genes with diverse adaptive functions. *eLife* **8** (2019).

9 Moreira, D., López-Archilla, A.-I., Amils, R. & Marín, I. Characterization of two new thermoacidophilic microalgae: Genome organization and comparison with *Galdieria sulphuraria*. *FEMS Microbiol. Lett.* **122**, 109-114 (1994).

10 Swindell, W. R., Huebner, M. & Weber, A. P. Transcriptional profiling of Arabidopsis heat shock proteins and transcription factors reveals extensive overlap between heat and non-heat stress response pathways. *BMC Genomics* **8**, 125 (2007).

11 Shabalina, S. A. & Koonin, E. V. Origins and evolution of eukaryotic RNA interference. *Trends Ecol. Evol.* **23**, 578-587 (2008).

12 Huang, A., Wu, X., Wang, G., Jia, Z. & He, L. Computational prediction of microRNAs and their targets from three unicellular algae species with complete genome sequences. *Can. J. Microbiol.* **57**, 1052-1061 (2011).

13 Gonzalez-Garay, M. L. in *Transcriptomics and Gene Regulation* (ed Jiaqian Wu) 141-160 (Springer Netherlands, 2016).

14 Li, H. *et al.* Genome-wide long non-coding RNA screening, identification and characterization in a model microorganism *Chlamydomonas reinhardtii*. *Sci. Rep.* **6**, 34109 (2016).

15 Cormier, A. *et al.* Re-annotation, improved large-scale assembly and establishment of a catalogue of noncoding loci for the genome of the model brown alga *Ectocarpus*. *New Phytol.* **214**, 219-232 (2017).

16 Bantignies, F. & Cavalli, G. Polycomb group proteins: repression in 3D. *Trends Genet.* **27**, 454-464 (2011).

17 Hennig, L. & Derkacheva, M. Diversity of Polycomb group complexes in plants: same rules, different players? *Trends Genet.* **25**, 414-423 (2009).

18 Bourdareau, S. *et al.* Histone modifications during the life cycle of the brown alga *Ectocarpus*. *Genome Biol.* **22**, 12 (2021).

19 Mikulski, P., Komarynets, O., Fachinelli, F., Weber, A. P. M. & Schubert, D. Characterization of the polycomb-group mark H3K27me3 in unicellular algae. *Front. Plant Sci.* **8** (2017).

20 Laksanalamai, P. & Robb, F. T. Small heat shock proteins from extremophiles: a review. *Extremophiles* **8**, 1-11 (2004).

21 Qiu, X.-B., Shao, Y.-M., Miao, S. & Wang, L. The diversity of the DnaJ/Hsp40 family, the crucial partners for Hsp70 chaperones. *Cell. Mol. Life Sci.* **63**, 2560-2570 (2006).

22 Malinovska, L., Palm, S., Gibson, K., Verbavatz, J.-M. & Alberti, S. *Dictyostelium discoideum* has a highly Q/N-rich proteome and shows an unusual resilience to protein aggregation. *Proc. Natl. Acad. Sci. USA* **112**, E2620-E2629 (2015).

23 Draceni, Y. & Pechmann, S. Pervasive convergent evolution and extreme phenotypes define chaperone requirements of protein homeostasis. *Proc. Natl. Acad. Sci. USA* **116**, 20009-20014 (2019).

24 Cho, C. H. *et al.* Potential causes and consequences of rapid mitochondrial genome evolution in thermoacidophilic *Galdieria* (Rhodophyta). *BMC Evol. Biol.* **20**, 112 (2020).

25 Petroll, R. *et al.* Signatures of transcription factor evolution and the secondary gain of red algae complexity. *Genes* **12**, 1055 (2021).

26 Kim, B. H. & Schöffl, F. Interaction between Arabidopsis heat shock transcription factor 1 and 70 kDa heat shock proteins. *J. Exp. Bot.* **53**, 371-375 (2002).

27 von Koskull-Döring, P., Scharf, K.-D. & Nover, L. The diversity of plant heat stress transcription factors. *Trends Plant Sci.* **12**, 452-457 (2007).

28 Nishizawa, A. *et al.* Arabidopsis heat shock transcription factor A2 as a key regulator in response to several types of environmental stress. *Plant J.* **48**, 535-547 (2006).

29 Shim, D. *et al.* Orthologs of the class A4 heat shock transcription factor HsfA4a confer cadmium tolerance in wheat and rice *Plant Cell* **21**, 4031-4043 (2009).

30 Blumenthal, T. Gene clusters and polycistronic transcription in eukaryotes. *Bioessays* **20**, 480-487 (1998).

31 Gordon, S. P. *et al.* Widespread polycistronic transcripts in fungi revealed by single-molecule mRNA sequencing. *PLoS One* **10**, e0132628 (2015).

32 Wang, K. *et al.* Multi-strategic RNA-seq analysis reveals a high-resolution transcriptional landscape in cotton. *Nat. Commun.* **10**, 1-15 (2019).

33 Gallaher, S. D. *et al.* Widespread polycistronic gene expression in green algae. *Proc. Natl. Acad. Sci. USA* **118**, e2017714118 (2021).

34 Luke, G. A. *et al.* Occurrence, function and evolutionary origins of '2A-like' sequences in virus genomes. *J. Gen. Virol.* **89**, 1036-1042 (2008).
