# Supplementary material for: Genome-wide signatures of adaptation to extreme environments in red algae
Source: Nat Commun. 2023 Jan 4;14:10. doi: 10.1038/s41467-022-35566-x (PMC9812998; doi:10.1038/s41467-022-35566-x)
Supplement: Supplementary file 3 — Description of Additional Supplementary Files [file 41467_2022_35566_MOESM3_ESM.pdf]

Description of Additional Supplementary Files

File Name: Supplementary Data 1
Description: **Sequencing information of Cyanidiophyceae used in this study.** CCYA: *Cyanidiococcus yangmingshanensis*, CDCA: *Cyanidium caldarium*, GASU: *Galdieria sulphuraria*.

File Name: Supplementary Data 2
Description: **Oligonucleotides designed for G-quadruplex verification.**

File Name: Supplementary Data 3
Description: **Genomic characteristics of cyanidiophycean genomes and reference red algal genomes.** Single-end (SE) telomere scaffolds with size greater than 10 kbp was counted.

File Name: Supplementary Data 4
Description: **Repeat comparison of *Cyanidioschyzon merolae* 10D and *Cyanidiococcus yangmingshanensis* 8.1.23 F7.**

File Name: Supplementary Data 5
Description: **Subtelomere identification and gene contents in the Cyanidiales genomes (*Cyanidium*, *Cyanidiococcus*, *Cyanidioschyzon*).**

File Name: Supplementary Data 6
Description: **Subtelomere identification and gene contents in the *Galdieria sulphuraria* 108.79 E11 genome.**

File Name: Supplementary Data 7
Description: **List of subtelomeric duplicated orthogroups in three Cyanidiophyceae species.**

File Name: Supplementary Data 8
Description: **Ka/Ks analysis of mercuric reductase genes in Cyanidiophyceae.** A one-sided test in a Fisher's Exact Test was used to calculate P-values.

File Name: Supplementary Data 9
Description: **Genome and transcriptome information used in this study.**

File Name: Supplementary Data 10
Description: **miRNA prediction of four cyanidiophycean species predicted by rfam v14.5.**

File Name: Supplementary Data 11
Description: **Oligonucleotide primers designed for polycistronic transcript candidates.**

File Name: Supplementary Data 12
Description: **Components of modified 5X Allen medium [pH 1.5].**

File Name: Supplementary Data 13
Description: **The proportion of subtelomeric gene duplication (STGD) to overall gene duplication in four cyanidiophycean species.**
