# Supplementary material for: Genome-wide signatures of adaptation to extreme environments in red algae
Source: Nat Commun. 2023 Jan 4;14:10. doi: 10.1038/s41467-022-35566-x (PMC9812998; doi:10.1038/s41467-022-35566-x)
Supplement: Supplementary file 5 — Reporting Summary [file 41467_2022_35566_MOESM5_ESM.pdf]

nature portfolio

Corresponding author(s): Hwan Su Yoon

Last updated by author(s): Nov 8, 2022

Reporting Summary

Nature Portfolio wishes to improve the reproducibility of the work that we publish. This form provides structure for consistency and transparency in reporting. For further information on Nature Portfolio policies, see our Editorial Policies and the Editorial Policy Checklist.

Statistics

For all statistical analyses, confirm that the following items are present in the figure legend, table legend, main text, or Methods section.

n/a	Confirmed	
☐	☒	The exact sample size (*n*) for each experimental group/condition, given as a discrete number and unit of measurement
☒	☐	A statement on whether measurements were taken from distinct samples or whether the same sample was measured repeatedly
☐	☒	The statistical test(s) used AND whether they are one- or two-sided<br>*Only common tests should be described solely by name; describe more complex techniques in the Methods section.*
☒	☐	A description of all covariates tested
☒	☐	A description of any assumptions or corrections, such as tests of normality and adjustment for multiple comparisons
☐	☒	A full description of the statistical parameters including central tendency (e.g. means) or other basic estimates (e.g. regression coefficient) AND variation (e.g. standard deviation) or associated estimates of uncertainty (e.g. confidence intervals)
☐	☒	For null hypothesis testing, the test statistic (e.g. *F*, *t*, *r*) with confidence intervals, effect sizes, degrees of freedom and *P* value noted<br>*Give P values as exact values whenever suitable.*
☒	☐	For Bayesian analysis, information on the choice of priors and Markov chain Monte Carlo settings
☒	☐	For hierarchical and complex designs, identification of the appropriate level for tests and full reporting of outcomes
☒	☐	Estimates of effect sizes (e.g. Cohen's *d*, Pearson's *r*), indicating how they were calculated

Our web collection on statistics for biologists contains articles on many of the points above.

Software and code

Policy information about availability of computer code

Data collection — In this study, no software was used to collect data.

Data analysis — Whole genome sequencing (WGS) and whole transcriptome sequencing (WTS).
Adapter and quality trimming for Illumina sequencing reads were conducted using Trimmotatic v0.36 with parameter settings of 'ILLUMINACLIP:TruSeq3-PE.fa:2:30:10:2:keepBothReads LEADING:3 TRAILING:3 MINLEN:100'.

Genome size estimation and genome assembly.
We chose different approaches for genome assembly of individual species due to the differences in sequencing methods and assembly performances. Although the basic outline of the assembly process is consistent across species, we have used multiple platforms and methods to improve the quality of each species' assembly. The basic outline of assembly is as follows: i) build a draft assembly using long-read sequencing platforms (e.g., PacBio, Nanopore) applying multiple assemblers (e.g., HGAP, CANU, FALCON, MaSuRCA), ii) sort out organelle genomes (e.g., mitochondria, chloroplast) to get nuclear genome assembly only, iii) use additional scaffolding method (e.g., RaGOO) based on reference assembly or manually complement non-covering regions from other assemblers, iv) use haplo-merging tools (e.g., Purge-Dups, Purge Haplotigs) to remove duplicated regions that are not considered necessary in a haploid genome, v) correct assembled genome using Illumina reads (e.g., Bowtie2, Pilon) and assess chimeric region based on mapping coverage of reads.
For Cyanidium caldarium 063 E5, we have only used two assemblers, MaSuRCA v3.4.2 was used for main genome and Miniasm v0.3-r179 were used for complementing regional difference between two assembled contigs. RaGOO v1.1 with long read validation were used for contig scaffolding to finalize scaffolds and purge-dup v1.2.5 was used to remove haplotigs. Finally, final 20 chromosomes were recovered from scaffolding process, and chromosome sequences were processed for error correction with pre-processed short-read data using Bowtie2 v2.3.4.1 ('very-sensitive' option) and Pilon v1.23. We repeated this correction step until no conflict sequence was found between corrected and query genomes.
Draft genome of Cyanidiococcus yangmingshanensis 8.1.23 F7 was assembled using HGAP4 implied in PacBio SMRT portal and we compared the result with FALCON-Unzip v1.8.1 assembly result. Organelle genomes were sorted out from assembled genomes using previously

established plastid genomes and mitogenomes. We were able to recover 20 chromosomes from HGAP4 result without using any scaffolding process, and FALCON contigs were used to refine uncovered subtelomere regions. Genome correction, as done in Cyanidium, was used to fine-tune the genome sequence after recovering Cyanidiococcus chromosomes.

Besides hybrid assembly using different platform sequencing couldn't give us a clear assembly result of Galdieria genome, we used different combination of data for assembly; i) FALCON assembler v0.3.0 using PacBio HiFi reads, ii) Nanopore sequencing-based CANU v2.2 assembly, iii) PacBio RS II-based HGAP3, and iv) MaSuRCA v3.2.4 (PacBio and Illumina hybrid assembly). The basic structure of the Galdieria sulphuraria 108.79 E11 genome was built using HiFi result, and other assemblers were used for genome scaffolding and obtaining unique gene regions that HiFi assembly didn't cover. Because Galdieria genome has higher heterozygosity than other Cyanidiales lineages, we used different correction tools (e.g., Pilon v1.2.4, NextPolish v1.2.3, Hapo-G v1.0) using Illumina and PacBio HiFi reads with multiple replications for genome polishing. In addition, due to small chromosome size and duplicated regions across chromosomes, it was hard to discriminate or pair each chromosome to make haploid genome. As a result, we decided to include a few overlapping chromosomal contigs (e.g., haplotigs) in the Galdieria genome like as a pan-genome concept.

PacBio reads were mapped to assembled genomes by minimap v2.17-r941 after all genomes were constructed, and WGSCoveragePlotter was used to visualize mapping coverage of each species. We have used Jellyfish v2.2.8 and KMC v2.3.0 to counting k-mers and estimated genome size using GenomeScope 2.0. When compared to estimated genome size using k-mers, the assembled genome covered at least 90% of the predicted size.

Gene modeling and annotation.
After reconstruction of genomes, we mapped Illumina RNA-Seq and PacBio Iso-Seq data by STAR(long) v2.7.5a to identify transcribed regions from genome data. Transcriptome-mapped data (e.g., Illumina RNA-Seq, PacBio Iso-Seq) was used for the training set of ab initio gene modeling and BRAKER v2.1.6 and GeMoMa v1.7.1 were performed for the gene annotation. However, unlike Galdieria species, BRAKER-based gene annotation did not work well with Cyanidiales genomes due to Cyanidiales unique gene features (e.g., intron-poor gene, short intergenic region). Considering these features, we used Augustus v3.3.1 for ab initio modeling based on BUSCO training sets and Exonerate v2.4.0 for homology-based gene prediction using reference proteins of Cyanidioschyzon and Cyanidiococcus. Combining all gene modeling result with RNA-Seq and Iso-Seq mapping information, we finalized and corrected gene modeling by manual inspection of integrated information (e.g., ab initio gene modeling, reference proteome homology-based gene modeling, transcript-mapped regions) in all three species. Additionally, some of the putatively mispredicted genes in the Galdieria genome (approximately 70 genes) were manually removed based on two criteria: i) exclusive intron patterns without support from RNA-seq and Iso-Seq data, ii) no homology with other proteins and lack of a function domain inside the protein. The completeness of gene modeling was verified by BUSCO v3.0.2 using general eukaryote database ('eukaryota_odb9'). Despite the availability of a more recent BUSCO database ('eukaryota odb10, n:303'; 21.1% missing BUSCOs in Cyanidioschyzon 10D), we chose to use previous version database ('eukaryota odb9, n:255'; 3.6% missing BUSCOs in Cyanidioschyzon 10D) because recent version contains many missing genes that were lost in the cyanidiophycean lineage tested by reference genome (Cyanidioschyzon 10D). We used multiple methods for functional annotations of genes in each species: i) BLAST-based search (e.g., MMSeqs2, DIAMOND) against NCBI nr protein database, ii) HMMER-based search against a customized HMM database of KEGG Orthologs using KofamKOALA (ver. 2021-03-01), iii) BLAST-based search using eggNOG v5.0, which is specialized database for functional annotation. For functional RNA annotation, we applied Infernal v1.1.2 using Rfam v12.5 (March 2021, 3940 families) database. Transcription start site prediction were identified by TSSPlant with a support of in-house python script.

Analysis of repeats in genomes.
Following the analysis pipeline used in a previous study, repeat sequences in genomes were identified using the de novo method using the RepeatModeler v2.0.2a (http://www.repeatmasker.org/RepeatModeler). We used 13 and 14 l-mers optimized by round from 'log4[genome size] + 1' for repeat analysis and classified into repeat subclasses using RepBase (updated October 26th, 2018) and Dfam v3.3 (November 09th, 2020) database. Genetic distance between repeat copies found in genomic sequences were parsed from the output of RepeatMasker v4.1.2-p1 and used to measure the Kimura's distance.

Genome analysis.
Nucleotide sequence alignment-based genome comparison were performed by JupiterPlot v1.0 (https://github.com/JustinChu/JupiterPlot) to see structural variation. However, nucleotide alignment-based genome comparison between cyanidiophycean species had insufficient resolution, so we conducted gene synteny-based comparison for higher level of taxonomy. Cyanidiophyceae genomes were compared using synteny blocks identified by MCScanX with a minimum syntenic block length of five genes and a maximum gap between genes in a syntenic block of 25 genes. Tree view mode of SynVisio was used to visualize the results of the synteny block comparison. To identify subtelomeric regions from genomes, LASTZ alignment v7.0.2 were used to see if there were any conserved regions between chromosomes.
Following the analysis pipeline used in a previous study, repeat sequences in genomes were identified using the de novo method using the RepeatModeler v2.0.2a (http://www.repeatmasker.org/RepeatModeler). We used 13 and 14 l-mers optimized by round from 'log4[genome size] + 1' for repeat analysis and classified into repeat subclasses using RepBase (updated October 26th, 2018) and Dfam v3.3 (November 09th, 2020) database. Genetic distance between repeat copies found in genomic sequences were parsed from the output of RepeatMasker v4.1.2-p1 and used to measure the Kimura's distance.

The grouping of orthologous genes was performed by Orthofinder v2.5.2 with default option and protein dataset were collected from 35 representative taxa of Archaeplastida. Gene gain and loss event of cyanidiophycean algae were tested by the Dollo parsimony method (DolloP) using Archaeplastida-based orthogroups. We've used this orthogroup information for further analysis of gene families, however, two major issues have arisen: i) some misannotated genes found in individual strains combine two independent gene families into one orthogroup that has no functional domain in common but is clustered together by a misannotated fused gene, and ii) some orthogroups were separated due to protein properties (e.g., highly diverged protein, small size protein) due to a unified parameter adjusted for all different gene families. We were not able to discard some of problematic genes from whole orthogroups because we don't have strong evidence to reject published gene modeling data. Therefore, we manually confirmed controversial genes that appeared to be misannotated compared to sister species or strains (i.e., parsimonious approach) for further analysis.

TargetP v1.1 101 and DeepTMHMM v1.0.1 102 were used to predict transit peptides and transmembrane domain regions in order to validate gene localization.

Phylogenetic analysis of genes.
To determine the evolutionary history of target genes, we obtained homologous protein sequences from the NCBI non-redundant protein sequence database by applying protein homologous searches using MMSeqs2 v13.45111. Sequences collected for phylogenetic analysis were aligned using MAFFT v7.310, and some alignments with a lot of gaps were trimmed out using trimAl v1.4 '-automated1' option. IQ-TREE v2.1.2 was used for Maximum Likelihood (ML) inference of phylogenetic tree. To select evolutionary models, implemented model selection was used, and 1,000 replications of the ultrafast bootstrap approximation approach (UFBoot2) were used for phylogenetic analysis. After phylogenetic trees were constructed, we removed a few taxa that seem to be redundant due to dataset disequilibrium of taxon sampling

(e.g., extensively sequenced in a particular lineage). Following the removal of redundant taxa, we reanalyzed the datasets, beginning with the alignment and performing the phylogenetic analysis as previously stated. The final trees were visualized using FigTree v1.4.4 with a midpoint root or an unrooted tree if outgroups were not considered from the start.

Analysis of histone modification ChIP-Seq data.
We used previously sequenced ChIP-Seq data [Input DNA, histone H3 (H3), and tri-methylation of lysine 27 on histone H3 (H3K27me3)] from Cyanidioschyzon merolae 10D to confirm H3K27me3 histone modification pattern in Cyanidiophyceae. All ChIP-Seq data were mapped against the Cyanidioschyzon genome using Bowtie2 (v2.3.4.1), and the peaks were identified with Model Based Analysis of ChIP-seq data (MACS3 v3.0.0a7). Input DNA data was used as a control for both H3K27me3 and H3. Enrichment of H3K27me3 peaks refer to the MACS3-calculated log fold changes over H3 and we used calculated fold-enrichment information for further analysis. We used IGV v2.11.0 for visualizing the output findings of "broadPeak" and "gappedPeak," which were signal enrichment based on pooled and normalized data.

Non-synonymous substitutions per non-synonymous sites (Ka) and synonymous substitutions per synonymous sites analysis.
To assess evolutionary selection of subtelomeric duplicated genes, each of subtelomeric duplicated genes were aligned by MAFFT v7.471. Ka/Ks analysis were performed using ParaAT v2.0 and KaKs_Calculator v2.0.

Characterization of polycistronic transcripts.
We used deduplicated high-quality transcripts from PacBio Iso-Seq circular consensus sequencing (CCS) reads to identify polycistronic transcripts, and all transcripts were mapped to the genome using STARlong v2.7.5a. Using gene modeling information and mapped information, polycistronic transcripts were identified by an in-house python script based on the criteria of completely covering at least two gene regions in the same direction as the transcript. After identifying of polycistronic loci, internal ribosome entry sites (IRESs) were identified from all putative polycistronic transcripts using IRESfinder.

Protein homeostasis survey.
Hidden Markov models (HMMs) of different chaperone types (Hsp20, 40, 60, 70, 90, and 100) were used for hmmsearch were performed for profiling chaperones in our data. TANGO v2.3.1 was used for estimating proteins aggregation propensity based on protein sequences and hydrophobicity of proteomes was computed with the Kyte–Doolittle hydropathy scale using python and R scripts (https://github.com/pechmannlab/chapevo). Statistical tests were done with R packages providing t-test and others.

For manuscripts utilizing custom algorithms or software that are central to the research but not yet described in published literature, software must be made available to editors and reviewers. We strongly encourage code deposition in a community repository (e.g. GitHub). See the Nature Portfolio guidelines for submitting code & software for further information.

Data

Policy information about availability of data

All manuscripts must include a data availability statement. This statement should provide the following information, where applicable:

- Accession codes, unique identifiers, or web links for publicly available datasets
- A description of any restrictions on data availability
- For clinical datasets or third party data, please ensure that the statement adheres to our policy

All sequencing data and assembled genomes are deposited in the NCBI database as BioProject PRJNA851236 (SRA: SRR19760108-SRR19760122; Biosample: SAMN29217976-SAMN29217978; Assembly: JANCYU000000000, JANCYV000000000, JANCYW000000000). The DRYAD database (https://doi.org/10.5061/dryad.cfxpnvx7b) contains all the analyzed data used in this study.

Field-specific reporting

Please select the one below that is the best fit for your research. If you are not sure, read the appropriate sections before making your selection.

☐ Life sciences ☐ Behavioural & social sciences ☒ Ecological, evolutionary & environmental sciences

For a reference copy of the document with all sections, see nature.com/documents/nr-reporting-summary-flat.pdf

Ecological, evolutionary & environmental sciences study design

All studies must disclose on these points even when the disclosure is negative.

Study description	Using chromosomal-level genome assemblies, we studied the evolution of extremophilic red algae. We find that horizontal gene transfer is essential for adaptation to extreme hot-springs environments, but that other innovations such as subtelomeric gene duplications and loss of conserved processes, such as microRNA biogenesis, also characterize the extremophilic lifestyle. Two major Cyanidiophyceae orders, the Cyanidiales and Galdieriales, have evolved distinct strategies for adaptation, suggesting that massive genome divergence can occur, even when cells live near each other, but face very different selective pressures based on varying temperature, acidity, and nutrient levels.
Research sample	Three Cyanidiophyceae genomes were constructed using culture samples. To eliminate mixed cryptic species in a culture strain, we established culture strains from single cells using the fluorescence-activated cell sorting (FACS) method: Cyanidium caldarium 063 E5 was isolated from DBV 063 strain, Cyanidiococcus yangmingshanensis 8.1.23 F7 was isolated from Galdieria maxima (now Cyanidiococcus yangmingshanensis) 8.1.23 strain, and Galdieria sulphuraria 108.79 E11 was isolated from SAG 108.79 strain. After initial cultivation in 96 well plates, the mass culture was done in modified 5x Allen’s medium. More information about these samples can be found in a prior study (Cho et al., 2020, BMC Evol Biol).
Sampling strategy	Heavy metal treatments

Sampling strategy

A modified Allen's medium with serial concentrations of each metal (0, 1, 10, 25, 50, and 100 mM, pH = 2) was used to test the arsenite [As(III); $NaAsO_2$, CAS #7784-46-5, Sigma-Aldrich] and arsenate [As(V); $Na_2HAsO_4 \cdot 7H_2O$, CAS #10048-95-0, Sigma-Aldrich] tolerance of Cyanidium caldarium, Cyanidiococcus yangmingshanensis, and Galdieria sulphuraria. Physiological experiments with three biological replicates were conducted with a shaking speed of 130 rpm at 30 °C and a light intensity of 70 µmol/m2·s at a 12:12h light-dark cycle for 7 days. On the first day, cell density was diluted to OD750 as 0.05 to standardize the beginning condition. OD750 was measured using xMark™ Microplate Absorbance Spectrophotometer (Bio-Rad, Hercules, USA) on the first and the seventh days of the experiment.

Data collection

For genome and transcriptome sequencing, both short-read and long-read sequencing were conducted by Chung Hyun Cho, Seung In Park, Tzu-Yen Huang, Yongsung Lee. For PacBio whole genome sequencing (WGS), we used SMRTbell® Express Template Prep Kit 2.0 (Pacific Biosciences, Menlo Park, CA, USA) with a 15 kbp size selection to construct Sequel I sequencing libraries of Cyanidium and Cyanidiococcus. For Galdieria PacBio WGS, SMRTbell® Express Template Prep Kit 1.0 (Pacific Biosciences) with a 9 kbp size selection was used to prepare the RS II sequencing library and SMRTbell Express TPK 2.0 (Pacific Biosciences) was used for HiFi library preparation. All experiments followed the manufacturer's standard protocol, without shearing step in Cyanidium and Galdieria samples. SQK-LSK109 ligation kit (Oxford Nanopore Technologies, Oxford, UK) was used to construct a library of Galdieria PromethION sequencing without shearing step and a 20 kbp size selection. For Illumina HiSeq2500 WGS of Cyanidium and Galdieria species, TruSeq® Nano DNA Prep Kit (Illumina, San Diego, CA, USA) with an insert size 550 bp was used to prepare gDNA sequencing libraries. The same kit and protocol were used for Cyanidiococcus WGS and ran in the Illumina NovaSeq6000 platform. SMARTer PCR cDNA Synthesis Kit (Clontech Laboratories, Palo Alto, CA, USA) and SMRTbell® Express Template Prep Kit 1.0 (Pacific Biosciences) were used to prepare PacBio WTS (Iso-Seq) libraries. Clustering and deduplication of Iso-Seq reads were done by IsoSeq v3 implemented in Sequel SMRT® Link v8.0 and high-quality reads (99% accuracy) from clustered results were only used for subsequent analysis. For Illumina WTS (RNA-Seq), TruSeq® Stranded mRNA Prep Kit (Illumina) were used for library construction for all species and those libraries were sequenced with Illumina NovaSeq6000 platform.

Timing and spatial scale

[N/A] In this study, there was no experiment to require timing and spatial scale.

Data exclusions

[N/A] No data were excluded from the analyses.

Reproducibility

The data is all publicly available online in the NCBI database and DRYAD database. All bioinformatic analysis software was released under an open-source license and is freely available.

Randomization

[N/A] This is not relevant as our study does not consider variable assignments or categories.

Blinding

[N/A] This study did not include any blinding information, which is not required for any analysis.

Did the study involve field work? ☐ Yes ☒ No

Reporting for specific materials, systems and methods

We require information from authors about some types of materials, experimental systems and methods used in many studies. Here, indicate whether each material, system or method listed is relevant to your study. If you are not sure if a list item applies to your research, read the appropriate section before selecting a response.

Materials & experimental systems

n/a	Involved in the study
☒	☐ Antibodies
☒	☐ Eukaryotic cell lines
☒	☐ Palaeontology and archaeology
☒	☐ Animals and other organisms
☒	☐ Human research participants
☒	☐ Clinical data
☒	☐ Dual use research of concern

Methods

n/a	Involved in the study
☒	☐ ChIP-seq
☐	☒ Flow cytometry
☒	☐ MRI-based neuroimaging

Flow Cytometry

Plots

Confirm that:

☒ The axis labels state the marker and fluorochrome used (e.g. CD4-FITC).

☐ The axis scales are clearly visible. Include numbers along axes only for bottom left plot of group (a 'group' is an analysis of identical markers).

☐ All plots are contour plots with outliers or pseudocolor plots.

☐ A numerical value for number of cells or percentage (with statistics) is provided.

Methodology

Sample preparation	To eliminate mixed cryptic species in a culture strain, we established culture strains from cells using the fluorescence-activated cell sorting (FACS) method: Cyanidium caldarium 063 E5 was isolated from DBV 063 strain, Cyanidiococcus yangmingshanensis 8.1.23 F7 was isolated from Galdieria maxima (now Cyanidiococcus yangmingshanensis) 8.1.23 strain, and Galdieria sulphuraria 108.79 E11 was isolated from SAG 108.79 strain. After initial cultivation in 96 well plates, the mass culture was done in modified 5x Allen's medium.
Instrument	Sony SH800S
Software	Sony SH800S software
Cell population abundance	Minor cell populations and cell debris in medium were distinguished based on relative cell size and granularity as determined by the FSC-H, FSC-A, and SSC signals. The autofluorescence signal of chloroplasts was detected using PE-Cy5 to distinguish between larger cell debris and dead cell signals. Because the purpose was to isolate single cells rather than extract a cell population displaying a specific signal, we did not specify cell population.
Gating strategy	To identify specific live Cyanidiophyceae cell populations, cells in gates FSC (374-396 nm) and SSC (407-456 nm) were backgated onto PE-Cy5 (>500 nm).

☒ Tick this box to confirm that a figure exemplifying the gating strategy is provided in the Supplementary Information.
