# Supplementary material for: Genome-wide signatures of adaptation to extreme environments in red algae
Source: Nat Commun. 2023 Jan 4;14:10. doi: 10.1038/s41467-022-35566-x (PMC9812998; doi:10.1038/s41467-022-35566-x)
Supplement: Supplementary file 6 — Source Data [file 41467_2022_35566_MOESM6_ESM.zip › pdf files/Supplementary Figure S15abc - miRNA processing AGO DCL 221109.pdf]

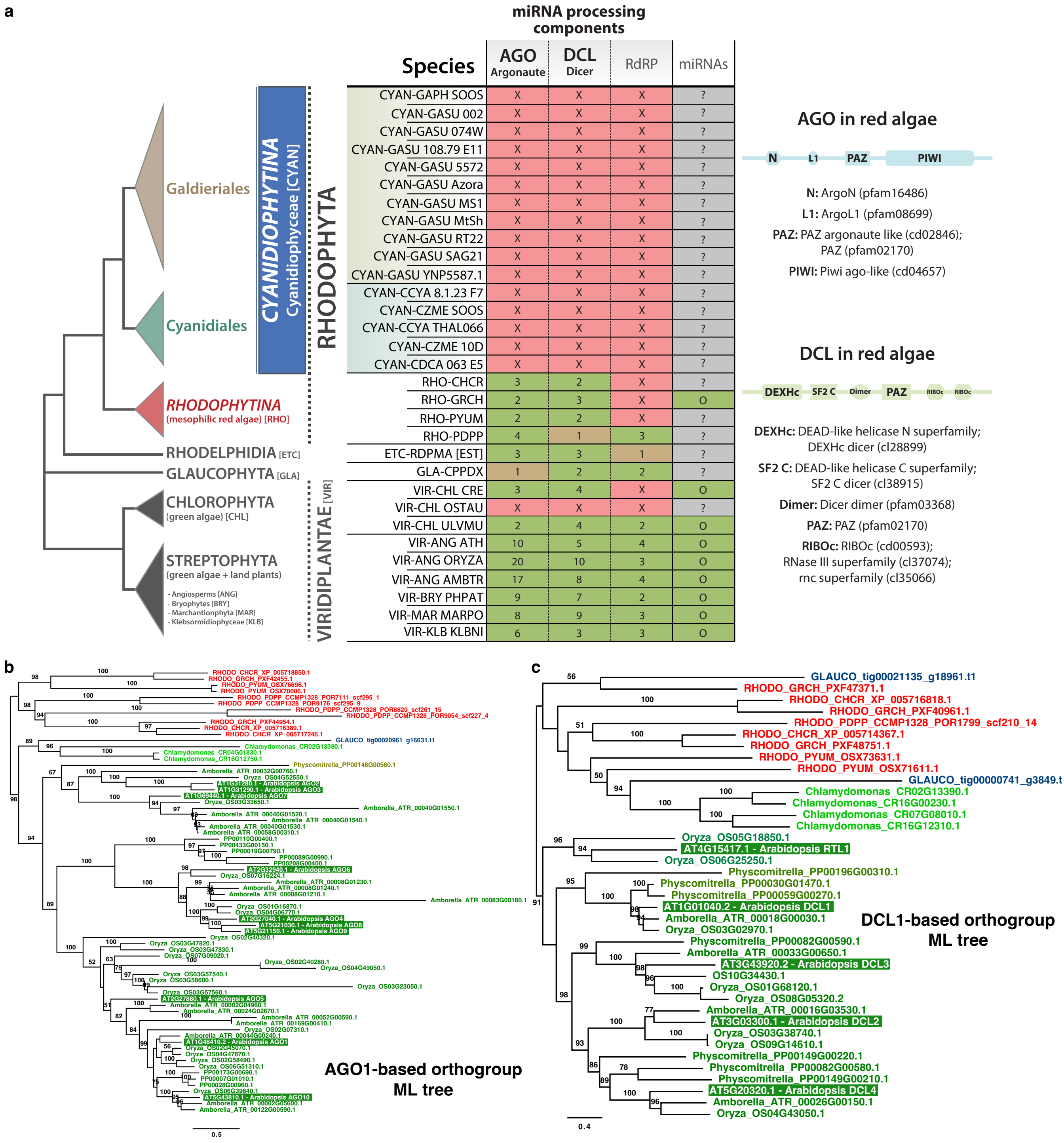
a
miRNA processing components
Species
AGO Argonaute
DCL Dicer
RdRP
miRNAs
CYAN-GAPH SOOS X X X ?
CYAN-GASU 002 X X X ?
CYAN-GASU 074W X X X ?
CYAN-GASU 108.79 E11 X X X ?
CYAN-GASU 5572 X X X ?
CYAN-GASU Azora X X X ?
CYAN-GASU MS1 X X X ?
CYAN-GASU MtSh X X X ?
CYAN-GASU RT22 X X X ?
CYAN-GASU SAG21 X X X ?
CYAN-GASU YNP5587.1 X X X ?
CYAN-CCYA 8.1.23 F7 X X X ?
CYAN-CZME SOOS X X X ?
CYAN-CCYA THAL066 X X X ?
CYAN-CZME 10D X X X ?
CYAN-CDCA 063 E5 X X X ?
RHO-CHCR 3 2 X ?
RHO-GRCH 2 3 X O
RHO-PYUM 2 2 X ?
RHO-PDPP 4 1 3 ?
ETC-RDPMA [EST] 3 3 1 ?
GLA-CPPDX 1 2 2 ?
VIR-CHL CRE 3 4 X O
VIR-CHL OSTAU X X X ?
VIR-CHL ULVMU 2 4 2 O
VIR-ANG ATH 10 5 4 O
VIR-ANG ORYZA 20 10 3 O
VIR-ANG AMBTR 17 8 4 O
VIR-BRY PHPAT 9 7 2 O
VIR-MAR MARPO 8 9 3 O
VIR-KLB KLBNI 6 3 3 O
Galdieriales
Cyanidiales
CYANIDIOPHYTINA
Cyanidiophyceae [CYAN]
RHODOPHYTA
RHODOPHYTINA
(mesophilic red algae) [RHO]
RHODELPHIDIA [ETC]
GLAUCOPHYTA [GLA]
CHLOROPHYTA
(green algae) [CHL]
STREPTOPHYTA
(green algae + land plants)
- Angiosperms [ANG]
- Bryophytes [BRY]
- Marchantionphyta [MAR]
- Klebsormidiophyceae [KLB]
VIRIDIPLANTAE [VIR]
AGO in red algae
N
L1
PAZ
PIWI
N: ArgoN (pfam16486)
L1: ArgoL1 (pfam08699)
PAZ: PAZ argonaute like (cd02846); PAZ (pfam02170)
PIWI: Piwi ago-like (cd04657)
DCL in red algae
DEXHc
SF2 C
Dimer
PAZ
RIBOc
RIBOc
DEXHc: DEAD-like helicase N superfamily; DEXHc dicer (cl28899)
SF2 C: DEAD-like helicase C superfamily; SF2 C dicer (cl38915)
Dimer: Dicer dimer (pfam03368)
PAZ: PAZ (pfam02170)
RIBOc: RIBOc (cd00593); RNase III superfamily (cl37074); rnc superfamily (cl35066)
b
AGO1-based orthogroup ML tree
0.5
c
GLAUCO_tig00021135_g18961.t1
RHODO_GRCH_PXF47371.1
RHODO_CHCR_XP_005716818.1
RHODO_GRCH_PXF40961.1
RHODO_PDPP_CCMP1328_POR1799_scf210_14
RHODO_CHCR_XP_005714367.1
RHODO_GRCH_PXF48751.1
RHODO_PYUM_OSX73631.1
RHODO_PYUM_OSX71611.1
GLAUCO_tig00000741_g3849.t
Chlamydomonas_CR02G13390.1
Chlamydomonas_CR16G00230.1
Chlamydomonas_CR07G08010.1
Chlamydomonas_CR16G12310.1
Oryza_OS05G18850.1
AT4G15417.1 - Arabidopsis RTL1
Oryza_OS06G25250.1
Physcomitrella_PP00196G00310.1
Physcomitrella_PP00030G01470.1
Physcomitrella_PP00059G00270.1
AT1G01040.2 - Arabidopsis DCL1
Amborella_ATR_00018G00030.1
Oryza_OS03G02970.1
Physcomitrella_PP00082G00590.1
Amborella_ATR_00033G00650.1
AT3G43920.2 - Arabidopsis DCL3
OS10G34430.1
Oryza_OS01G68120.1
Oryza_OS08G05320.2
Amborella_ATR_00016G03530.1
AT3G03300.1 - Arabidopsis DCL2
Oryza_OS03G38740.1
Oryza_OS09G14610.1
Physcomitrella_PP00149G00220.1
Physcomitrella_PP00082G00580.1
Physcomitrella_PP00149G00210.1
AT5G20320.1 - Arabidopsis DCL4
Amborella_ATR_00026G00150.1
Oryza_OS04G43050.1
DCL1-based orthogroup ML tree
0.4
