# Supplementary material for: Genome-wide signatures of adaptation to extreme environments in red algae
Source: Nat Commun. 2023 Jan 4;14:10. doi: 10.1038/s41467-022-35566-x (PMC9812998; doi:10.1038/s41467-022-35566-x)
Supplement: Supplementary file 6 — Source Data [file 41467_2022_35566_MOESM6_ESM.zip › pdf files/Supplementary Figure S22 - FACS_isolation IC.pdf]

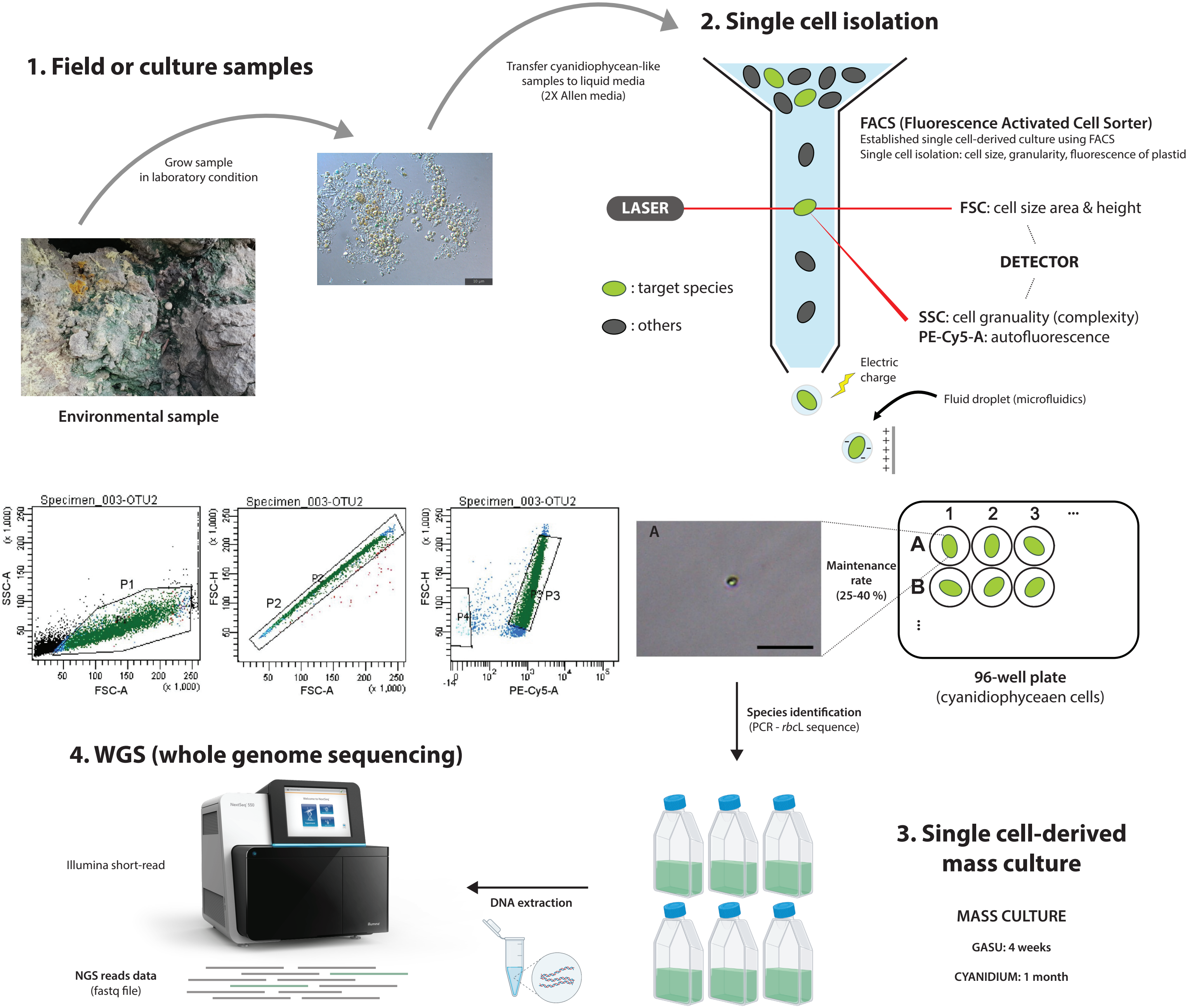
1. Field or culture samples
Grow sample
in laboratory condition
Environmental sample
Transfer cyanidiophycean-like
samples to liquid media
(2X Allen media)
2. Single cell isolation
FACS (Fluorescence Activated Cell Sorter)
Established single cell-derived culture using FACS
Single cell isolation: cell size, granularity, fluorescence of plastid
LASER
FSC: cell size area & height
DETECTOR
SSC: cell granuality (complexity)
PE-Cy5-A: autofluorescence
: target species
: others
Electric
charge
Fluid droplet (microfluidics)
Specimen_003-OTU2
SSC-A
FSC-A
P1
Specimen_003-OTU2
FSC-H
FSC-A
P2
Specimen_003-OTU2
FSC-H
PE-Cy5-A
P3
P4
A
Maintenance
rate
(25-40 %)
1
2
3
A
B
96-well plate
(cyanidiophyceaen cells)
Species identification
(PCR - rbcL sequence)
3. Single cell-derived
mass culture
MASS CULTURE
GASU: 4 weeks
CYANIDIUM: 1 month
DNA extraction
4. WGS (whole genome sequencing)
Illumina short-read
NGS reads data
(fastq file)
