# Supplementary material for: Genome-wide signatures of adaptation to extreme environments in red algae
Source: Nat Commun. 2023 Jan 4;14:10. doi: 10.1038/s41467-022-35566-x (PMC9812998; doi:10.1038/s41467-022-35566-x)
Supplement: Supplementary file 6 — Source Data [file 41467_2022_35566_MOESM6_ESM.zip › pdf files/Supplementary Figure S1 - CCYA strain comparison updated.pdf]

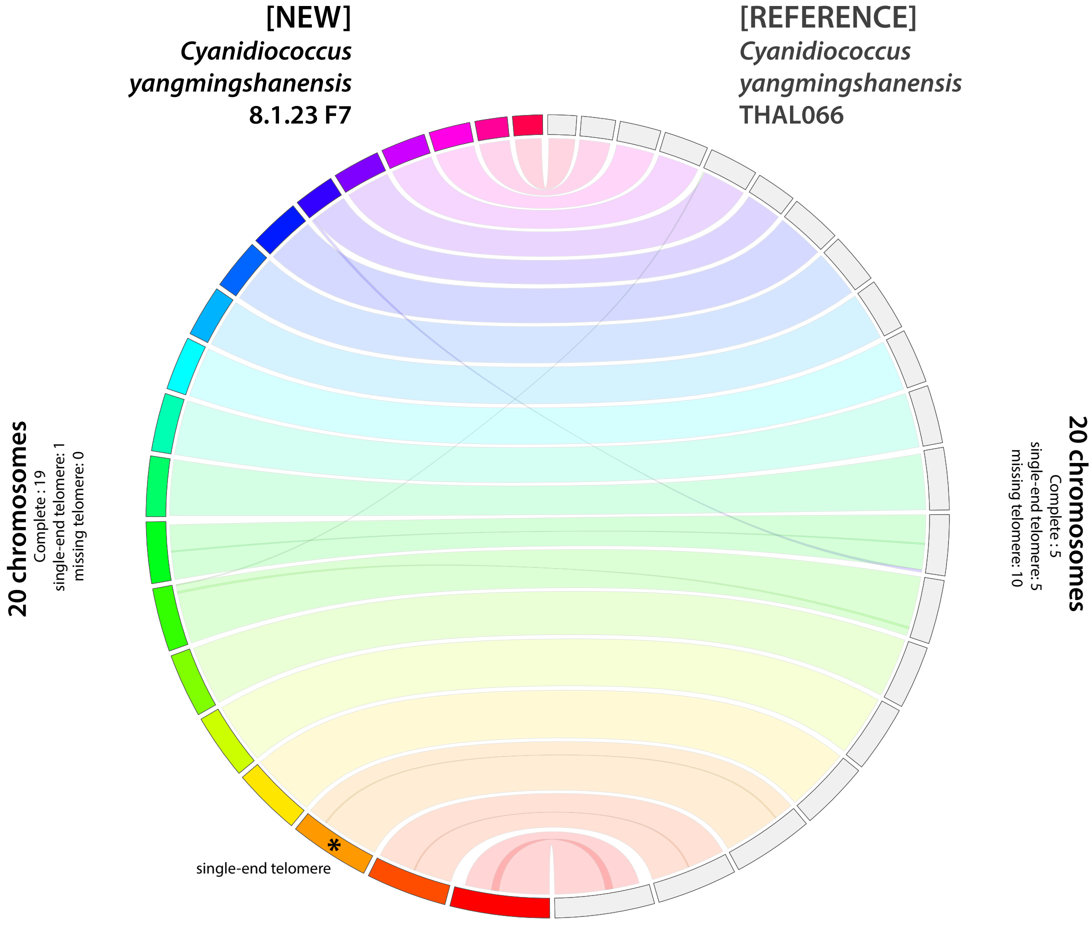
[NEW]
Cyanidiococcus
yangmingshanensis
8.1.23 F7
[REFERENCE]
Cyanidiococcus
yangmingshanensis
THAL066
20 chromosomes
Complete : 19
single-end telomere: 1
missing telomere: 0
20 chromosomes
Complete : 5
single-end telomere: 5
missing telomere: 10
*
single-end telomere
