# Supplementary material for: Genome-wide signatures of adaptation to extreme environments in red algae
Source: Nat Commun. 2023 Jan 4;14:10. doi: 10.1038/s41467-022-35566-x (PMC9812998; doi:10.1038/s41467-022-35566-x)
Supplement: Supplementary file 6 — Source Data [file 41467_2022_35566_MOESM6_ESM.zip › pdf files/Supplementary Figure S13 - DolloP_COG_Detail_220315.pdf]

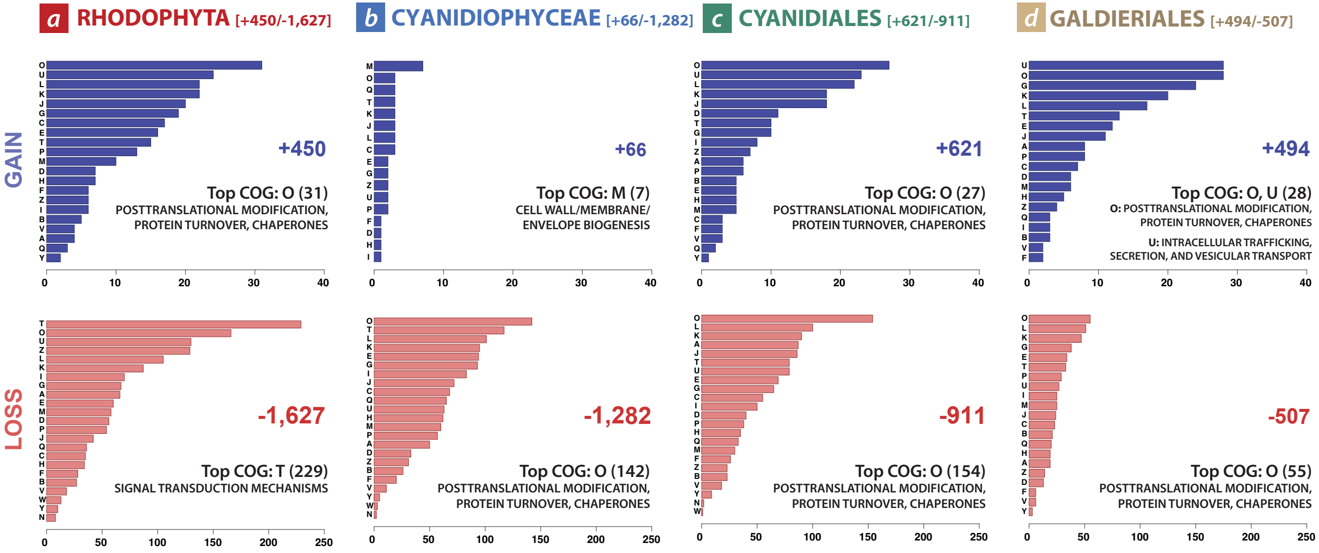

A: RNA PROCESSING AND MODIFICATION
B: CHROMATIN STRUCTURE AND DYNAMICS
C: ENERGY PRODUCTION AND CONVERSION
D: CELL CYCLE CONTROL, CELL DIVISION, CHROMOSOME PARTITIONING
E: AMINO ACID TRANSPORT AND METABOLISM
F: NUCLEOTIDE TRANSPORT AND METABOLISM
G: CARBOHYDRATE TRANSPORT AND METABOLISM
H: COENZYME TRANSPORT AND METABOLISM
I: LIPID TRANSPORT AND METABOLISM
J: TRANSLATION, RIBOSOMAL STRUCTURE AND BIOGENESIS
K: TRANSCRIPTION
L: REPLICATION, RECOMBINATION AND REPAIR
M: CELL WALL/MEMBRANE/ENVELOPE BIOGENESIS
N: CELL MOTILITY
O: POSTTRANSLATIONAL MODIFICATION, PROTEIN TURNOVER, CHAPERONES
P: INORGANIC ION TRANSPORT AND METABOLISM
Q: SECONDARY METABOLITES BIOSYNTHESIS, TRANSPORT AND CATABOLISM
T: SIGNAL TRANSDUCTION MECHANISMS
U: INTRACELLULAR TRAFFICKING, SECRETION, AND VESICULAR TRANSPORT
V: DEFENSE MECHANISMS
W: EXTRACELLULAR STRUCTURES
Y: NUCLEAR STRUCTURE
Z: CYTOSKELETON
*Excluded "**S:** FUNCTION UNKNOWN"
