# Supplementary material for: Genome-wide signatures of adaptation to extreme environments in red algae
Source: Nat Commun. 2023 Jan 4;14:10. doi: 10.1038/s41467-022-35566-x (PMC9812998; doi:10.1038/s41467-022-35566-x)
Supplement: Supplementary file 6 — Source Data [file 41467_2022_35566_MOESM6_ESM.zip › pdf files/Supplementary Figure S17 - Polycomb unit 221206.pdf]

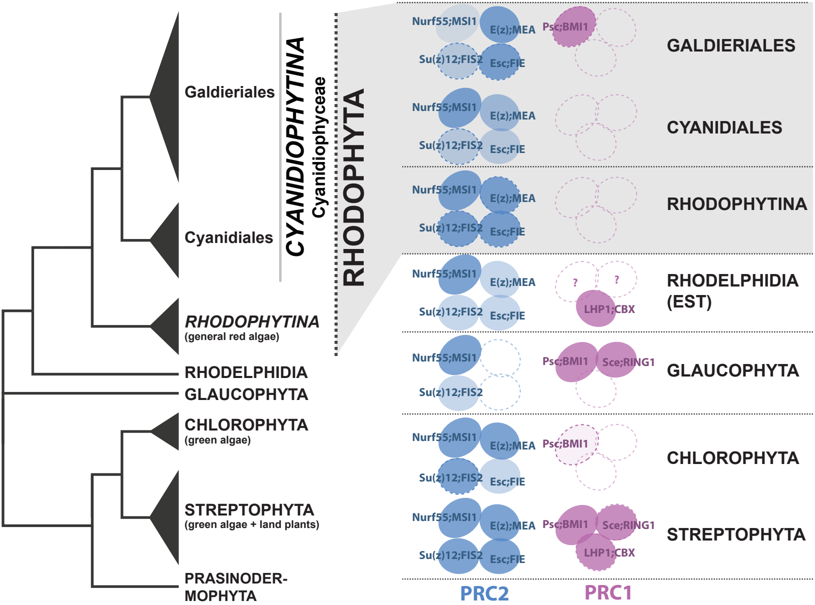

		GALDIER IALES	CYANID IALES	RED ALGAE	RHODEL PHIDIA	GLAUCO PHYTA	CHLORO PHYTA	STREPTO PHYTA
PRC2	Core PRC2 - Nurf55;MSI	1	2	2-3	3	2	2	2-5
	Core PRC2 - E(z);MEA	1-3	1	0-6	1	0	1	1-7
	Core PRC2 - Su(z)12;FIS2	0-1	0-1	0-2	1	0	0-2	1-4
	Core PRC2 - Esc;FIE	0-3	1	0-2	1	1	1	1-2
PRC1	Core PRC1 - BMI1	0-3	0	0	0	2	0-1	1-6
	Core PRC1 - RING1	0	0	0	0	3	0	0-6
	Core PRC1 - LHP1	0	0	0	2	0	0	0-4
