# Supplementary material for: Genome-wide signatures of adaptation to extreme environments in red algae
Source: Nat Commun. 2023 Jan 4;14:10. doi: 10.1038/s41467-022-35566-x (PMC9812998; doi:10.1038/s41467-022-35566-x)
Supplement: Supplementary file 6 — Source Data [file 41467_2022_35566_MOESM6_ESM.zip › pdf files/Supplementary Figure S23abc - GASU_CCYA_CYCA_WGScoverage_220621_modified.pdf]

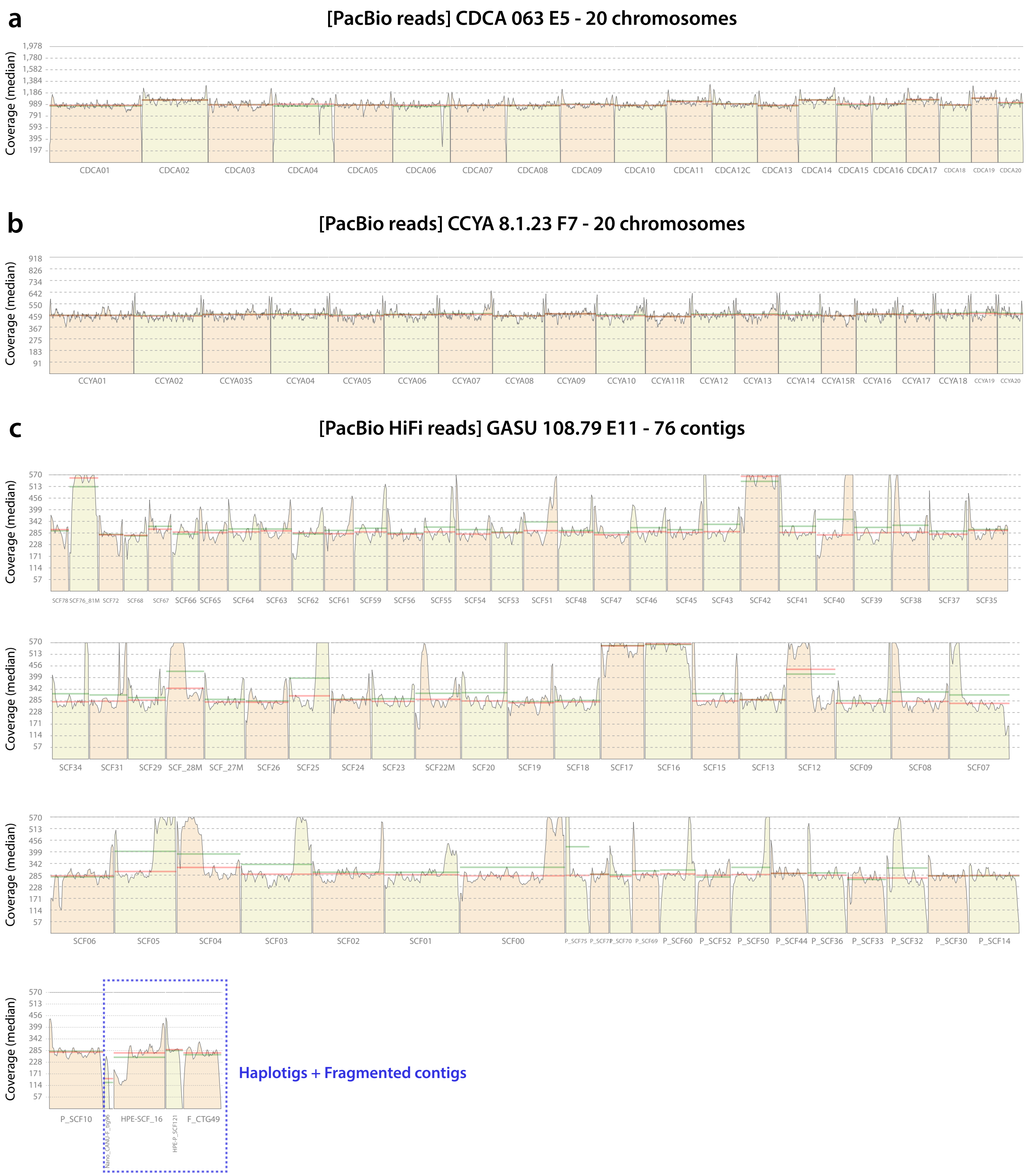
a
[PacBio reads] CDCA 063 E5 - 20 chromosomes
Coverage (median)
1,978
1,780
1,582
1,384
1,186
989
791
593
395
197
CDCA01 CDCA02 CDCA03 CDCA04 CDCA05 CDCA06 CDCA07 CDCA08 CDCA09 CDCA10 CDCA11 CDCA12C CDCA13 CDCA14 CDCA15 CDCA16 CDCA17 CDCA18 CDCA19 CDCA20
b
[PacBio reads] CCYA 8.1.23 F7 - 20 chromosomes
Coverage (median)
918
826
734
642
550
459
367
275
183
91
CCYA01 CCYA02 CCYA03S CCYA04 CCYA05 CCYA06 CCYA07 CCYA08 CCYA09 CCYA10 CCYA11R CCYA12 CCYA13 CCYA14 CCYA15R CCYA16 CCYA17 CCYA18 CCYA19 CCYA20
c
[PacBio HiFi reads] GASU 108.79 E11 - 76 contigs
Coverage (median)
570
513
456
399
342
285
228
171
114
57
SCF78 SCF76_81M SCF72 SCF68 SCF67 SCF66 SCF65 SCF64 SCF63 SCF62 SCF61 SCF59 SCF56 SCF55 SCF54 SCF53 SCF51 SCF48 SCF47 SCF46 SCF45 SCF43 SCF42 SCF41 SCF40 SCF39 SCF38 SCF37 SCF35
SCF34 SCF31 SCF29 SCF_28M SCF_27M SCF26 SCF25 SCF24 SCF23 SCF22M SCF20 SCF19 SCF18 SCF17 SCF16 SCF15 SCF13 SCF12 SCF09 SCF08 SCF07
SCF06 SCF05 SCF04 SCF03 SCF02 SCF01 SCF00 P_SCF75 P_SCF71 P_SCF70 P_SCF69 P_SCF60 P_SCF52 P_SCF50 P_SCF44 P_SCF36 P_SCF33 P_SCF32 P_SCF30 P_SCF14
P_SCF10 Nano_CANU-F_tig96 HPE-SCF_16 HPE-P_SCF121 F_CTG49
Haplotigs + Fragmented contigs
