# Supplementary material for: Genome-wide signatures of adaptation to extreme environments in red algae
Source: Nat Commun. 2023 Jan 4;14:10. doi: 10.1038/s41467-022-35566-x (PMC9812998; doi:10.1038/s41467-022-35566-x)
Supplement: Supplementary file 6 — Source Data [file 41467_2022_35566_MOESM6_ESM.zip › pdf files/Supplementary Figure S2ab - annot error_221206.pdf]

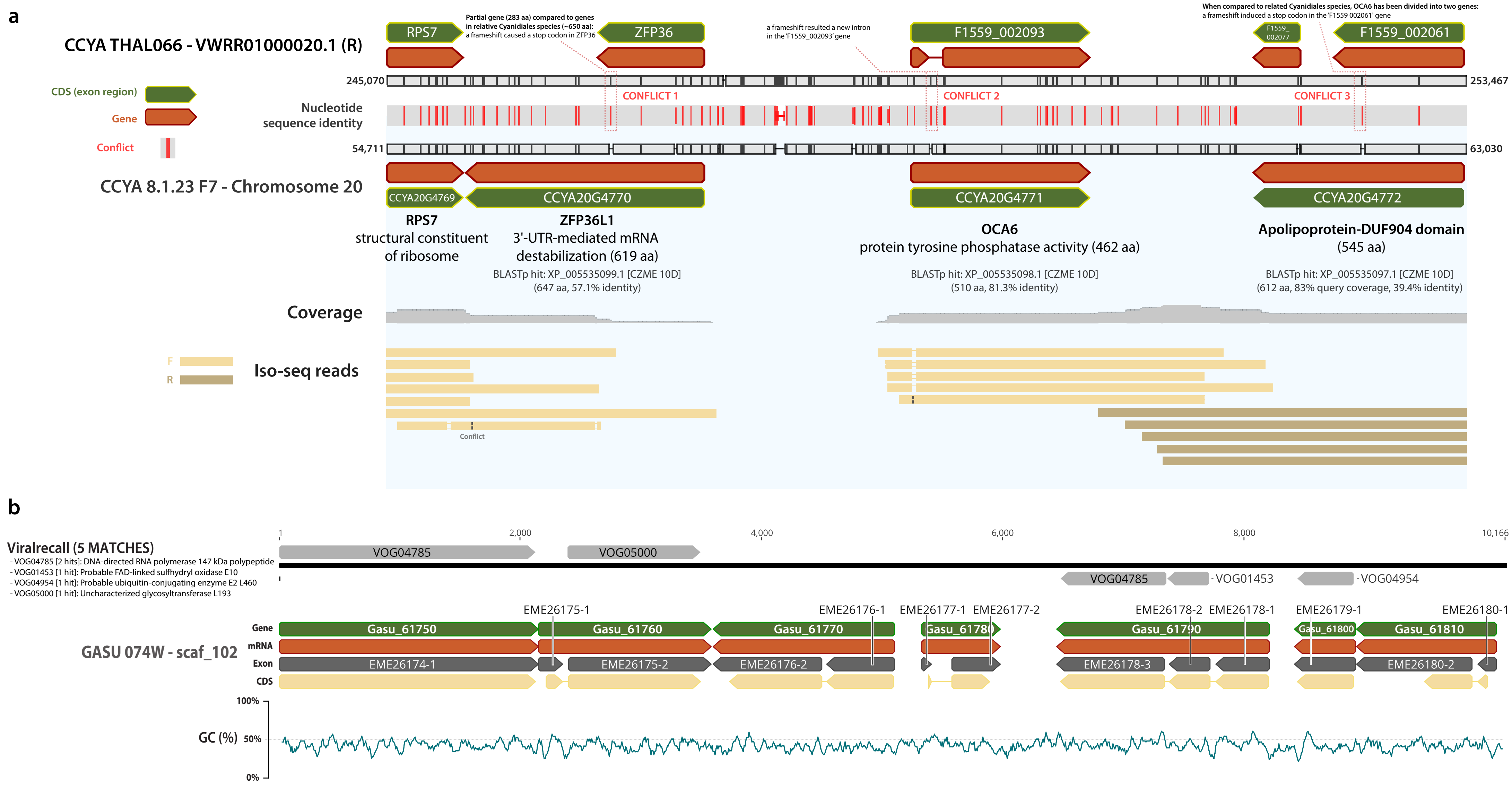
a
CCYA THAL066 - VWRR01000020.1 (R)
RPS7
Partial gene (283 aa) compared to genes in relative Cyanidiales species (~650 aa): a frameshift caused a stop codon in ZFP36
ZFP36
a frameshift resulted a new intron in the 'F1559_002093' gene
F1559_002093
When compared to related Cyanidiales species, OCA6 has been divided into two genes: a frameshift induced a stop codon in the 'F1559 002061' gene
F1559_002077
F1559_002061
245,070
253,467
CONFLICT 1
CONFLICT 2
CONFLICT 3
CDS (exon region)
Gene
Conflict
Nucleotide sequence identity
54,711
63,030
CCYA 8.1.23 F7 - Chromosome 20
CCYA20G4769
CCYA20G4770
CCYA20G4771
CCYA20G4772
RPS7
structural constituent of ribosome
ZFP36L1
3'-UTR-mediated mRNA destabilization (619 aa)
BLASTp hit: XP_005535099.1 [CZME 10D] (647 aa, 57.1% identity)
OCA6
protein tyrosine phosphatase activity (462 aa)
BLASTp hit: XP_005535098.1 [CZME 10D] (510 aa, 81.3% identity)
Apolipoprotein-DUF904 domain
(545 aa)
BLASTp hit: XP_005535097.1 [CZME 10D] (612 aa, 83% query coverage, 39.4% identity)
Coverage
F
R
Iso-seq reads
Conflict
b
1
2,000
4,000
6,000
8,000
10,166
Viralrecall (5 MATCHES)
- VOG04785 [2 hits]: DNA-directed RNA polymerase 147 kDa polypeptide
- VOG01453 [1 hit]: Probable FAD-linked sulfhydryl oxidase E10
- VOG04954 [1 hit]: Probable ubiquitin-conjugating enzyme E2 L460
- VOG05000 [1 hit]: Uncharacterized glycosyltransferase L193
VOG04785
VOG05000
VOG04785
VOG01453
VOG04954
EME26175-1
EME26176-1
EME26177-1
EME26177-2
EME26178-2
EME26178-1
EME26179-1
EME26180-1
Gene
mRNA
Exon
CDS
GASU 074W - scaf_102
Gasu_61750
Gasu_61760
Gasu_61770
Gasu_61780
Gasu_61790
Gasu_61800
Gasu_61810
EME26174-1
EME26175-2
EME26176-2
EME26178-3
EME26180-2
100%
GC (%)
50%
0%
