# Supplementary material for: Genome-wide signatures of adaptation to extreme environments in red algae
Source: Nat Commun. 2023 Jan 4;14:10. doi: 10.1038/s41467-022-35566-x (PMC9812998; doi:10.1038/s41467-022-35566-x)
Supplement: Supplementary file 6 — Source Data [file 41467_2022_35566_MOESM6_ESM.zip › pdf files/Supplementary Figure S12 - ChIP_10D_H3K27me3_subtelomere_221206.pdf]

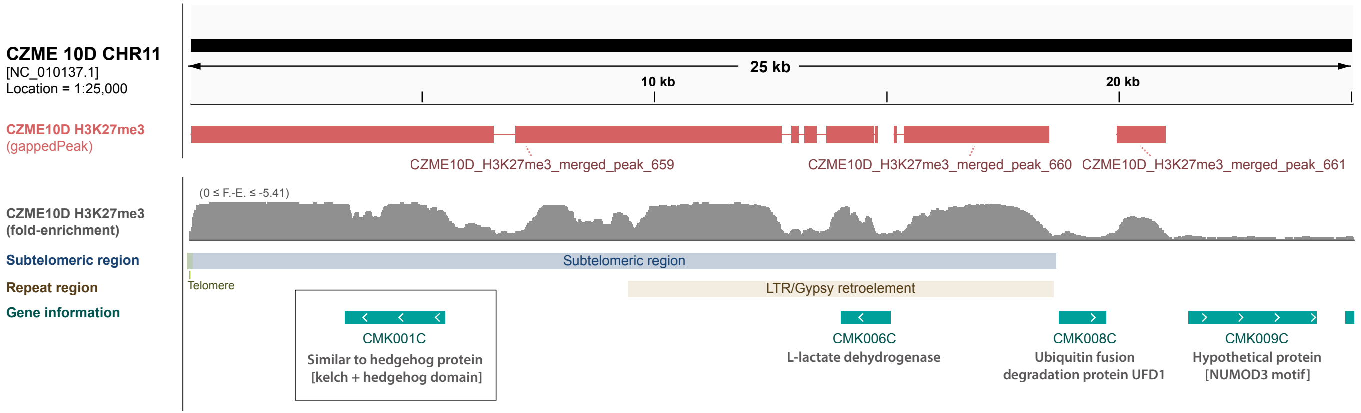
CZME 10D CHR11
[NC_010137.1]
Location = 1:25,000
25 kb
10 kb
20 kb
CZME10D H3K27me3
(gappedPeak)
CZME10D_H3K27me3_merged_peak_659
CZME10D_H3K27me3_merged_peak_660
CZME10D_H3K27me3_merged_peak_661
(0 ≤ F.-E. ≤ -5.41)
CZME10D H3K27me3
(fold-enrichment)
Subtelomeric region
Subtelomeric region
Telomere
Repeat region
LTR/Gypsy retroelement
Gene information
CMK001C
Similar to hedgehog protein
[kelch + hedgehog domain]
CMK006C
L-lactate dehydrogenase
CMK008C
Ubiquitin fusion
degradation protein UFD1
CMK009C
Hypothetical protein
[NUMOD3 motif]
