# Supplementary material for: Genome-wide signatures of adaptation to extreme environments in red algae
Source: Nat Commun. 2023 Jan 4;14:10. doi: 10.1038/s41467-022-35566-x (PMC9812998; doi:10.1038/s41467-022-35566-x)
Supplement: Supplementary file 6 — Source Data [file 41467_2022_35566_MOESM6_ESM.zip › pdf files/Supplementary Figure S11 - Cyanidiales subtelomeric_210817.pdf]

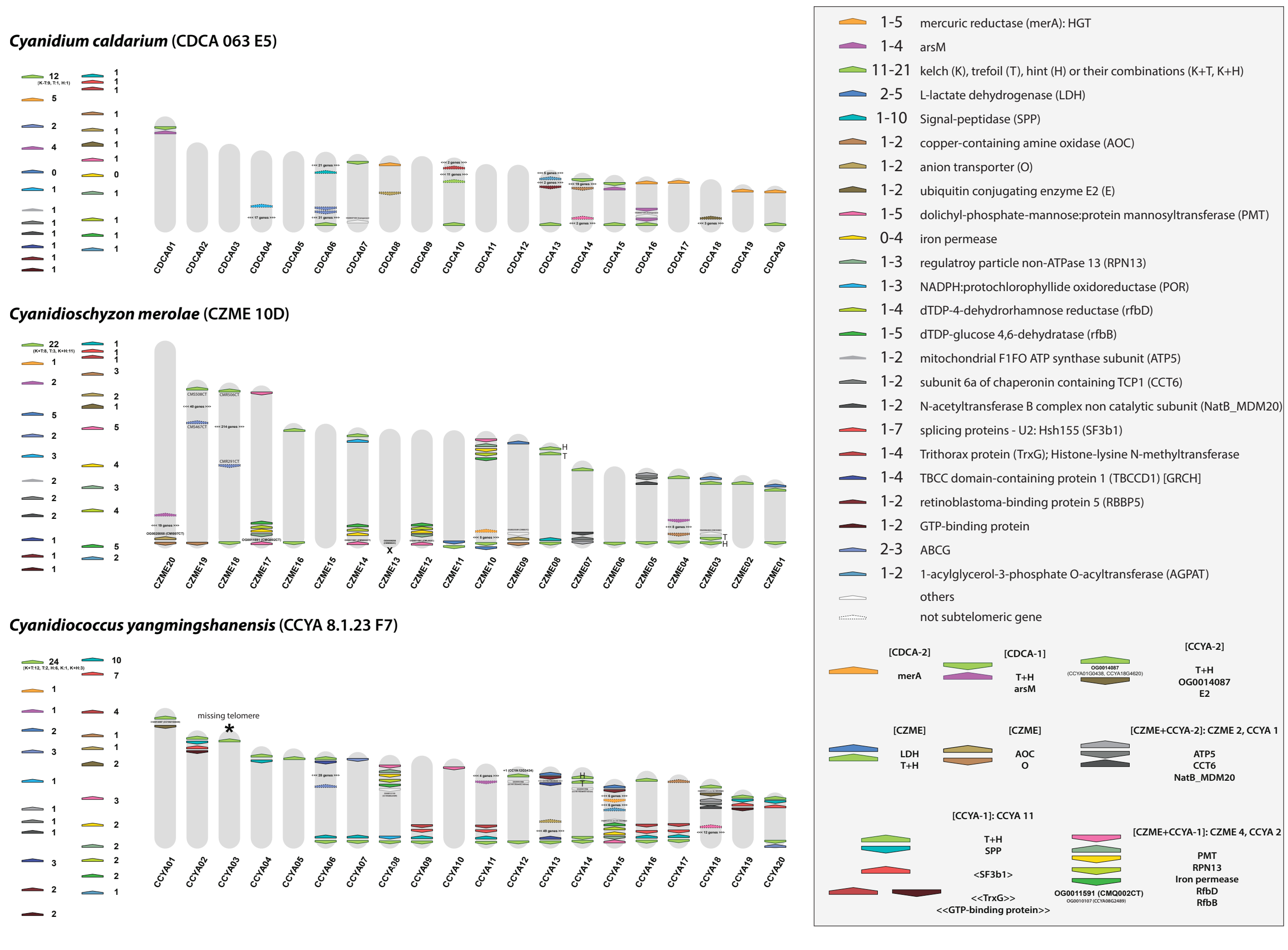

Cyanidium caldarium (CDCA 063 E5)
12
(K-T:9, T:1, H:1)
5
2
4
0
1
1
1
1
1
1
1
1
1
1
1
1
1
1
1
0
1
1
1
1
<<< 17 genes >>>
<<< 21 genes >>>
<<< 31 genes >>>
<<< 2 genes >>>
<<< 11 genes >>>
<<< 5 genes >>>
<<< 2 genes >>>
<<< 19 genes >>>
<<< 2 genes >>>
<<< 3 genes >>>
CDCA01
CDCA02
CDCA03
CDCA04
CDCA05
CDCA06
CDCA07
CDCA08
CDCA09
CDCA10
CDCA11
CDCA12
CDCA13
CDCA14
CDCA15
CDCA16
CDCA17
CDCA18
CDCA19
CDCA20
Cyanidioschyzon merolae (CZME 10D)
22
(K+T:8, T:3, K+H:11)
1
2
5
2
3
2
2
2
1
1
1
1
1
1
3
2
1
5
4
3
4
5
2
CMS508CT
<<< 40 genes >>>
CMS467CT
CMR506CT
<<< 214 genes >>>
CMR291CT
<<< 19 genes >>>
OG0020058 (CMI007CT)
OG0011591 (CMQ002CT)
<<< 5 genes >>>
<<< 8 genes >>>
H
T
T
H
X
CZME20
CZME19
CZME18
CZME17
CZME16
CZME15
CZME14
CZME13
CZME12
CZME11
CZME10
CZME09
CZME08
CZME07
CZME06
CZME05
CZME04
CZME03
CZME02
CZME01
Cyanidiococcus yangmingshanensis (CCYA 8.1.23 F7)
24
(K+T:12, T:2, H:6, K:1, K+H:3)
1
1
2
3
1
1
1
1
3
2
2
10
7
4
1
1
2
3
2
2
2
2
1
missing telomere
*
<<< 28 genes >>>
<<< 4 genes >>>
+1 (CCYA12G3434)
<<< 49 genes >>>
H
T
<<< 6 genes >>>
<<< 6 genes >>>
<<< 12 genes >>>
CCYA01
CCYA02
CCYA03
CCYA04
CCYA05
CCYA06
CCYA07
CCYA08
CCYA09
CCYA10
CCYA11
CCYA12
CCYA13
CCYA14
CCYA15
CCYA16
CCYA17
CCYA18
CCYA19
CCYA20
1-5 mercuric reductase (merA): HGT
1-4 arsM
11-21 kelch (K), trefoil (T), hint (H) or their combinations (K+T, K+H)
2-5 L-lactate dehydrogenase (LDH)
1-10 Signal-peptidase (SPP)
1-2 copper-containing amine oxidase (AOC)
1-2 anion transporter (O)
1-2 ubiquitin conjugating enzyme E2 (E)
1-5 dolichyl-phosphate-mannose:protein mannosyltransferase (PMT)
0-4 iron permease
1-3 regulatroy particle non-ATPase 13 (RPN13)
1-3 NADPH:protochlorophyllide oxidoreductase (POR)
1-4 dTDP-4-dehydrorhamnose reductase (rfbD)
1-5 dTDP-glucose 4,6-dehydratase (rfbB)
1-2 mitochondrial F1FO ATP synthase subunit (ATP5)
1-2 subunit 6a of chaperonin containing TCP1 (CCT6)
1-2 N-acetyltransferase B complex non catalytic subunit (NatB_MDM20)
1-7 splicing proteins - U2: Hsh155 (SF3b1)
1-4 Trithorax protein (TrxG); Histone-lysine N-methyltransferase
1-4 TBCC domain-containing protein 1 (TBCCD1) [GRCH]
1-2 retinoblastoma-binding protein 5 (RBBP5)
1-2 GTP-binding protein
2-3 ABCG
1-2 1-acylglycerol-3-phosphate O-acyltransferase (AGPAT)
others
not subtelomeric gene
[CDCA-2]
merA
[CDCA-1]
T+H
arsM
OG0014087
(CCYA01G0438, CCYA18G4620)
[CCYA-2]
T+H
OG0014087
E2
[CZME]
LDH
T+H
[CZME]
AOC
O
[CZME+CCYA-2]: CZME 2, CCYA 1
ATP5
CCT6
NatB_MDM20
[CCYA-1]: CCYA 11
T+H
SPP
<SF3b1>
<<TrxG>>
<<GTP-binding protein>>
OG0011591 (CMQ002CT)
OG0010107 (CCYA08G2489)
[CZME+CCYA-1]: CZME 4, CCYA 2
PMT
RPN13
Iron permease
RfbD
RfbB
