# Supplementary material for: Genome-wide signatures of adaptation to extreme environments in red algae
Source: Nat Commun. 2023 Jan 4;14:10. doi: 10.1038/s41467-022-35566-x (PMC9812998; doi:10.1038/s41467-022-35566-x)
Supplement: Supplementary file 6 — Source Data [file 41467_2022_35566_MOESM6_ESM.zip › pdf files/Supplementary Figure S21 - population mix_221206.pdf]

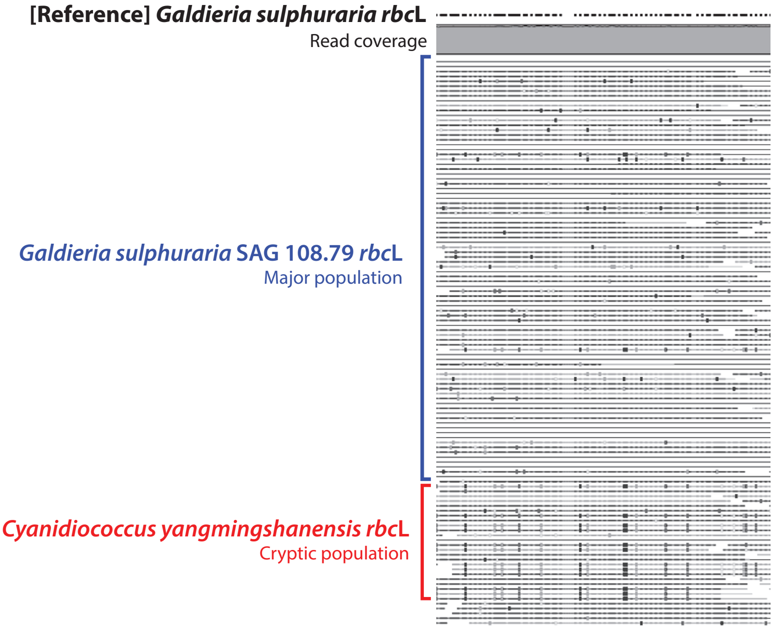

[Reference] Galdieria sulphuraria rbcL
Read coverage
Galdieria sulphuraria SAG 108.79 rbcL
Major population
Cyanidiococcus yangmingshanensis rbcL
Cryptic population
