# Supplementary material for: Genome-wide signatures of adaptation to extreme environments in red algae
Source: Nat Commun. 2023 Jan 4;14:10. doi: 10.1038/s41467-022-35566-x (PMC9812998; doi:10.1038/s41467-022-35566-x)
Supplement: Supplementary file 6 — Source Data [file 41467_2022_35566_MOESM6_ESM.zip › pdf files/Supplementary Figure S10 - STGD:GD ratio boxplot.pdf]

Percentages of subtelomeric gene duplications (STGDs) versus gene duplications

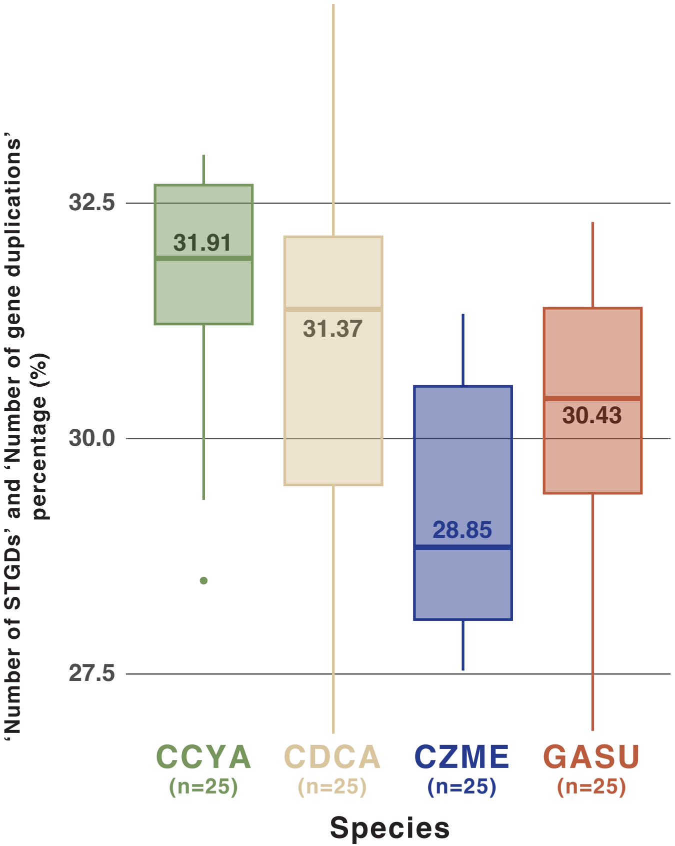

DIAMOND (BLASTp): 25 different parameter combinations were used
- coverage (70, 75, 80, 85, 90%)
- identity (70, 75, 80, 85, 90%)
