# Supplementary material for: Genome-wide signatures of adaptation to extreme environments in red algae
Source: Nat Commun. 2023 Jan 4;14:10. doi: 10.1038/s41467-022-35566-x (PMC9812998; doi:10.1038/s41467-022-35566-x)
Supplement: Supplementary file 6 — Source Data [file 41467_2022_35566_MOESM6_ESM.zip › pdf files/Supplementary Figure S24abc - kmer.pdf]

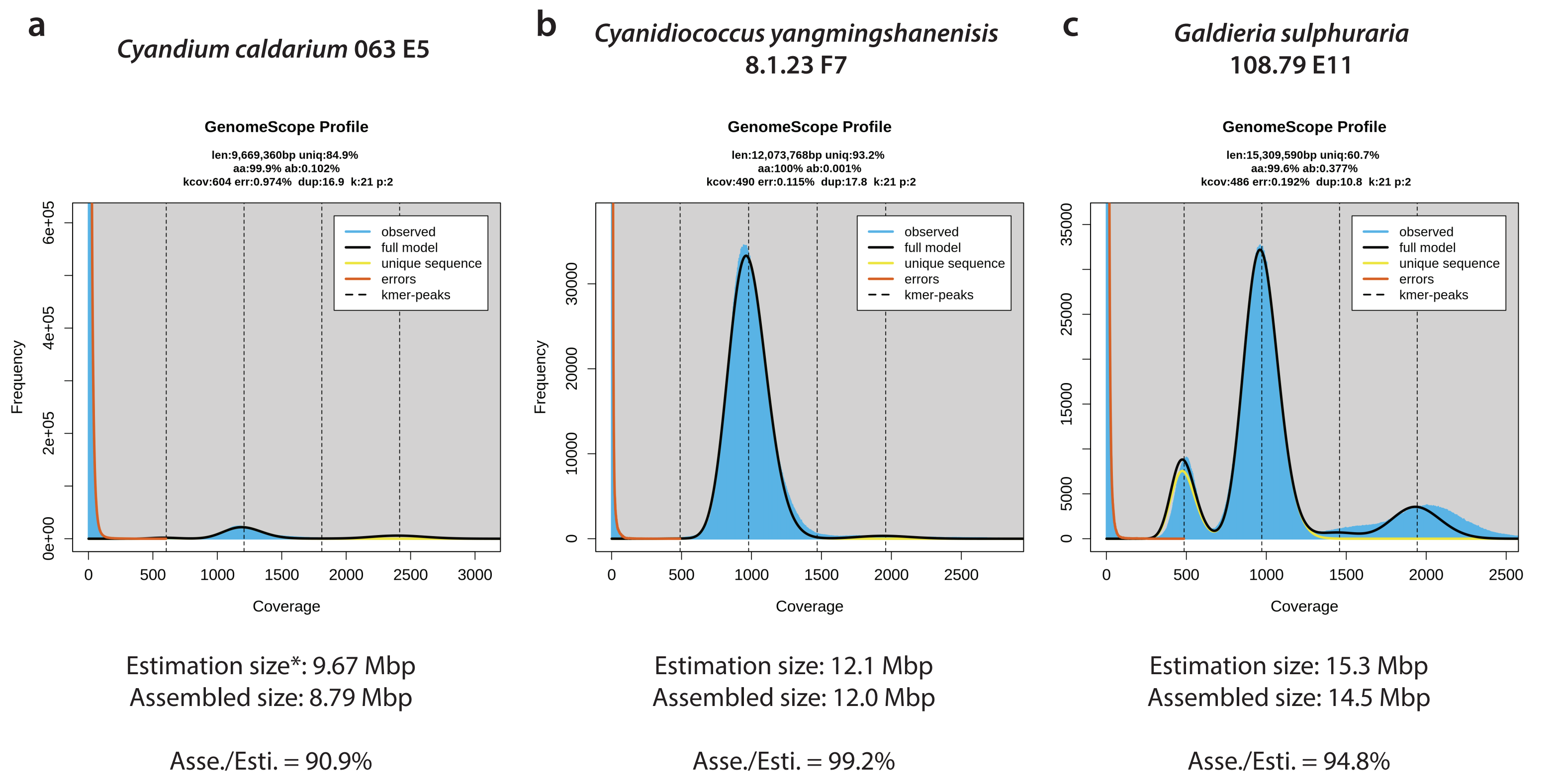

a
Cyandium caldarium 063 E5
GenomeScope Profile
len:9,669,360bp uniq:84.9%
aa:99.9% ab:0.102%
kcov:604 err:0.974% dup:16.9 k:21 p:2
observed
full model
unique sequence
errors
kmer-peaks
Frequency
Coverage
Estimation size*: 9.67 Mbp
Assembled size: 8.79 Mbp
Asse./Esti. = 90.9%
b
Cyanidiococcus yangmingshanenisis
8.1.23 F7
GenomeScope Profile
len:12,073,768bp uniq:93.2%
aa:100% ab:0.001%
kcov:490 err:0.115% dup:17.8 k:21 p:2
observed
full model
unique sequence
errors
kmer-peaks
Frequency
Coverage
Estimation size: 12.1 Mbp
Assembled size: 12.0 Mbp
Asse./Esti. = 99.2%
c
Galdieria sulphuraria
108.79 E11
GenomeScope Profile
len:15,309,590bp uniq:60.7%
aa:99.6% ab:0.377%
kcov:486 err:0.192% dup:10.8 k:21 p:2
observed
full model
unique sequence
errors
kmer-peaks
Coverage
Estimation size: 15.3 Mbp
Assembled size: 14.5 Mbp
Asse./Esti. = 94.8%
