# Supplementary figures and images for: Genome-wide signatures of adaptation to extreme environments in red algae
Source: Nat Commun. 2023 Jan 4;14:10. doi: 10.1038/s41467-022-35566-x (PMC9812998; doi:10.1038/s41467-022-35566-x)

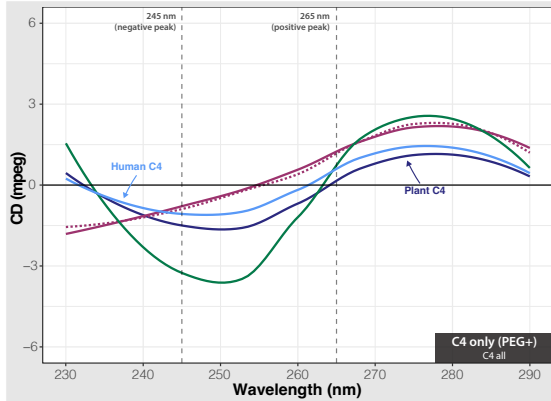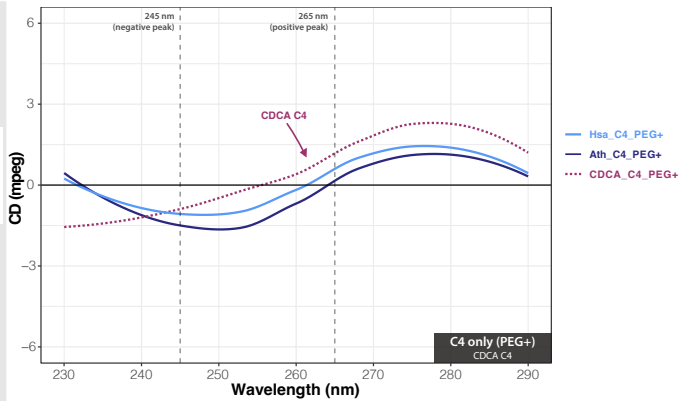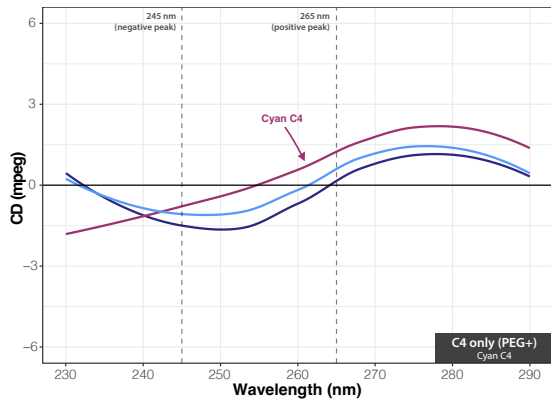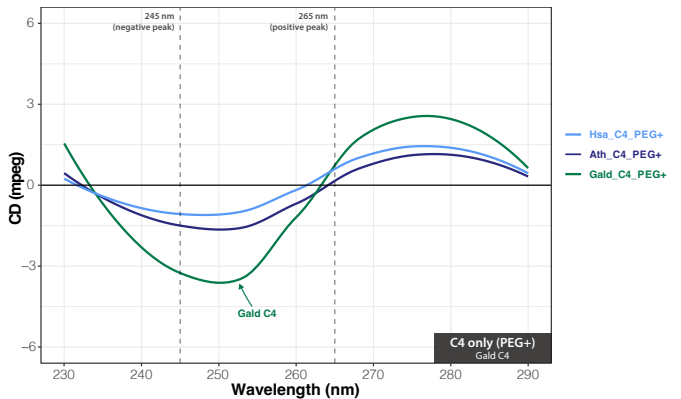

Supplement: Supplementary file 6 — Source Data [file 41467_2022_35566_MOESM6_ESM.zip › pdf files/Supplementary Figure S4 - C4 CD spectra.pdf]

# PROKARYOTES [421 taxa]

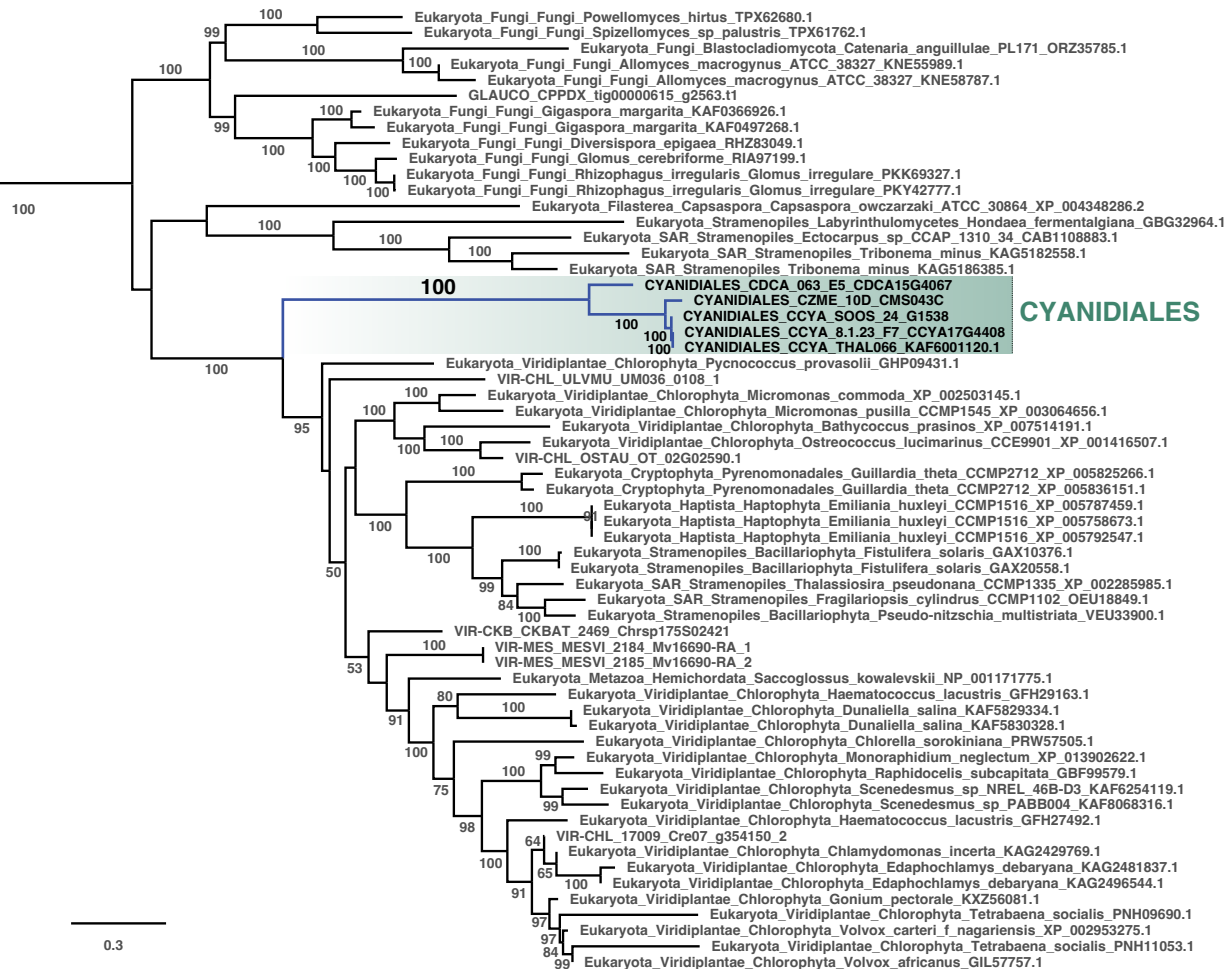

Supplement: Supplementary file 6 — Source Data [file 41467_2022_35566_MOESM6_ESM.zip › pdf files/Supplementary Figure S14 - arsJ phylogeny_221206.pdf]

**[REFERENCE]**  
*Galdieria sulphuraria*  
MtSh

**[NEW]**  
*Galdieria sulphuraria*  
108.79 E11

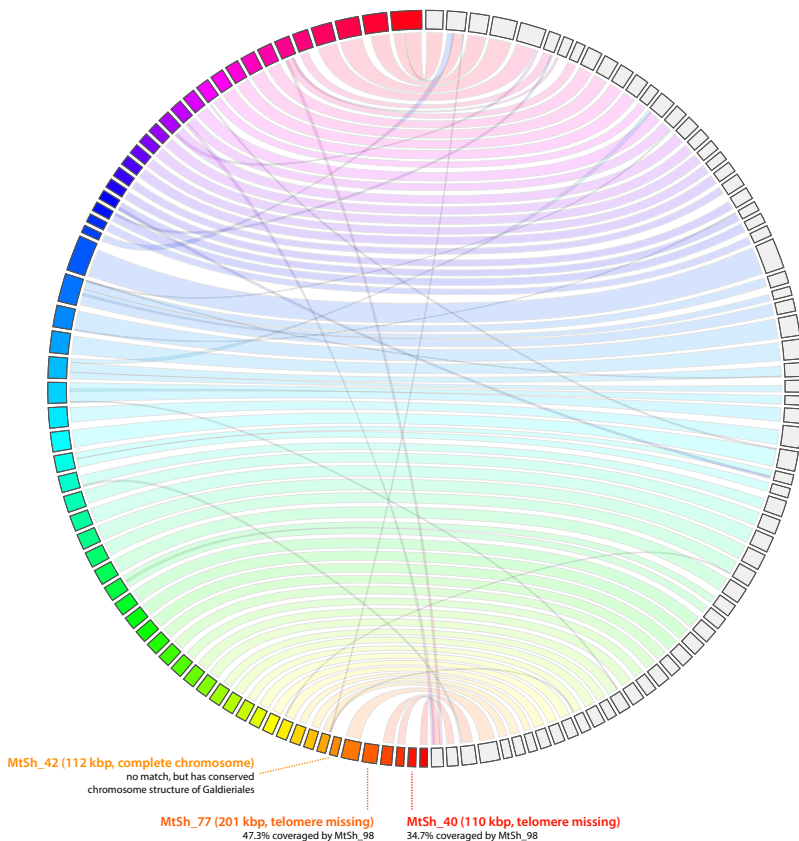

Supplement: Supplementary file 6 — Source Data [file 41467_2022_35566_MOESM6_ESM.zip › pdf files/Supplementary Figure S5 - GASU_108.79_E11_vs_MtSH_221109.pdf]

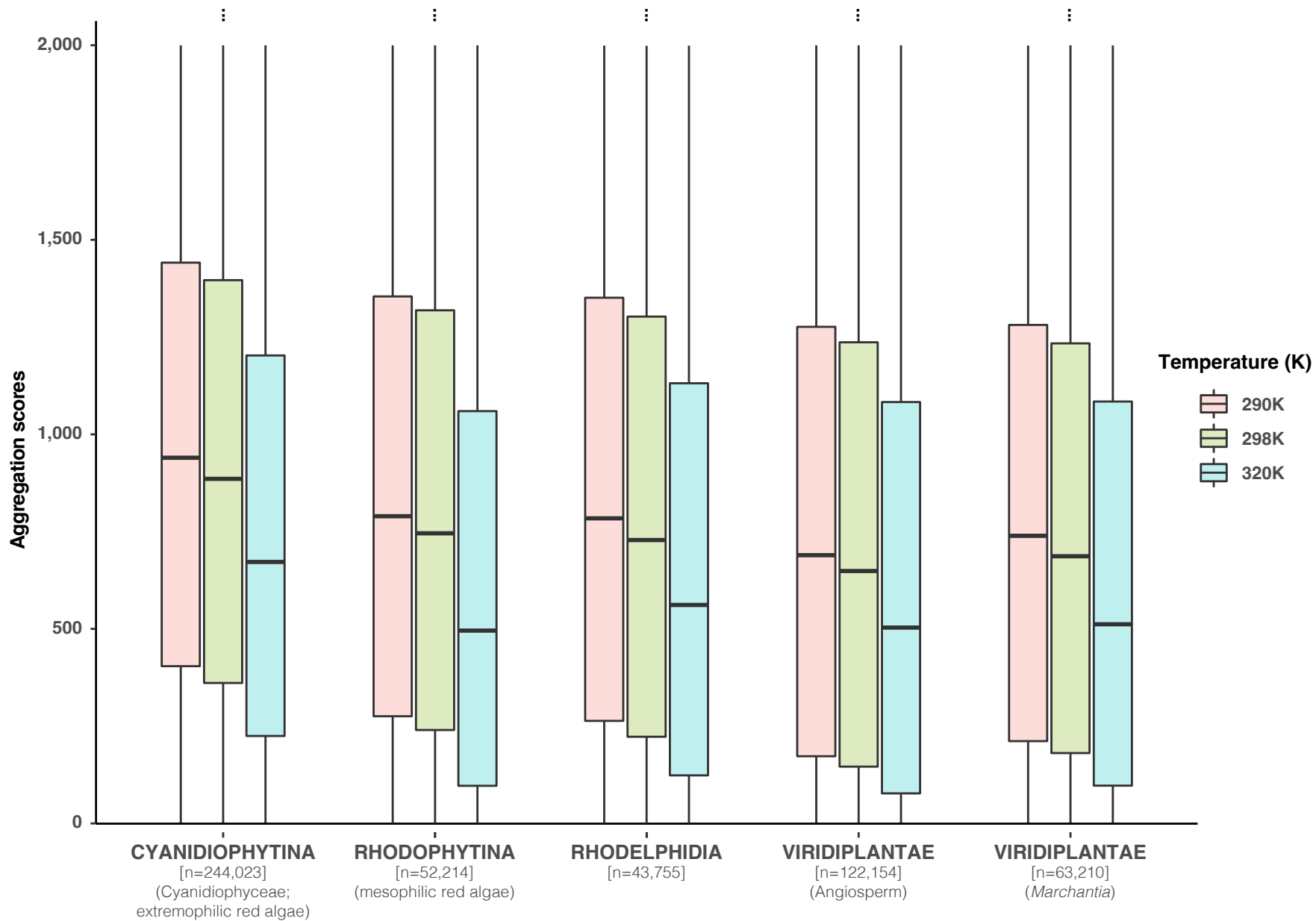

Supplement: Supplementary file 6 — Source Data [file 41467_2022_35566_MOESM6_ESM.zip › pdf files/Supplementary Figure S19 - aggregation prone 221117.pdf]

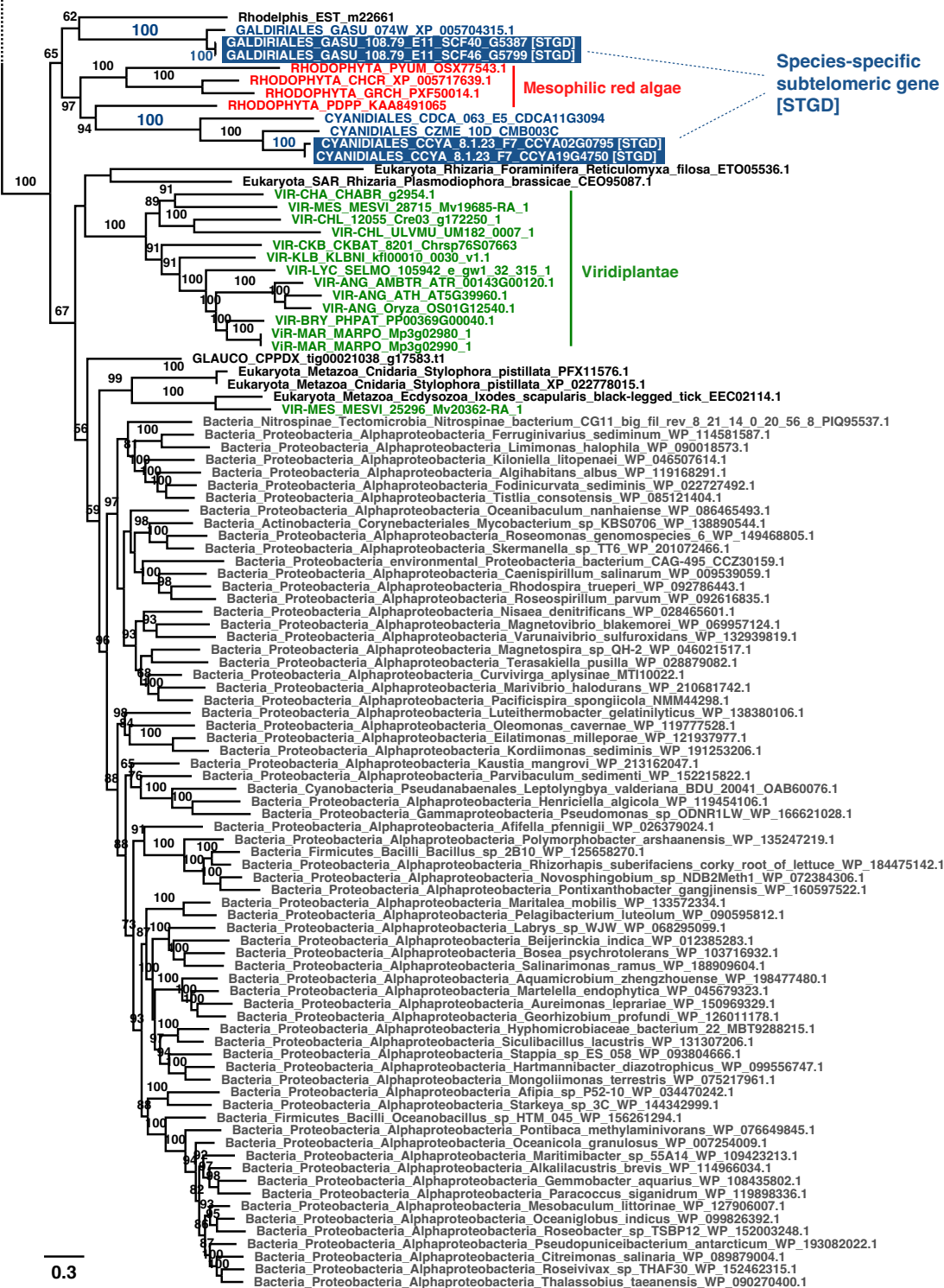

Supplement: Supplementary file 6 — Source Data [file 41467_2022_35566_MOESM6_ESM.zip › pdf files/Supplementary Figure S9 - independent STGD of GTP binding protein .pdf]

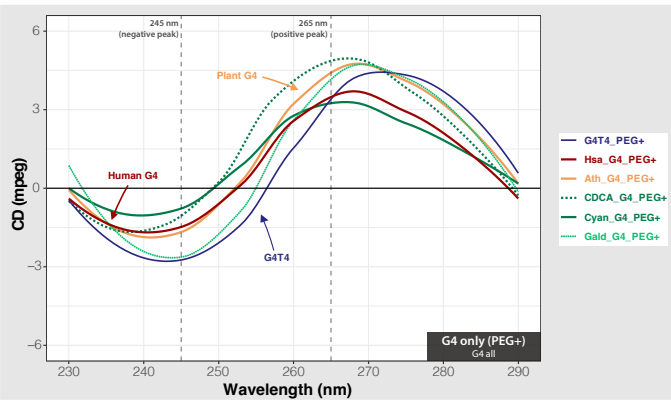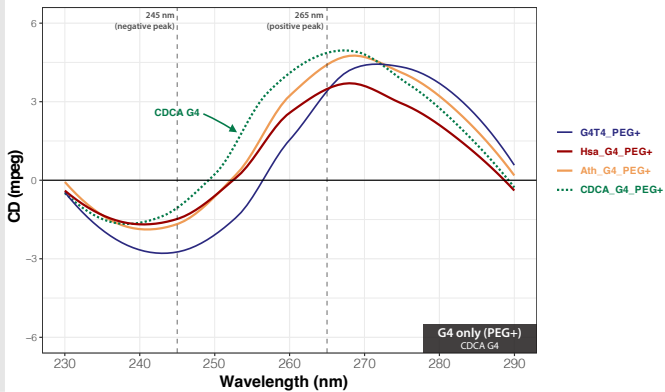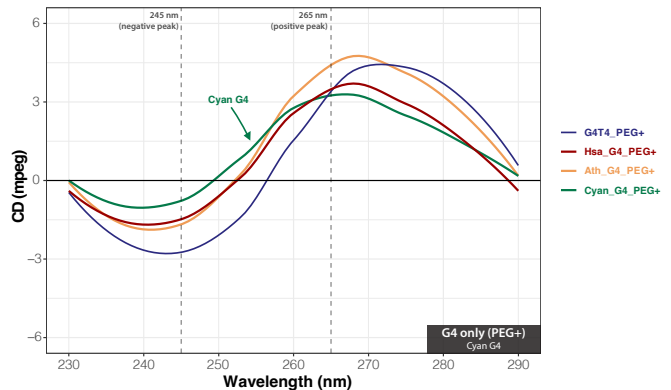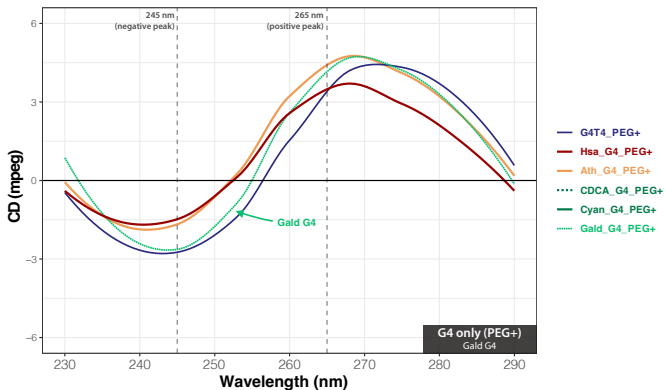

Supplement: Supplementary file 6 — Source Data [file 41467_2022_35566_MOESM6_ESM.zip › pdf files/Supplementary Figure S3 - G4 CD spectra.pdf]

**a**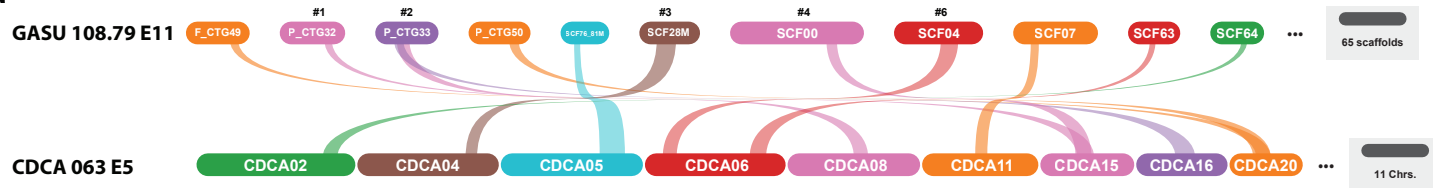**b**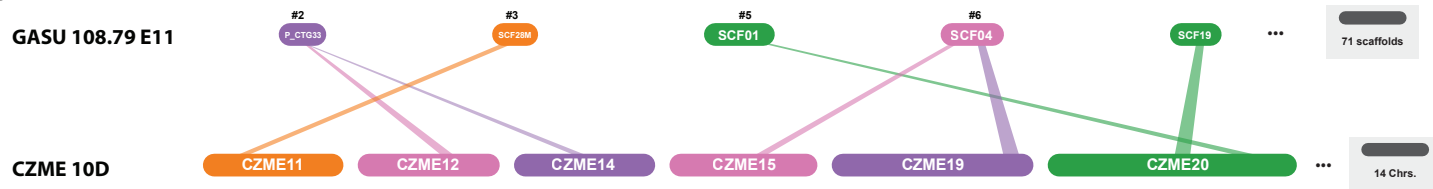**c**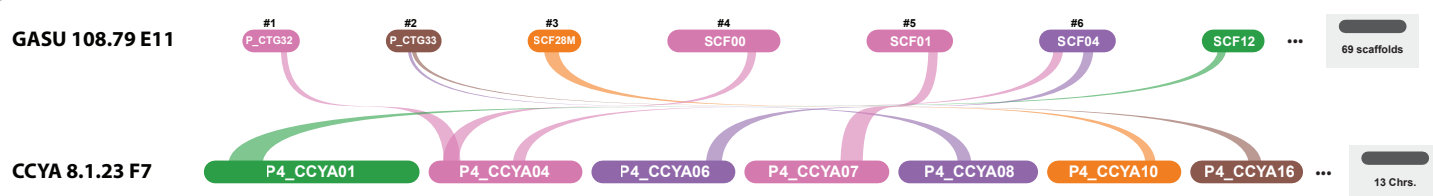

Supplement: Supplementary file 6 — Source Data [file 41467_2022_35566_MOESM6_ESM.zip › pdf files/Supplementary Figure S6abc - GASU_vs_CYANIDIALES chr.pdf]

a

## CCYA 8.1.23 F7 - Chromosome 4

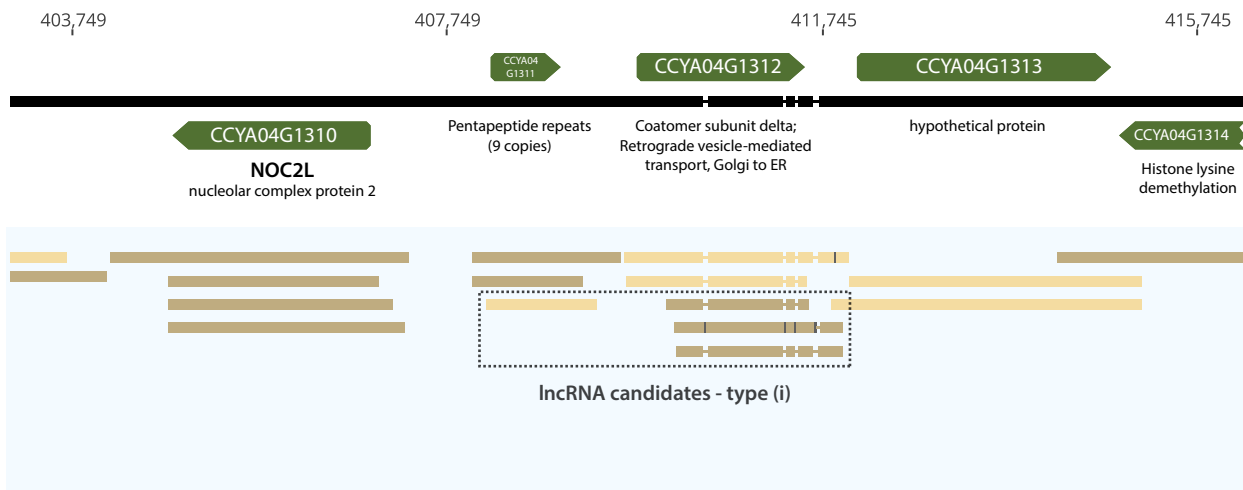

b

## CDCA 063 E5 - Chromosome 17

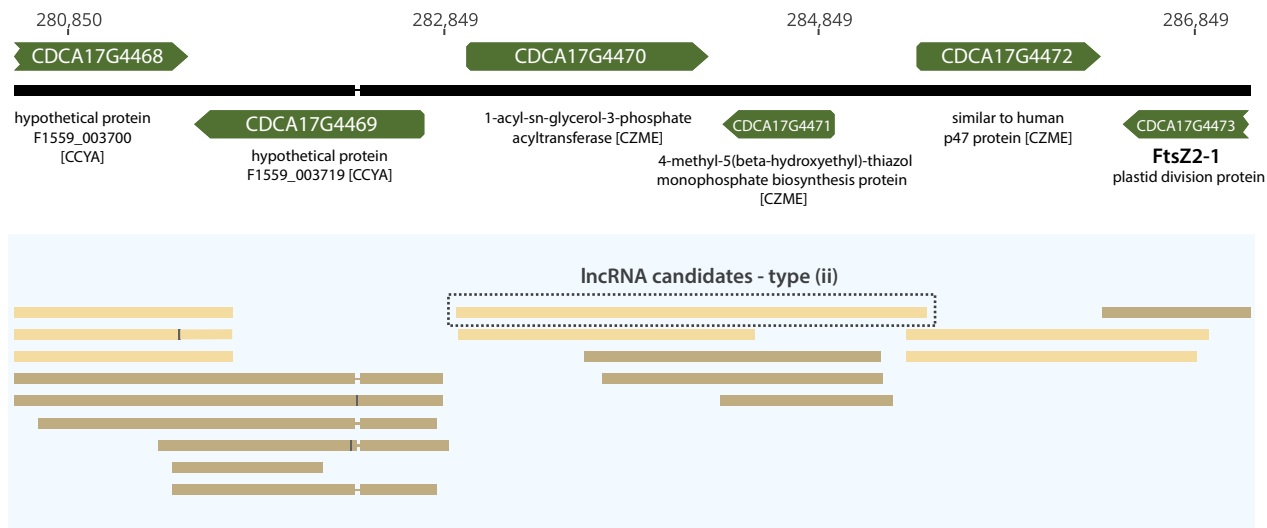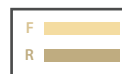

Iso-seq reads

Supplement: Supplementary file 6 — Source Data [file 41467_2022_35566_MOESM6_ESM.zip › pdf files/Supplementary Figure S16ab - LNC_possible_candidate.pdf]

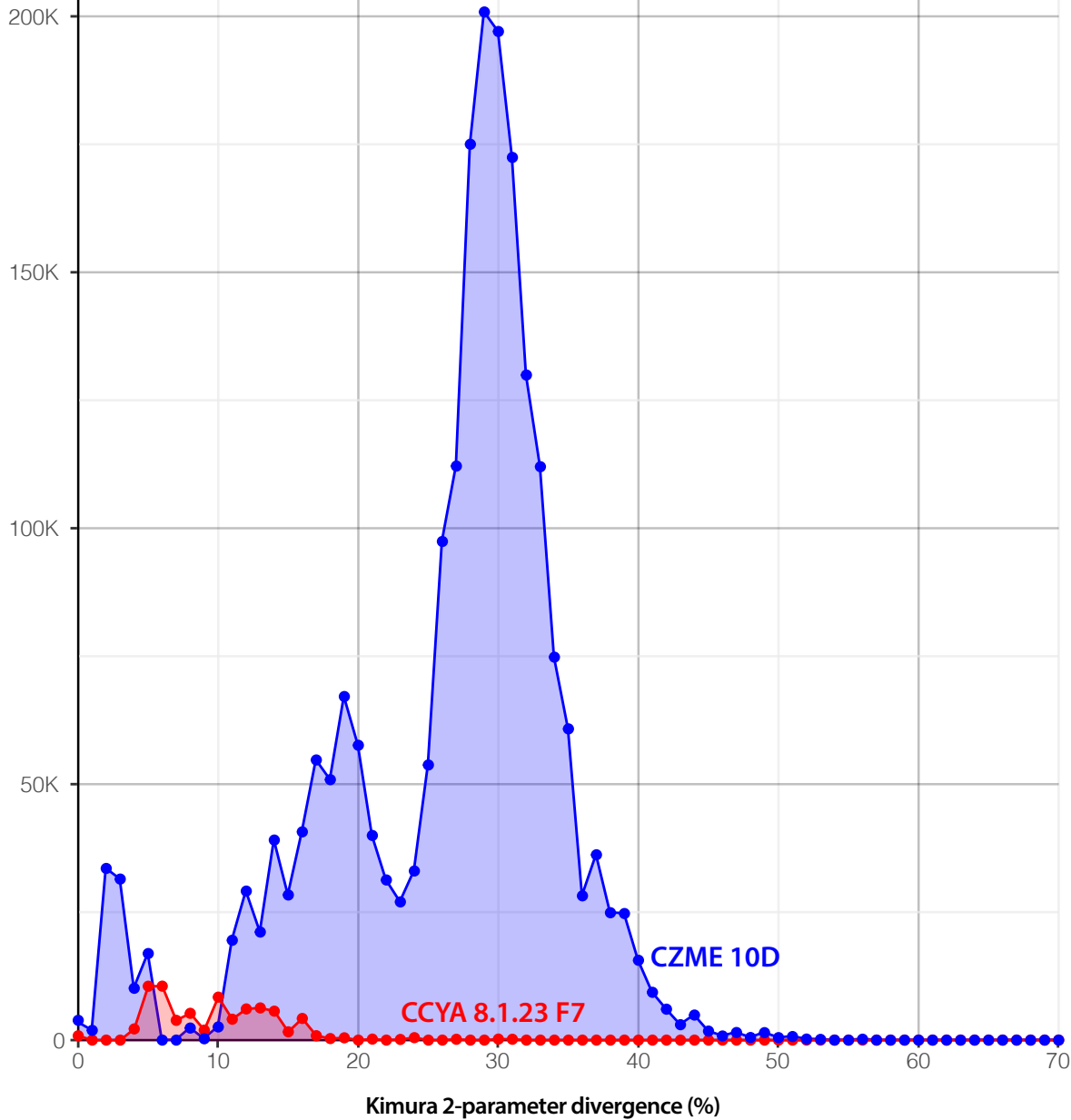

Supplement: Supplementary file 6 — Source Data [file 41467_2022_35566_MOESM6_ESM.zip › pdf files/Supplementary Figure S7 - CCYA CZME LTR.pdf]

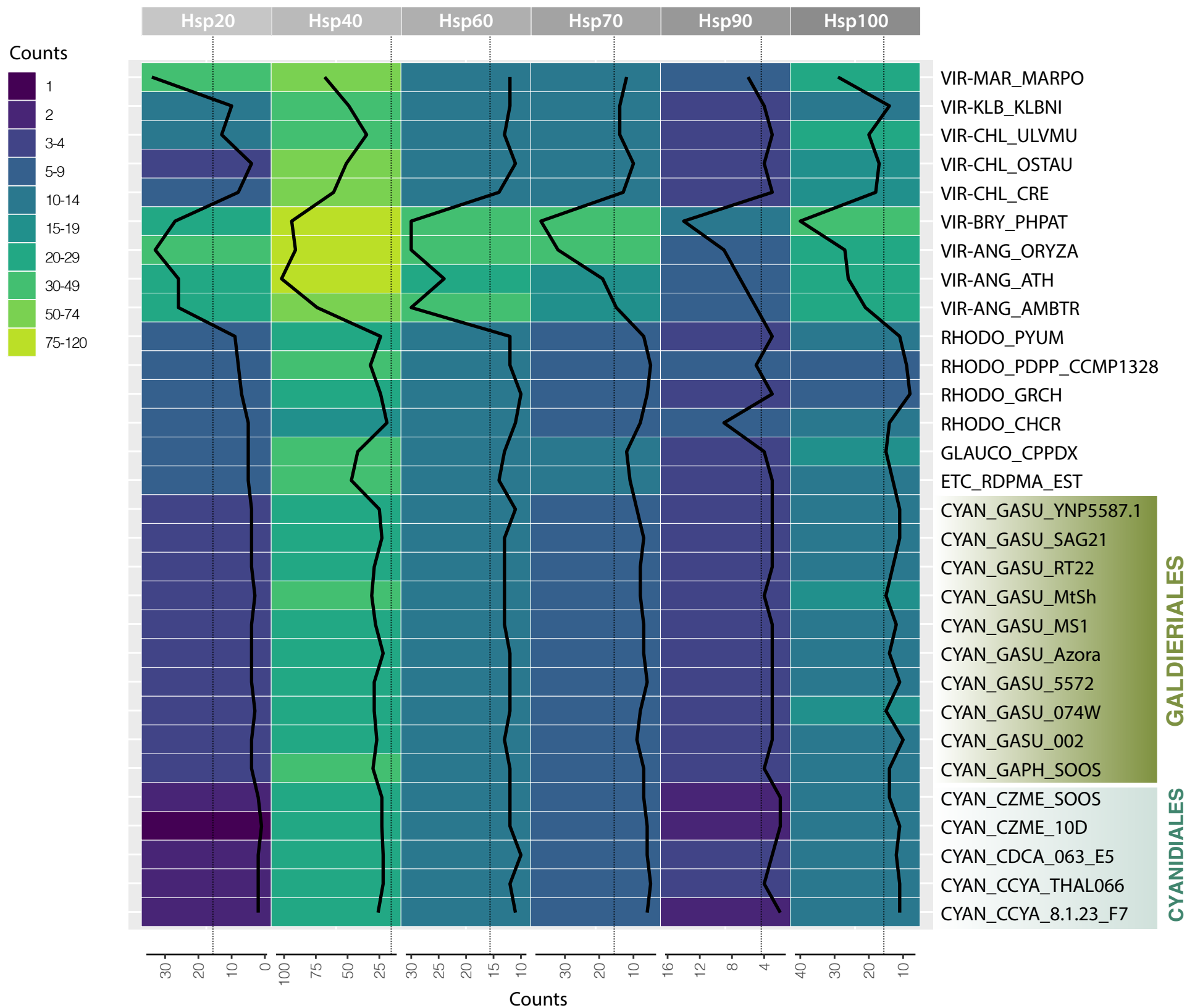

Supplement: Supplementary file 6 — Source Data [file 41467_2022_35566_MOESM6_ESM.zip › pdf files/Supplementary Figure S18 - HSP_dotplot.pdf]

## CDCA 063 E5 - Chromosome 3

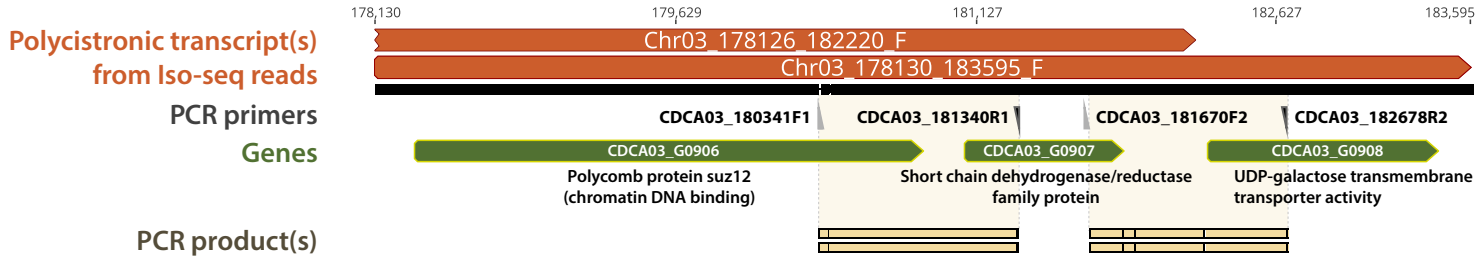

## CDCA 063 E5 - Chromosome 7

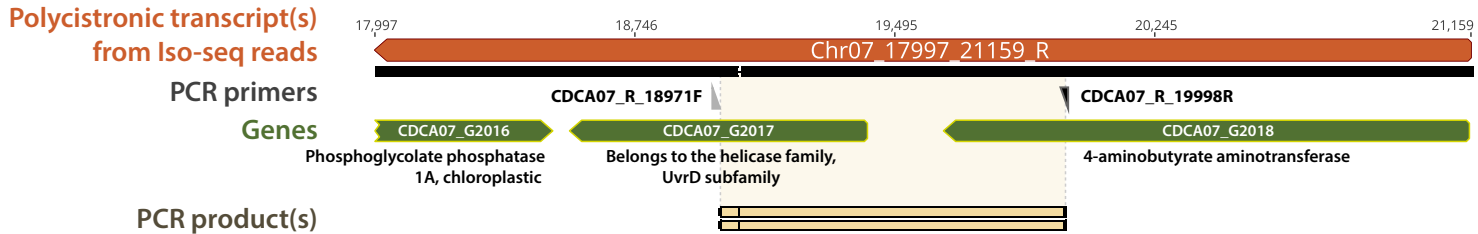

## CDCA 063 E5 - Chromosome 13

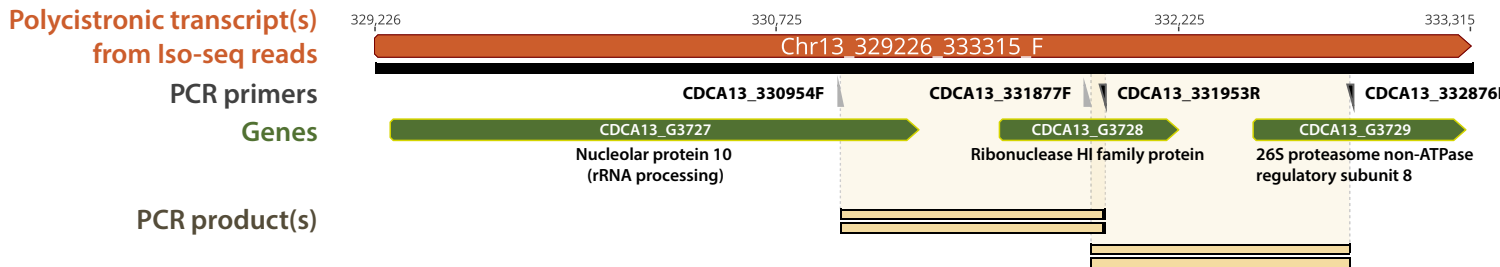

Supplement: Supplementary file 6 — Source Data [file 41467_2022_35566_MOESM6_ESM.zip › pdf files/Supplementary Figure S20 - CDCA_polycistronic_transcripts.pdf]
