# Supplementary material for: Genome-wide signatures of adaptation to extreme environments in red algae
Source: Nat Commun. 2023 Jan 4;14:10. doi: 10.1038/s41467-022-35566-x (PMC9812998; doi:10.1038/s41467-022-35566-x)
Supplement: Supplementary file 6 — Source Data [file Supplementary Figure S8abcd - Cyanidiophyceae Chromsome 221117.pdf › pdf files/Supplementary Figure S8abcd - Cyanidiophyceae Chromsome 221117.pdf]

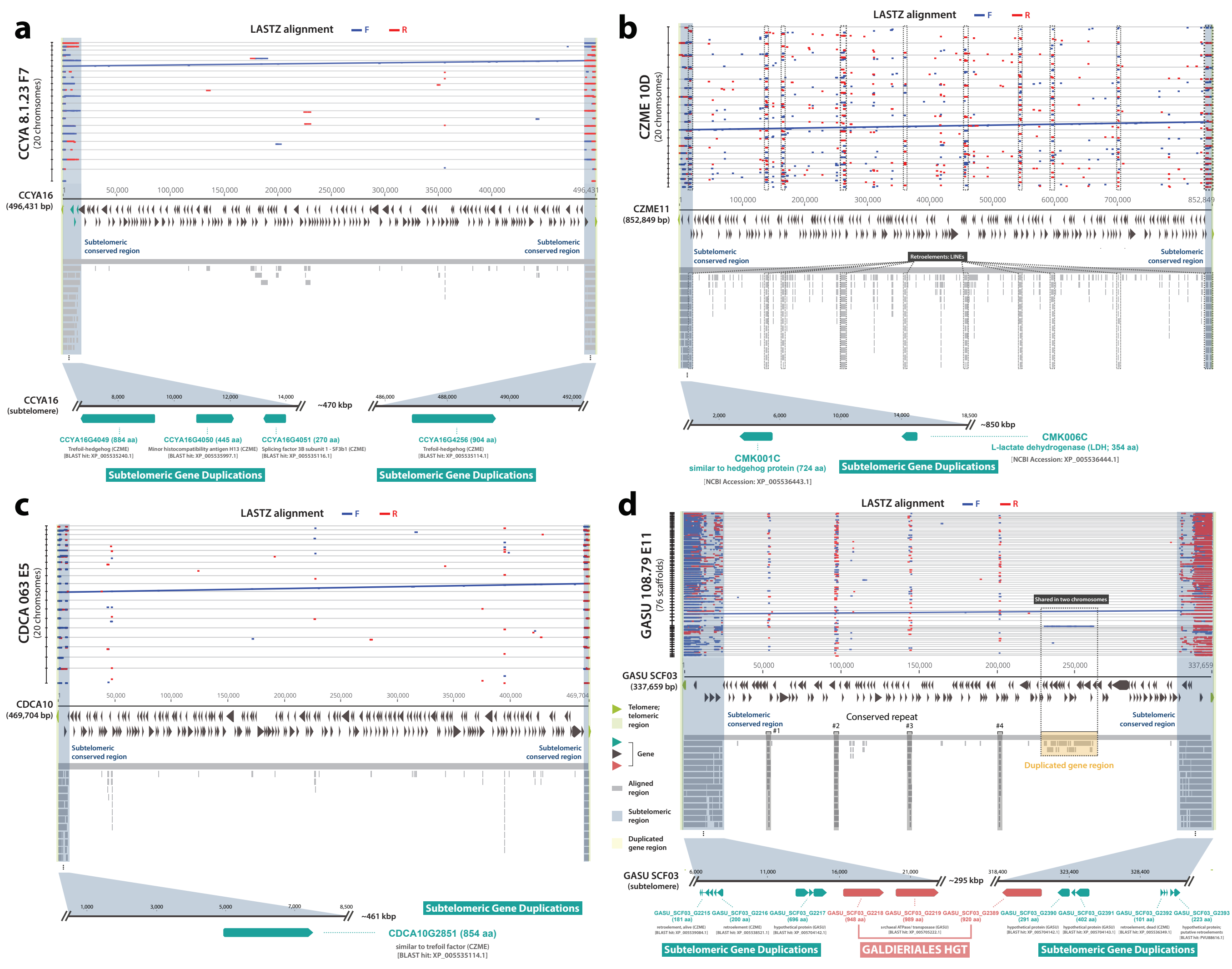
a
LASTZ alignment
F
R
CCYA 8.1.23 F7
(20 chromsomes)
CCYA16
(496,431 bp)
Subtelomeric conserved region
Subtelomeric conserved region
CCYA16 (subtelomere)
~470 kbp
CCYA16G4049 (884 aa)
Trefoil-hedgehog (CZME)
[BLAST hit: XP_005535240.1]
CCYA16G4050 (445 aa)
Minor histocompatibility antigen H13 (CZME)
[BLAST hit: XP_005535997.1]
CCYA16G4051 (270 aa)
Splicing factor 3B subunit 1 - SF3b1 (CZME)
[BLAST hit: XP_005535116.1]
CCYA16G4256 (904 aa)
Trefoil-hedgehog (CZME)
[BLAST hit: XP_005535114.1]
Subtelomeric Gene Duplications
Subtelomeric Gene Duplications
b
LASTZ alignment
F
R
CZME 10D
(20 chromsomes)
CZME11
(852,849 bp)
Subtelomeric conserved region
Retroelements: LINEs
Subtelomeric conserved region
~850 kbp
CMK001C
similar to hedgehog protein (724 aa)
[NCBI Accession: XP_005536443.1]
Subtelomeric Gene Duplications
CMK006C
L-lactate dehydrogenase (LDH; 354 aa)
[NCBI Accession: XP_005536444.1]
c
LASTZ alignment
F
R
CDCA 063 E5
(20 chromsomes)
CDCA10
(469,704 bp)
Subtelomeric conserved region
Subtelomeric conserved region
~461 kbp
Subtelomeric Gene Duplications
CDCA10G2851 (854 aa)
similar to trefoil factor (CZME)
[BLAST hit: XP_005535114.1]
d
LASTZ alignment
F
R
GASU 108.79 E11
(76 scaffolds)
Shared in two chromosomes
GASU SCF03
(337,659 bp)
Telomere; telomeric region
Gene
Aligned region
Subtelomeric region
Duplicated gene region
Subtelomeric conserved region
Conserved repeat
Subtelomeric conserved region
Duplicated gene region
GASU SCF03 (subtelomere)
~295 kbp
GASU_SCF03_G2215 (181 aa)
GASU_SCF03_G2216 (200 aa)
GASU_SCF03_G2217 (696 aa)
GASU_SCF03_G2218 (948 aa)
GASU_SCF03_G2219 (989 aa)
GASU_SCF03_G2389 (920 aa)
GASU_SCF03_G2390 (291 aa)
GASU_SCF03_G2391 (402 aa)
GASU_SCF03_G2392 (101 aa)
GASU_SCF03_G2393 (223 aa)
archaeal ATPase/ transposase (GASU)
Subtelomeric Gene Duplications
GALDIERIALES HGT
Subtelomeric Gene Duplications
